# Genome-wide association meta-analysis of corneal curvature identifies novel loci and shared genetic influences across axial length and refractive error

**DOI:** 10.1038/s42003-020-0802-y

**Published:** 2020-03-19

**Authors:** Qiao Fan, Alfred Pozarickij, Nicholas Y. Q. Tan, Xiaobo Guo, Virginie J. M. Verhoeven, Veronique Vitart, Jeremy A. Guggenheim, Masahiro Miyake, J. Willem L. Tideman, Anthony P. Khawaja, Liang Zhang, Stuart MacGregor, René Höhn, Peng Chen, Ginevra Biino, Juho Wedenoja, Seyed Ehsan Saffari, Milly S. Tedja, Jing Xie, Carla Lanca, Ya Xing Wang, Srujana Sahebjada, Johanna Mazur, Alireza Mirshahi, Nicholas G. Martin, Seyhan Yazar, Craig E. Pennell, Maurice Yap, Annechien E. G. Haarman, Clair A. Enthoven, JanRoelof Polling, Consortium for Refractive Error and Myopia (CREAM), Joan E. Bailey-Wilson, Amutha Barathi Veluchamy, Kathryn P. Burdon, Harry Campbell, Li Jia Chen, Emily Y. Chew, Jamie E. Craig, Phillippa M. Cumberland, Margaret M. Deangelis, Cécile Delcourt, Xiaohu Ding, David M. Evans, Puya Gharahkhani, Adriana I. Iglesias, Toomas Haller, Xikun Han, Quan Hoang, Robert P. Igo, Sudha K. Iyengar, Mika Kähönen, Jaakko Kaprio, Barbara E. Klein, Ronald Klein, Jonathan H. Lass, Kris Lee, Terho Lehtimäki, Deyana D. Lewis, Qing Li, Shi-Ming Li, Leo-Pekka Lyytikäinen, Akira Meguro, Andres Metspalu, Candace D. Middlebrooks, Nobuhisa Mizuki, Anthony M. Musolf, Stefan Nickels, Konrad Oexle, Chi Pui Pang, Andrew D. Paterson, Jugnoo S. Rahi, Olli Raitakari, Igor Rudan, Dwight Stambolian, Claire L. Simpson, Ningli Wang, Wen Bin Wei, Katie M. Williams, James F. Wilson, Robert Wojciechowski, Kenji Yamashiro, Jason C. S. Yam, Xiangtian Zhou, UK Biobank Eye and Vision Consortium, Tariq Aslam, Sarah A. Barman, Jenny H. Barrett, Paul Bishop, Peter Blows, Catey Bunce, Roxana O. Carare, Usha Chakravarthy, Michelle Chan, Sharon Y. L. Chua, David P. Crabb, Philippa M. Cumberland, Alexander Day, Parul Desai, Bal Dhillon, Andrew D. Dick, Cathy Egan, Sarah Ennis, Marcus Fruttiger, John E. J. Gallacher, David F. Garway-Heath, Jane Gibson, Dan Gore, Alison Hardcastle, Simon P. Harding, Ruth E. Hogg, Pearse A. Keane, Sir Peng T. Khaw, Gerassimos Lascaratos, Andrew J. Lotery, Tom Macgillivray, Sarah Mackie, Keith Martin, Michelle McGaughey, Bernadette McGuinness, Gareth J. McKay, Martin McKibbin, Danny Mitry, Tony Moore, James E. Morgan, Zaynah A. Muthy, Eoin O’Sullivan, Chris G. Owen, Praveen Patel, Euan Paterson, Tunde Peto, Axel Petzold, Jugnoo S. Rahi, Alicja R. Rudnikca, Jay Self, Sobha Sivaprasad, David Steel, Irene Stratton, Nicholas Strouthidis, Cathie Sudlow, Dhanes Thomas, Emanuele Trucco, Adnan Tufail, Stephen A. Vernon, Ananth C. Viswanathan, Katie Williams, Jayne V. Woodside, Max M. Yates, Jennifer Yip, Yalin Zheng, Alex W. Hewitt, Vincent W. V. Jaddoe, Cornelia M. van Duijn, Caroline Hayward, Ozren Polasek, E-Shyong Tai, Hosoda Yoshikatsu, Pirro G. Hysi, Terri L. Young, Akitaka Tsujikawa, Jie Jing Wang, Paul Mitchell, Norbert Pfeiffer, Olavi Pärssinen, Paul J. Foster, Maurizio Fossarello, Shea Ping Yip, Cathy Williams, Christopher J. Hammond, Jost B. Jonas, Mingguang He, David A. Mackey, Tien-Yin Wong, Caroline C. W. Klaver, Seang-Mei Saw, Paul N. Baird, Ching-Yu Cheng

**Affiliations:** 1 Centre for Quantitative Medicine, Duke-NUS Medical School, 20 College Road, 169856 Singapore, Singapore; 2 Ophthalmology & Visual Sciences Academic Clinical Program (Eye ACP), Duke-NUS Medical School, Singapore, Singapore; 3 School of Optometry and Vision Sciences, Cardiff University, Cardiff, CF24 4HQ UK; 4 Singapore Eye Research Institute, Singapore National Eye Centre, Singapore, Singapore; 5 Department of Statistical Science, School of Mathematics, Sun Yat-Sen University, Guangzhou, China; 6 Southern China Center for Statistical Science, Sun Yat-Sen University, Guangzhou, China; 7 Department of Ophthalmology, Erasmus Medical Center, 3000 CA Rotterdam, The Netherlands; 8 Department of Clinical Genetics, Erasmus Medical Center, Rotterdam, the Netherlands; 9 Medical Research Council Human Genetics Unit, Institute of Genetics and Molecular Medicine, University of Edinburgh, Edinburgh, EH4 2XU UK; 10 Department of Ophthalmology and Visual Sciences, Kyoto University, Kyoto, 6068507 Japan; 11 Department of Epidemiology, Erasmus Medical Center, 3000 CA Rotterdam, The Netherlands; 12 NIHR Biomedical Research Centre, Moorfields Eye Hospital NHS Foundation Trust and UCL Institute of Ophthalmology, London, EC1V 2PD UK; 13 Department of Public Health and Primary Care, Institute of Public Health, University of Cambridge School of Clinical Medicine, Cambridge, UK; 14 Ocular Epidemiology Research Group, Singapore Eye Research Institute, Singapore, Singapore; 15 QIMR Berghofer Medical Research Institute, Brisbane, Australia; 16 Department of Ophthalmology, University Medical Center of the Johannes Gutenberg-University Mainz, 55131 Mainz, Germany; 17 Department of Ophthalmology, Inselspital, University Hospital Bern, University of Bern, Bern, 3010 Switzerland; 18 Saw Swee Hock School of Public Health, National University Health Systems, National University of Singapore, Singapore, Singapore; 19 Institute of Molecular Genetics, National Research Council of Italy, Pavia, 27100 Italy; 20 Department of Ophthalmology, University of Helsinki and Helsinki University Hospital, Helsinki, FI-00290 Finland; 21 Department of Public Health, University of Helsinki, Helsinki, FI-00290 Finland; 22 Occupational and Environmental Health Sciences, School of Public Health and Preventative Medicine, Monash University, Melbourne, 3004 Australia; 23 Centre for Eye Research Australia (CERA), University of Melbourne, Royal Victorian Eye and Ear Hospital, Melbourne, 3002 Australia; 24 Myopia Research Group, Singapore Eye Research Institute, Singapore, Singapore; 25 Beijing Institute of Ophthalmology, Beijing Key Laboratory of Ophthalmology and Visual Sciences, Beijing Tongren Eye Center, Beijing Tongren Hospital, Capital Medical University, Beijing, China; 26 Department of Surgery, Ophthalmology, Faculty of Medicine, Dentistry and Health Sciences, The University of Melbourne, Royal Victorian Eye and Ear Hospital, Melbourne, 3002 Australia; 27 Institute for Medical Biostatistics, Epidemiology and Informatics, University Medical Center of the Johannes Gutenberg-University Mainz, 55131 Mainz, Germany; 28 Dardenne Eye Hospital, Bonn-Bad Godesberg, Godesberg, 53117 Germany; 29 Centre for Ophthalmology and Visual Science, University of Western Australia, Lions Eye Institute, Perth, Australia; 30 School of Women’s and Infants’ Health, The University of Western Australia, Perth, WA 6009 Australia; 31 School of Optometry, The Hong Kong Polytechnic University, Hong Kong SAR, China; 32 Orthoptics & Optometry, University of Applied Sciences, Utrecht, Netherlands; 33 Menzies Institute for Medical Research, School of Medicine, University of Tasmania, Hobart, Australia; 34 Generation R Study Group, Erasmus MC, University Medical Center, Rotterdam, the Netherlands; 35 Faculty of Medicine, University of Split, Croatia, Split, 21000 Croatia; 36 Section of Academic Ophthalmology, School of Life Course Sciences, King’s College London, London, UK; 37 Department of Ophthalmology and Visual Sciences, University of Wisconsin-Madison, Madison, WI USA; 38 Duke-NUS Medical School, Singapore, Singapore; 39 Centre for Vision Research, Department of Ophthalmology and Westmead Institute for Medical Research, University of Sydney, Sydney, NSW 2145 Australia; 40 Gerontology Research Center and Faculty of Sport and Health Sciences, University of Jyväskylä, Jyväskylä, FI-40100 Finland; 41 Department of Ophthalmology, Central Hospital of Central Finland, Jyväskylä, FI-40100 Finland; 42 San Giovanni di Dio hospital, Clinica Oculistica, Azienda Ospedaliera Universitaria di Cagliari, Cagliari, 09131 Italy; 43 Department of Surgical Sciences, Eye Clinic, University of Cagliari, Cagliari, 09131 Italy; 44 Department of Health Technology and Informatics, The Hong Kong Polytechnic University, Hong Kong SAR, China; 45 Population Health Sciences, Bristol Medical School, University of Bristol, Bristol, BS8 1NU UK; 46 Department of Ophthalmology, Medical Faculty Mannheim, Heidelberg University, Mannheim, Germany; 47 State Key Laboratory of Ophthalmology, Zhongshan Ophthalmic Center, Sun Yat-Sen University, Guangzhou, China; 48 Centre for Eye Research Australia, Royal Victorian Eye and Ear Hospital, University of Melbourne, Melbourne, VIC Australia; 49 Department Ophthalmology, Radboud University Medical Center, Nijmegen, the Netherlands; 50 Computational and Statistical Genomics Branch, National Human Genome Research Institute, National Institutes of Health, Bethesda, MD USA; 51 Duke-NUS Medical School, Singapore, Singapore; 52 Department of Ophthalmology, National University Health Systems, National University of, Singapore, Singapore; 53 Department of Ophthalmology, Menzies Institute of Medical Research, University of Tasmania, Hobart, Australia; 54 Centre for Global Health Research, Usher Institute for Population Health Sciences and Informatics, University of Edinburgh, Edinburgh, UK; 55 Department of Ophthalmology and Visual Sciences, The Chinese University of Hong Kong, Hong Kong Eye Hospital, Kowloon, Hong Kong China; 56 Division of Epidemiology and Clinical Applications, National Eye Institute/National Institutes of Health, Bethesda, USA; 57 Department of Ophthalmology, Flinders University, Adelaide, Australia; 58 Great Ormond Street Institute of Child Health, University College London, London, UK; 59 Department of Ophthalmology and Visual Sciences, John Moran Eye Center, University of Utah, Salt Lake City, Utah USA; 60 Université de Bordeaux, Inserm, Bordeaux Population Health Research Center, team LEHA, UMR 1219, F-33000 Bordeaux, France; 61 Translational Research Institute, University of Queensland Diamantina Institute, Brisbane, Queensland Australia; 62 MRC Integrative Epidemiology Unit, University of Bristol, Bristol, UK; 63 Department of Population Health Sciences, Bristol Medical School, Bristol, UK; 64 Statistical Genetics, QIMR Berghofer Medical Research Institute, Brisbane, Australia; 65 University of Leeds, Leeds, UK; 66 NIHR Biomedical Research Centre, London, UK; 67 University of Southampton, Southampton, UK; 68 Cardiff University, Cardiff, UK; 69 Department of Ophthalmology, Columbia University, New York, USA; 70 Department of Population and Quantitative Health Sciences, Case Western Reserve University, Cleveland, Ohio USA; 71 Department of Ophthalmology and Visual Sciences, Case Western Reserve University and University Hospitals Eye Institute, Cleveland, Ohio USA; 72 Department of Genetics, Case Western Reserve University, Cleveland, Ohio USA; 73 Department of Clinical Physiology, Tampere University Hospital and School of Medicine, University of Tampere, Tampere, Finland; 74 Finnish Cardiovascular Research Center, Faculty of Medicine and Life Sciences, University of Tampere, Tampere, Finland; 75 Gloucestershire Hospitals NHS Foundation Trust, Gloucestershire, UK; 76 Institute for Molecular Medicine Finland FIMM, HiLIFE Unit, University of Helsinki, Helsinki, Finland; 77 Department of Ophthalmology and Visual Sciences, University of Wisconsin–Madison, Madison, Wisconsin USA; 78 Department of Clinical Chemistry, Finnish Cardiovascular Research Center-Tampere, Faculty of Medicine and Life Sciences, University of Tampere, Tampere, Finland; 79 Department of Clinical Chemistry, Fimlab Laboratories, University of Tampere, Tampere, Finland; 80 National Human Genome Research Institute, National Institutes of Health, Baltimore, USA; 81 Nottingham University Hospitals NHS Trust, Nottingham, UK; 82 Department of Ophthalmology, Yokohama City University School of Medicine, Yokohama, Kanagawa Japan; 83 Department of Ophthalmology, University Medical Center of the Johannes Gutenberg-University Mainz, Mainz, Germany; 84 Institute of Neurogenomics, Helmholtz Zentrum München, German Research Centre for Environmental Health, Neuherberg, Germany; 85 Program in Genetics and Genome Biology, Hospital for Sick Children and University of Toronto, Toronto, Ontario Canada; 86 NIHR Biomedical Research Centre, Moorfields Eye Hospital NHS Foundation Trust and UCL Institute of Ophthalmology, London, UK; 87 Ulverscroft Vision Research Group, University College London, London, UK; 88 Research Centre of Applied and Preventive Cardiovascular Medicine, University of Turku, Turku, Finland; 89 Department of Clinical Physiology and Nuclear Medicine, Turku University Hospital, Turku, Finland; 90 Department of Ophthalmology, University of Pennsylvania, Philadelphia, Pennsylvania USA; 91 Department of Genetics, Genomics and Informatics, University of Tennessee Health Sciences Center, Memphis, Tenessee USA; 92 Beijing Tongren Eye Center, Beijing Key Laboratory of Intraocular Tumor Diagnosis and Treatment, Beijing Ophthalmology & Visual Sciences Key Lab, Beijing Tongren Hospital, Capital Medical University, Beijing, China; 93 MRC Human Genetics Unit, MRC Institute of Genetics & Molecular Medicine, University of Edinburgh, Edinburgh, UK; 94 Department of Epidemiology and Medicine, Johns Hopkins Bloomberg School of Public Health, Baltimore, Maryland USA; 95 Wilmer Eye Institute, Johns Hopkins Medical Institutions, Baltimore, Maryland USA; 96 Department of Ophthalmology, Otsu Red Cross Hospital, Nagara, Japan; 97 School of Ophthalmology and Optometry, Eye Hospital, Wenzhou Medical University, Wenzhou, China; 98 University of Manchester, Manchester, England; 99 Kingston University, London, USA; 100 King’s College London, London, USA; 101 Queens University Belfast, Belfast, UK; 102 University College London, London, USA; 103 University of Edinburgh, Edinburgh, UK; 104 University of Bristol, Belfast, UK; 105 University of Oxford, Oxford, USA; 106 University of Liverpool, Liverpool, UK; 107 University of Cambridge, Cambridge, USA; 108 Leeds Teaching Hospitals NHS Trust, Leeds, UK; 109 King’s College Hospital NHS Foundation Trust, London, UK; 110 University of London, London, UK; 111 Newcastle University, Newcastle, UK; 112 University of Dundee, Dundee, UK; 113 University of East Anglia, Anglia, UK

**Keywords:** Genome-wide association studies, Genetic predisposition to disease, Corneal diseases

## Abstract

Corneal curvature, a highly heritable trait, is a key clinical endophenotype for myopia - a major cause of visual impairment and blindness in the world. Here we present a trans-ethnic meta-analysis of corneal curvature GWAS in 44,042 individuals of Caucasian and Asian with replication in 88,218 UK Biobank data. We identified 47 loci (of which 26 are novel), with population-specific signals as well as shared signals across ethnicities. Some identified variants showed precise scaling in corneal curvature and eye elongation (i.e. axial length) to maintain eyes in emmetropia (i.e. *HDAC11*/*FBLN2* rs2630445, *RBP3* rs11204213); others exhibited association with myopia with little pleiotropic effects on eye elongation. Implicated genes are involved in extracellular matrix organization, developmental process for body and eye, connective tissue cartilage and glycosylation protein activities. Our study provides insights into population-specific novel genes for corneal curvature, and their pleiotropic effect in regulating eye size or conferring susceptibility to myopia.

## Abstract (web summary)

Qiao Fan et al. report a genome-wide association analysis of corneal curvature—an important measurement for identifying vision problems, such as myopia—in more than 45,000 individuals of either European or Asian ancestry. They identify 47 loci, 26 of which are novel, with some showing population-specific effects and pleiotropic effects on eye elongation and myopia.

## Introduction

Refractive error is common worldwide and particularly so in Asia, where uncorrected refractive error is one of the major causes of visual impairment and blindness^1,2^. In 2015, uncorrected refractive error caused moderate or severe visual impairment in 116 million people, and blindness in 7.4 million people—these figures are expected to rise to 128 million and 8.0 million, respectively, by 2020^3^. Thus there is a critical need to better understand the genetic basis of how different optical components may contribute to ammetropia, for which corneal curvature represents a main endophenotype.

Corneal curvature (CC) is a key clinical endophenotype for the refractive status of the eye. The corneal air-tissue interface provides approximately two-thirds of the eye’s optical power^4^. Thus, changes in the CC significantly affect refractive error, such as myopia. A steeper CC was associated with a more negative/myopic refractive error. In the emmetropic eye, the refractive power of the eye’s optical components (such as CC) must be appropriate to its axial length (AL). If the changes in CC, AL, or other ocular components such as lens thickness or anterior chamber depth are not aligned, refractive errors (myopia or hyperopia) are likely to occur.

Clinically, CC associates with ethnicity^5,6^, age^7^, and anthropometric features (height and weight)^7^. Based on family and twin studies, CC is highly heritable, with 35–95% of inter-individual CC variation attributed to genetic factors^8–12^. Previous genome-wide association studies (GWASs) have been successful in identifying more than 160 loci associated with refractive error^13–15^, and nine loci associated with AL^16^. Only four loci associated with CC have been previously reported from GWAS analyses: *MTOR*^17^*, CMPK1*^18^, and *RBP3*^18^ identified in Asians, and *PDGFRA* in both Asians^17^ and Europeans^11,19^. A further 31 loci in European emmetropes, accounting for an additional 2.3% of variance in CC, have also been provisionally reported^20^. Associated variants identified to date cannot fully explain the additive genetic variance of CC, and hence other genetic variants are likely to contribute to this endophenotype. The difference in the prevalence of myopia in various ethnic groups, particularly in Asia, also suggests that certain CC-associated alleles may be population-specific^11^. Furthermore, the extent to which shared or distinct genetic loci contribute to variation in CC, AL, and spherical equivalent is uncertain.

Thus, we conducted the largest GWAS meta-analysis of CC to date, incorporating both European and Asian cohorts in a single analysis from the Consortium for Refractive Error and Myopia (CREAM) and validated our findings in the United Kingdom (UK) Biobank.

## Results

### Primary GWAS of corneal curvature

The CREAM discovery cohorts included 29,580 individuals with European ancestry from 18 studies, and 14,462 individuals with Asian ancestry from 10 studies. The demographics of these 44,042 participants are shown in Supplementary Table 1. GWAS analyses for CC were performed at the cohort level for all variants genotyped or imputed using the 1000 Genomes Project data as reference panels^7^ (Supplementary Table 2). The genomic control inflation factor (λ_GC_: 0.872–1.085**)** showed little evidence of inflation in test statistics at the study level.

We applied a uniform set of quality-control procedures to all cohort-level GWAS results in CREAM and meta-analysed up to 8.94 million variants (see Methods). For the discovery phase, we performed an inverse-variance-weighted meta-analysis on the European and Asian populations. The quantile-quantile plot for the trans-ethnic meta-analysis (λ_GC_: 1.119; Supplementary Fig. 1) indicated moderate inflation, and genomic control-adjusted test statistics were generated. The inflation is partially due to polygenicity as the linkage disequilibrium (LD)-score regression intercepts were close to one^21^ (LD-score regression intercept of 1.045 in Europeans and 1.013 in Asians).

**Fig. 1:**
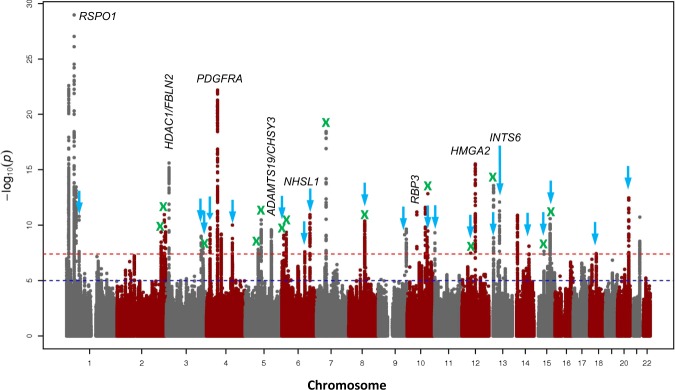
Manhattan plot of trans-ethnic GWAS meta-analysis for corneal curvature. Both directly genotyped and imputed variants were meta-analysed for corneal curvature in 44,042 CREAM participants. The *y*-axis represents −log_10_*p* values for association with corneal curvature, and the *x*-axis represents genomic position based on human genome build 37, highlighting newly identified loci (arrows in blue; Table 1), loci associated with axial length (labelled with nearest gene names), and loci associated with spherical equivalent (cross in green). The horizontal red line indicates the genome-wide significance level of *P* < 5.0 × 10^−8^. The horizontal blue line indicates the suggestive significance level of *P* < 1.0 × 10^−5^.

Figure 1 shows the Manhattan plot for the trans-ethnic meta-analysis for CC. We identified 41 loci at genome-wide significance (*P* *<* 5.0 × 10^−8^; Table 1; Supplementary Figs. 2–4). We performed replication analyses of these loci for CC in 88,218 participants with European ancestry from the UK Biobank. Thirty-seven (90.2%) of the 41 lead variants passed genome-wide significance in the UK Biobank data (Table 1). The signals of the two loci (*CMPK1/STIL, RBP3*) not reaching genome-wide significance in the replication phase were mainly driven by the CREAM Asian populations. In the combined CREAM and UK Biobank meta-analysis, the five most strongly associated loci were *RSPO1* (rs4074961; *P* = 6.51 × 10^−100^), *HUS1* (rs12702376; *P* *=* 2.77 × 10^−86^), *FGF9* (rs9506725; *P* = 1.21 × 10^−78^), *PDGFRA* (rs1800813; *P* = 1.59 × 10^−73^), and *HDAC11/FBLN2* (rs2630445; *P* = 4.92 × 10^−67^). The *RSPO1* gene was previously identified as the strongest locus with the same lead SNP rs4074961 associated with AL in CREAM^16^. Here it stands out as the most significant locus for CC in our large meta-analysis. We confirmed associations with CC in the four previously identified loci in our samples: *PDGFRA, MTOR, RBP3*, and *CMPK1*^11,17–19^, and in an additional 17 loci provisionally identified in the European emmetropes from the UK Biobank^20^. In total, we identified 20 novel CC loci through single-variant analysis.

**Table 1:** Summary of SNPs associated with corneal curvature in CREAM populations (*P* < 5.0 × 10^−8^), with replication in UK Biobank data.

					CREAM-eur (*n* = 29,580)			CREAM-asn (*n* = 14,462)			CREAM (*n* = 44,042)				UK Biobank (n=88,218)			All combined	
SNP	CHR	POS	GENE(S)	A1/A2	EAF	β	*P*	EAF	β	*P*	β	*P*	*Het-I*^2^	*het-P*	Freq	β	*P*	*Het-I*^2^	*P*
rs3737611	1	11186897	*MTOR*	A/G	0.98	−0.040	2.08E-05	0.83	−0.041	6.73E-21	−0.041	2.50E-23	0.00	0.523	0.98	−0.041	1.30E-23	0.00	1.36E-46
rs4074961	1	38092723	*RSPO1*	T/C	0.43	0.024	5.83E-22	0.46	0.019	4.10E-10	0.022	1.06E-29	16.20	0.206	0.44	0.022	1.40E-73	0.00	6.51E-100
rs60078183^a^	1	47857307	*CMPK1/STIL*	A/G	0.00	NA	NA	0.21	−0.037	3.82E-15	−0.037	3.59E-14	0.00	0.694	0.00	−0.034	4.80E-01	0.00	4.47E-16
**rs945170**	1	62867483	*USP1*	T/G	0.27	−0.018	5.11E-12	0.33	−0.007	2.91E-02	−0.014	1.61E-11	27.74	0.067	0.26	−0.012	1.10E-19	0.00	2.64E-30
rs1548942	2	217619036	*IGFBP5/TNP1*	T/C	0.15	0.016	5.62E-07	0.19	0.016	1.05E-04	0.016	4.23E-10	0.00	0.757	0.15	0.021	6.40E-37	3.76	5.14E-50
rs2245601	2	233390937	*CHRND/RPSS56*	A/G	0.49	−0.014	5.27E-09	0.38	−0.013	2.95E-04	−0.014	1.10E-11	0.00	0.641	0.52	−0.015	5.40E-38	0.00	1.29E-48
rs2630445^a^	3	13554886	*HDAC11/FBLN1*	T/G	0.10	0.030	1.17E-15	0.00	NA	NA	0.030	1.17E-15	0.00	0.578	0.10	0.031	8.80E-55	0.00	4.92E-67
**rs485554**	3	172137343	*FNDC3B/GHSR*	C/G	0.31	−0.015	1.78E-08	0.49	−0.009	5.08E-03	−0.012	1.01E-09	25.11	0.094	0.32	−0.012	1.60E-19	0.00	2.12E-27
**rs10663094**	3	181363464	*SOX2*	ACT/A	0.34	0.011	1.48E-05	0.41	0.013	8.88E-05	0.012	9.82E-09	1.94	0.435	0.35	0.012	9.70E-23	0.00	1.27E-30
**rs16896276**	4	18015156	*LCORL*	A/T	0.27	0.014	1.28E-07	0.38	0.014	2.02E-04	0.014	1.74E-10	0.00	0.951	0.26	0.013	5.70E-21	0.00	1.19E-31
rs1800813	4	55094467	*PDGFRA*	A/G	0.21	−0.022	9.90E-15	0.23	−0.024	2.19E-10	−0.023	6.44E-23	36.64	0.019	0.22	−0.020	5.80E-45	0.41	1.59E-73
rs7657200	4	73473151	*ADAMTS3*	A/G	0.38	0.016	1.48E-09	0.37	0.008	2.20E-02	0.013	6.50E-10	0.00	0.873	0.39	0.014	3.70E-29	0.00	2.23E-38
**rs6831679**	4	128113424	*RP11-125O18.1*	A/G	0.27	0.013	2.80E-07	0.40	0.013	4.42E-05	0.013	9.78E-11	0.00	0.808	0.28	0.008	3.50E-10	3.57	1.14E-18
rs11740254	5	64319640	*CWC27/ADAMTS6*	T/C	0.40	0.014	2.48E-07	0.27	0.010	1.15E-02	0.013	1.55E-08	0.00	0.827	0.46	0.011	6.70E-21	0.00	4.96E-30
rs13180294	5	79360175	*THBS4*	A/G	0.08	−0.028	1.17E-10	0.05	−0.018	6.68E-02	−0.027	3.37E-11	0.00	0.479	0.10	−0.015	7.50E-14	6.85	2.46E-22
rs7708378^a^	5	129088784	*ADAMTS19/CHSY3*	T/G	0.07	0.028	2.49E-09	0.00	NA	NA	0.028	2.49E-09	0.00	0.474	0.08	0.028	2.80E-34	0.00	5.51E-45
**rs67612840**	6	10028826	*OFCC1*	A/T	0.28	−0.012	3.34E-06	0.26	−0.015	2.42E-05	−0.013	8.26E-10	4.24	0.398	0.27	−0.014	6.40E-26	0.00	4.11E-35
rs9366426	6	22064639	*CASC15*	T/C	0.44	0.014	2.11E-08	0.78	0.012	4.00E-03	0.013	4.42E-10	26.55	0.081	0.43	0.013	9.30E-28	0.00	5.39E-37
**rs961755**	6	113443139	*U6*	T/G	0.20	0.011	1.91E-04	0.33	0.014	1.32E-05	0.013	2.49E-08	0.00	0.844	0.18	0.014	2.30E-21	0.00	1.49E-30
**rs4620141**	6	138869568	*NHSL1*	T/C	0.36	−0.013	2.90E-07	0.58	−0.015	3.35E-06	−0.014	1.12E-11	19.17	0.164	0.36	−0.008	5.10E-11	5.93	1.74E-20
rs12702376	7	47775053	*HUS1*	C/G	0.84	−0.028	9.25E-18	0.86	−0.024	9.69E-03	−0.028	3.52E-19	12.45	0.286	0.85	−0.029	2.40E-67	0.00	2.77E-86
**rs7004112**	8	78941331	*RP11-91P17.1*	T/G	0.40	−0.010	3.08E-05	0.50	−0.017	1.82E-08	−0.013	4.20E-11	6.85	0.353	0.37	−0.014	1.10E-28	0.00	6.39E-38
**rs4837104**	9	129433929	*LMX1B*	A/G	0.05	0.024	1.43E-04	0.27	0.021	4.29E-07	0.022	7.35E-10	0.00	0.956	0.04	0.022	1.90E-12	0.00	2.73E-24
rs3132309	9	137433436	*COL5A1*	A/C	0.42	−0.015	3.52E-08	0.44	−0.011	7.98E-04	−0.014	2.27E-10	0.00	0.550	0.41	−0.013	3.10E-24	0.00	7.00E-35
rs11204213^a^	10	48388228	*RBP3*	T/C	0.00	NA	NA	0.04	0.071	9.83E-13	0.071	9.83E-13	0.00	0.741	0.00	0.063	1.50E-01	0.00	4.36E-13
rs166976	10	90024599	*RNLS*	A/G	0.72	−0.018	1.26E-10	0.80	−0.012	1.45E-03	−0.016	2.47E-12	5.52	0.378	0.73	−0.015	3.80E-30	0.00	4.17E-44
**rs10786330**	10	99074959	*FRAT1/FRAT2/ARHGAP19*	A/C	0.58	0.012	2.47E-06	0.65	0.013	6.11E-05	0.012	1.22E-09	0.00	0.980	0.60	0.008	4.00E-12	1.93	1.54E-19
rs807037	10	102824349	*KAZALD1*	C/G	0.67	−0.014	4.33E-08	0.49	−0.017	2.02E-07	−0.015	1.43E-13	0.00	0.631	0.66	−0.017	9.10E-42	0.00	7.12E-54
**rs7948458**	11	2172830	*IGF2*	A/C	0.20	−0.014	9.53E-06	0.51	−0.015	5.33E-06	−0.014	4.90E-10	40.44	0.008	0.19	−0.016	6.50E-26	0.00	1.66E-36
**rs11181913**	12	43574200	*ADAMTS20*	A/G	0.90	0.020	2.02E-06	0.92	0.018	3.50E-03	0.019	3.30E-08	0.00	0.645	0.91	0.016	4.40E-14	0.00	1.03E-22
rs7959830	12	66347368	*HMGA2*	T/G	0.42	−0.020	2.53E-16	0.80	−0.009	5.66E-02	−0.018	3.02E-16	34.50	0.027	0.42	−0.018	3.30E-17	5.41	9.35E-47
rs9506725^a^	13	22314146	*FGF9*	T/C	0.64	0.019	3.07E-14	1.00	NA	NA	0.019	3.07E-14	0.33	0.456	0.63	0.020	5.10E-59	0.00	1.21E-78
**rs9316971**	13	22980191	*SNORD36*	T/C	0.28	−0.015	2.43E-07	0.48	−0.010	1.63E-03	−0.013	3.21E-09	32.34	0.044	0.29	−0.009	2.20E-12	1.91	3.51E-21
**rs7327381**	13	52006645	*INTS6*	T/C	0.40	0.015	2.16E-09	0.53	0.013	4.27E-05	0.014	8.06E-13	0.00	0.829	0.41	0.012	1.50E-23	0.00	3.74E-34
rs75772222	14	25447080	*STXBP6*	T/C	0.11	0.021	3.21E-08	0.07	0.024	4.55E-05	0.021	1.28E-11	0.00	0.983	0.11	0.020	5.00E-27	0.00	9.79E-38
**rs4083463**	14	81856323	*STON2*	A/G	0.31	0.012	1.91E-06	0.34	0.011	7.14E-04	0.012	7.92E-09	4.26	0.397	0.33	0.010	2.00E-16	0.00	3.12E-24
**rs9806595**	15	48755168	*FBN1*	T/C	0.77	−0.009	1.68E-03	0.67	−0.018	1.49E-07	−0.012	2.23E-08	0.00	0.691	0.77	−0.004	4.20E-03	10.40	6.43E-09
**rs4887113**	15	79095287	*ADAMTS7*	T/C	0.40	0.016	3.08E-09	0.18	0.021	1.21E-03	0.016	2.57E-11	0.00	0.798	0.45	0.012	1.60E-24	2.11	5.74E-38
**rs184926585**	18	32643292	*MAPRE2*	T/C	0.93	−0.020	2.19E-05	0.82	−0.021	2.63E-04	−0.020	3.61E-08	0.00	0.756	0.92	−0.007	1.30E-03	11.53	4.86E-11
**rs6064518**	20	55821046	*BMP7*	T/G	0.33	0.017	4.85E-11	0.16	0.021	1.11E-03	0.017	3.47E-13	0.00	0.809	0.32	0.013	1.20E-24	2.15	6.73E-40
rs13050142	21	47427165	*COL6A1*	T/C	0.29	−0.016	1.71E-08	0.22	−0.022	9.43E-05	−0.017	1.86E-11	0.00	0.964	0.31	−0.013	4.80E-25	1.87	2.45E-40

Lead variants shown were genome-wide significant (*P* < 5.0E-8) in subjects of CREAM of European and Asian ancestry (Stage 1), with results of replication in UK Biobank data (Stage 2). Variants in bold indicate new loci.

*SNP* single-nucleotide polymorphism, *Chr* chromosome; Nearest gene in 200 kb flanking the lead SNP based on NCBI build 37; *A1* effect allele, *A2* reference allele, *EAF* effect allele frequency, *β* effect size of corneal curvature in millimeter based on the effect allele A1; *Het-I*^*2*^: heterogeneous effects *I*^*2*^ (%) between the Studies.

^a^Lead SNPs identified were monomorphic/extremely rare in Europeans or Asian populations.

Following the age classification scheme adopted by the CREAM consortium, we stratified the CREAM samples into younger (age < 25 years) and older (age ≥ 25 years) groups. In the older group (*n* = 35,442), the top five genome-wide significant loci were *RSPO1, MTOR, PDGFRA1, HUS1*, and *FGF9* (Supplementary Data 1). There were no novel genome-wide significant hits for CC in both groups. No variants reached genome-wide significance in the younger group (*n* = 8620), likely due to the insufficient power in contrast to the older group. The effect sizes and directions of effect for the top variants were consistent between the younger and older groups.

### Trans-ethnic comparison of genotypic effects in Europeans versus Asians

**Fig. 2:**
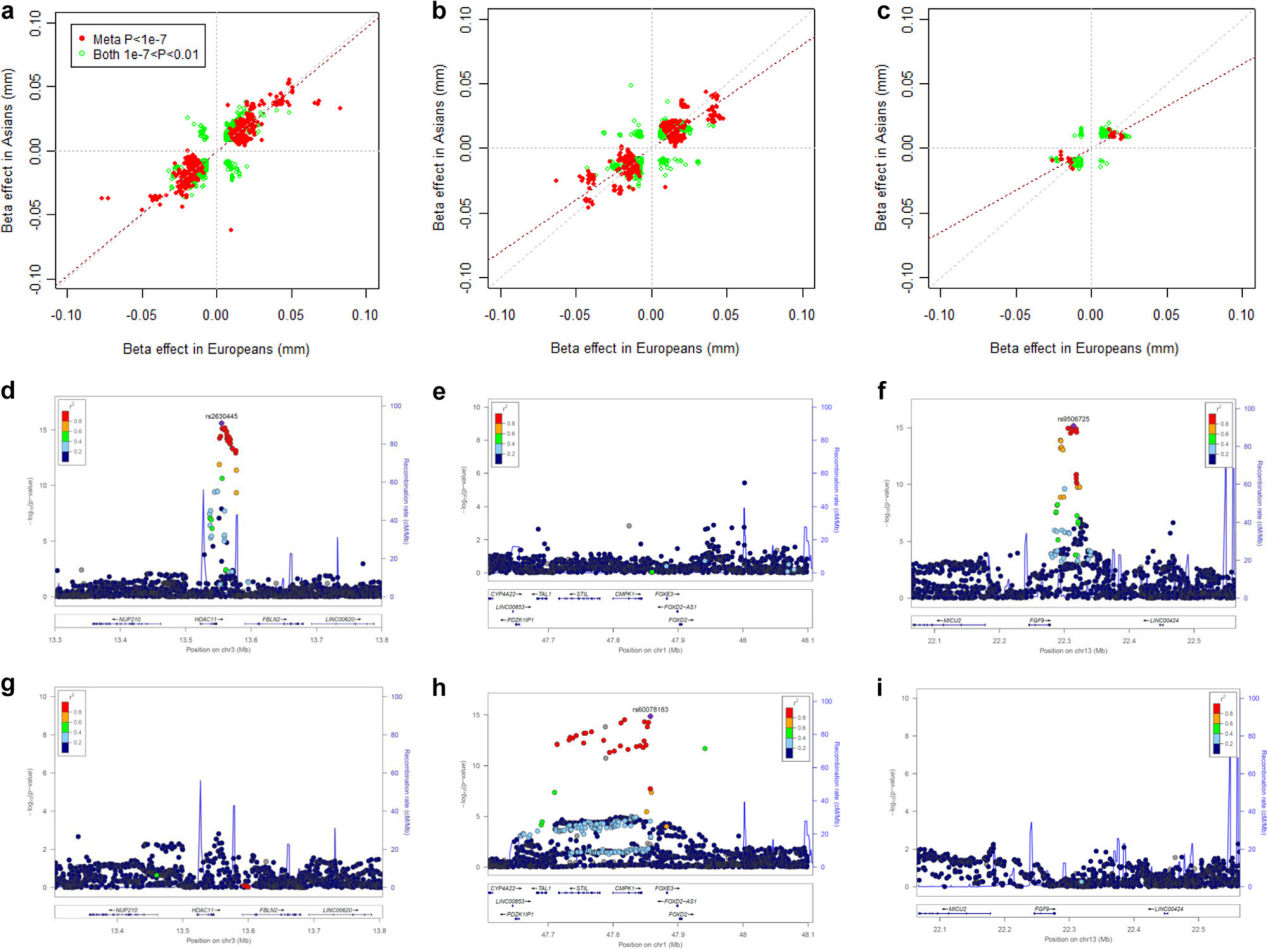
Concordance of effect sizes of variants between European and Asian populations and loci showing population-specific signals. **a**–**c** For each scatter plot, effect size in Asians (*x*-axis) and in Europeans (*y*-axis) was plotted for variants with *P* *<* 0.01 in both ancestry groups in CREAM. The variants were grouped based on the allele frequency difference between European and Asian populations: **a** <0.1; **b** 0.1–0.3, and **d** >0.3. The red dot represents variants with *P* < 1.0 × 10^−7^ in the meta-analysis of combined population, and green circle indicates variant with 1.0 × 10^−7^ < *P <* 0.01 in both Europeans and Asians. Dashed line in red is the fitted line and in grey is the x = y line of unity. **d**–**i** Regional plots in CREAM Europeans (**d**–**f**) and Asians (**g**–**i**) showing population-specific signals at loci exhibiting allele frequency differences: *HDAC11/FBLN2* (**d**, **g**), *CMPK1/STIL* (**e**, **h**) and **c** *FGF9* (**f**, **i**). Here we present regional plots for three lead variants. (i) Lead variant rs2630445 in plot **d** showing genome-wide association signals in Europeans (MAF = 0.10) is monomorphic in Asian populations. (ii) Lead variant rs60078183 in plot **h** exhibiting association in Asians (MAF = 0.21) is monomorphic in European populations. (iii) Lead variant rs9506725 in plot **f** showing association in Europeans (MAF = 0.36) is monomorphic in Asian populations.

Among the 41 lead variants identified in the full discovery samples, the effect size and direction of effect were largely consistent across Europeans and Asians (Table 1). To evaluate whether genetic effect sizes were consistent in Europeans versus Asians in the CREAM samples, we compared additive effect sizes (beta coefficient in millimetre per allele) of variants with *p*-value < 0.01 in both populations. We grouped these variants by inter-population allele frequency discrepancy between the two ancestry groups (<0.1, 0.1–0.3, and >0.3). Overall, the variants were concordant in direction of effects and effect sizes (Fig. 2a–c). The effect sizes appeared most consistent in variants with little discrepancy in allele frequency (2**a**; allele frequency difference < 0.1), and less consistent in variants of larger allele frequency discrepancy (**2c;** allele frequency difference > 0.3).

The high concordance of genetic effects between Europeans and Asians, however, could not rule out the possibility of discrepancy at some loci with large inter-population differences in the allele frequency and LD structure. Three loci in our all-ancestry analyses were driven by European populations (*HDAC11/FBLN2* rs2630445*, ADAMTS19/CASY3* rs7708378, and *FGF9* rs9506725), and two loci were driven by Asian populations (*RBP3* rs11204213 and *CMPK1/STIL* rs60078183). In the ethnic group (either the Asian or European populations) where these variants were not associated, they typically presented as monomorphic in the other ethnic group, as well as those variants in LD with the lead variant (*r*^2^ ≥ 0.8; Supplementary Data 2). Regional plots at *HDAC11/FBLN2* rs2630445, *CMPK1/STIL* rs60078183, and *FGF9* rs9506725 were presented in Europeans (Fig. 2d–f) and Asians (Fig. 2g–i), respectively, with inter-population allele frequency at 0.10, 0.20, and 0.36. The proxy SNPs adjacent showed minimal significance at only two loci in Asians (*HDAC11/FBLN2 rs2655225*, *P* = 3.34 × 10^−3^, *r*^*2*^ = 0.62) and *ADAMTS19/CASY3 rs11746536*; *P* = 2.67 × 10^−4^, *r*^*2*^ = 0.11; Supplementary Table 3). For GWAS analyses performed separately in Europeans and Asians, there were two Asian-specific loci (*EMX2/EMX2OS* rs2240776, *P* = 2.45 × 10^−8^; *NCAPG* rs7672919, *P* = 3.90 × 10^−8^; Supplementary Table 4); both did not reach genome-wide significance in the combined analysis. In addition, one locus showing suggestive significance in CREAM Europeans (*PIEZO2* rs2101976; *P* = 7.32 × 10^−8^) has been replicated in the UK Biobank data (*P* = 8.90 × 10^−14^).

We calculated SNP-heritability (SNP-*h*^2^) using GWAS summary statistics^22^. The SNP-*h*^2^ estimate for CC in Asians (0.196, *s.e*. = 0.036) was numerically lower than in Europeans (0.267, *s.e*. = 0.024), but there was no statistical evidence for a meaningful difference. A similar pattern was noted in a previous study^12^.

### Association of corneal curvature loci with spherical equivalent and axial length

**Fig. 3:**
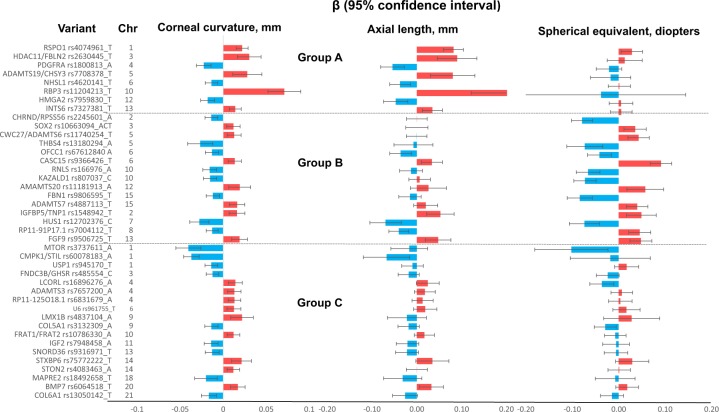
Effect sizes on cornea curvature, axial length and spherical equivalent for CC-associated variants. Corneal curvature (CC)-associated genetic variants identified from CREAM (*n* = 44,042) were grouped based on the patterns of the associations of effect alleles with axial length (AL; *n* = 10,851) and spherical equivalent (*n* = 95,505). Group A—variants associated with AL only (‘eye-size’ determining genetic variants); the effect allele of each variant was associated with eye size: a larger eye with both a flatter CC and longer AL (positive β on both CC and AL; bar in red), and a smaller eye with both a steeper CC and shorter AL (negative β; bar in blue). These variants were not associated with spherical equivalent. Group B—variants associated with spherical equivalent; the allele associated with a steeper CC was associated with a more negative refractive error (or vice versa). These variants were not associated with AL, except those at loci *IGFBP5/TNP1, HUS1, RP11-91P17.1*, and *FGF9*. Group C—variants not associated with spherical equivalent or AL. For the associations with axial length and spherical equivalent, *FDR* < 0.01 was considered significance. The colour of the bar represents a positive genetic effect (in red) or a negative genetic effect (in blue).

In further analyses, we assessed the associations of the 41 CC lead variants with spherical equivalent^14,15^ in 95,505 participants from the UK Biobank, as well as in a subset of CREAM participants with AL measurement (*N* = 10,851; Supplementary Table 5). The lead variants were categorized into the following three groups using false discovery rates (FDR) set at a threshold of 1% from the Benjamini-Hochberg procedure^23^ (Fig. 3 and Supplementary Data 3).

**Fig. 4:**
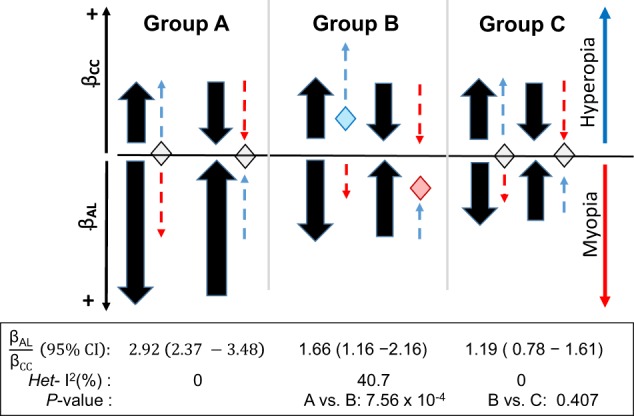
Illustration of pleiotropic effect ratio $\frac{{\upbeta}_{\mathrm{AL}}}{{\upbeta}_{\mathrm{CC}}}$ and effects toward emmetropic and myopic states. The figure illustrates genetic effects of AL (β_AL_) might or might not compensate genetic effects of corneal curvature (β_CC_) toward myopia or hyperopia. Longer CC (shown by positive β_CC_; arrow upward) tends to make the eye hyperopic (dashed line in blue) and longer AL (positive β_CC_; arrow downward) tends to make the eye more myopic (dashed line in red). Similarly, steeper CC (negative β_CC_, arrow downward) tends to make the eye more myopic and shorter AL (negative β_CC_; arrow downward) tends to make the eye less myopic. The compensatory pleiotropic effects β_AL_ could offset β_CC_ on myopia or hyperopia at the pleiotropic ratio $\frac{{\upbeta}_{{\mathrm{AL}}}}{{\upbeta}_{{\mathrm{CC}}}}$ ~ 3, as shown in group A. The compensatory pleiotropic effects β_AL_, however, cannot offset β_CC_ on myopia or hyperopia at smaller pleiotropic ratio $\frac{{\upbeta}_{{\mathrm{AL}}}}{{\upbeta}_{{\mathrm{CC}}}}$, as shown in group B. There might be other pleiotropic effect in Group C, besides AL, to compensate genetic effect of CC on myopia. *Het-I*^2^, for heterogeneous effects between the variants. All P-value for heterogeneity was >0.05. Pleiotropic effect ratio was calculated at each variant and combined to estimate $\frac{{\upbeta}_{{\mathrm{AL}}}}{{\upbeta}_{{\mathrm{CC}}}}$ and heterogeneity using the meta-analysis approach (see Methods). Grouping of A, B, and C was the same as in Fig. 3.

Eight CC variants were associated with AL, but not spherical equivalent (Group A, Fig. 3). That is, the effect allele of the variant was associated with eye size, e.g. a larger eye with both a flatter CC and longer AL (positive genetic effects on both CC and AL; bar in red, Fig. 3) or a smaller eye with both a steeper CC and shorter AL (negative genetic effects; bar in blue), but was not associated with spherical equivalent. For instance, *HMGA2* rs7959830 T allele was associated with a steeper CC (*β* *=* −0.018, *s.e*. = 0.002, *P* = 3.02 × 10^−16^) and shorter AL (*β* *=* −0.047, *s.e*. = 0.014, *P* = 6.54 × 10^−4^), but not spherical equivalent (*β* = 0.005, *s.e*. = 0.013, *P* = 0.670). A lack of association with refractive error at these variants might be explained on the basis of the compensatory effects resulting in, a steeper CC (that tends to make the eye more myopic) and shorter AL (that tends to make the eye more hyperopic/less myopic), or a flatter CC and longer AL The compensatory genetic effects for CC and AL relevant to the effects on myopia or hyperopia is illustrated in Fig. 4. Among these variants, the pleiotropic association on AL of *HDAC11/FBLN2* rs2630445, *ADAMTS19/CHSY* rs7708378 were mainly driven from European populations, and *RBP3* rs11204213 from Asian populations.

Fifteen CC variants were associated with spherical equivalent (Group B, Fig. 3). Eleven of these variants were not associated with AL. The strongest signals associated with spherical equivalent (*P* *<* 1 × 10^−7^) were *CASC15* rs9366426, *CHRND/PRSS56* rs2245601, *KAZALD1* rs807037, *FBN1* rs9806595, and *RNLS* rs166976. Among these 11 variants, the allele associated with a steeper CC was associated with a more negative/myopic refractive error (or a flatter CC and more positive/hyperopic refractive error). For instance, *FBN1* rs9806595 T allele was associated with a steeper CC (*β* *=* −0.012, *s.e*. = 0.002, *P* = 2.23 × 10^−8^) and more myopic refractive error (*β* = −0.084, *s.e*. = 0.014, *P* = 1.40 × 10^−8^), but not AL (*β* = −0.015, *s.e* = 0.013, *P* = 0.231). For the remaining CC variants at four loci that were associated spherical equivalent as well as AL (*HUS1*, *RP11-91P17.1, FGF9*, and *IGFBP5/TNP1)*, the direction of genetic effect on spherical equivalent may depend on the relative magnitude of effect on CC versus AL. For instance, although both *HUS1* rs12702376 C allele and *RP11-91P17.1* rs7004112 T allele were associated with steeper CC (that tends to make the eye more myopic) and shorter AL (that tends to make the eye less myopic), *HUS1* was associated with a negative/myopic refractive error, while *RP11-91P17.1* was associated with a positive/hyperopic refractive error. Among these variants, pleiotropic effects of *FGF9* rs9506725 were mainly driven from European populations.

The remaining 18 CC variants (Group C, Fig. 3) were not associated with spherical equivalent or AL at FDR > 1%. The association on CC and spherical equivalent at the majority loci was, although not significant, in an expected direction, e.g. with a steeper CC and a more negative/myopic refractive error (or vice versa). Among these variants, *CMPK1/STIL* rs60078183 was mainly driven by Asian populations. The pleiotropic genetic effects on spherical equivalent of CC-associated variants, together with previously implicated AL variants^16,24^, are summarized in Supplementary Fig. 5.

The pleiotropic effect ratio $\frac{{{\upbeta}_{\mathrm{AL}}}}{{{\upbeta}_{\mathrm{CC}}}}$ was meta-analyzed across all variants in each group (Fig. 4; Supplementary Table 6). The pleiotropic ratio $\frac{{{\upbeta}_{\mathrm{AL}}}}{{{\upbeta}_{\mathrm{CC}}}}$ in group A (2.92, 95% CI: 2.37–3.48) was larger than that in group B (1.66, 95% CI: 1.16–2.16) and C (1.19, 95% CI: 0.78–1.61), with *p*-value at 7.56 × 10^−4^ and 1.68 × 10^−6^, respectively. For variants in group A, the pleiotropic effect for AL could offset genetic effect for CC towards myopia or hyperopia; namely, a genetically determined 1 mm increase (or decrease) for CC accompanied by a 2.92 mm increase (or decrease) on average for AL might cancel out their respective opposite effects on refractive error. In contrast, if the pleiotropic effects on AL could not compensate effects on CC, as shown in Group B, these variants may influence refractive error primarily through the net effect of CC. The interplay between AL, CC and refractive error at the variants in Group C is less clear, likely these variants might have pleiotropic effect for other endophenotypes, besides AL, to account for the genetic effects of CC on refractive error.

### Post GWAS gene-based and pathway analyses

We applied gene-based tests using the Versatile Gene-based Association Study (VEGAS)^25,26^, with Bonferroni corrected *p*-value at 2.09 × 10^−6^ to test 24,000 genes for significance. Over and above the loci found in the per-variant tests, six additional genomic regions were significantly associated with CC via gene-based tests (Supplementary Table 7): *ANKRD65* (*P* *=* 9.0 × 10^−7^), *PEAR1* (*P* *=* 1.57 × 10^−7^), *ASB1* (*P* *=* 2.54 × 10^−7^), *GMDS* (*P* *=* 2.48 × 10^−8^), *EMX2OS* (*P* *=* 4.84 × 10^−8^), and *HM13-AS1* (*P* *=* 2.18 × 10^−7^).

We further conducted gene-set analysis using VEGAS by testing whether CC genes shared a common function or operated in the same pathways (see Methods). Thirty pathways were identified at Bonferroni corrected *p*-value of 5.14 × 10^−6^ (Supplementary Data 4), with those involving proteinaceous extracellular matrix (ECM) (GO: 0005578; *P* *=* 1.05 × 10^−9^) and ECM (GO: 0031012, *P* *=* 7.53 × 10^−9^) being the top two. The other significant pathways included gene sets involved in eye development (GO:0001654; *P* *=* 4.00 × 10^−7^) and camera-type eye development, GO: 0043010, *P* *=* 3.20 × 10^−6^), as well as those involving in organ morphogenesis (GO:0009887; *P* *=* 4.86 × 10^−7^), skeletal system development (GO:0001501; *P* *=* 5.25 × 10^−7^) and the Wnt signalling pathway (KEGG:04310, *P* *=* 2.09 × 10^−6^). For the top 41 loci identified in this study plus genes previously reported for CC (*WNT7B*^24^ and *ZNRF3*^16^), we conducted gene-set analysis using g: Profiler (https://biit.cs.ut.ee/gprofiler/gost) and identified 48 significant pathways at multiple testing corrected *p*-value of 0.05. Among these, collagen-containing ECM (GO: 0062023; *P* = 2.72 × 10^−7^) and ECM (GO: 0031012; *P* = 6.51 × 10^−6^) were the most significant terms (Supplementary Data 5). Other significant pathways consisted of heparin binding (GO: 0062023; *P* = 6.95 × 10^−5^), embryonic morphogenesis (GO: 0048598; *P* = 3.99 × 10^−4^), glycosaminoglycan binding (GO: 0005539, *P* = 6.86 × 10^−4^), and skeletal system development (GO: 0001501; *P* = 1.11 × 10^−3^) etc.

**Fig. 5:**
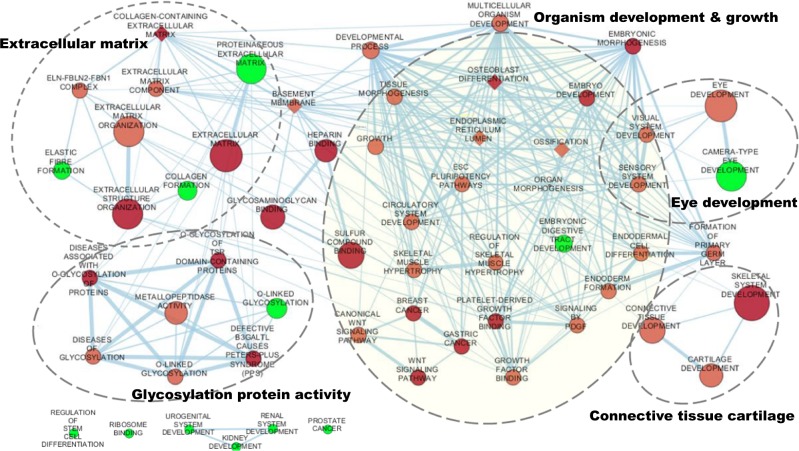
Gene-set enrichment analysis for corneal curvature in CREAM data. Enrichment results were mapped as a network of gene-sets (nodes) related by mutual overlap (edges). Node size is proportional to the total number of genes in each set, colour gradient represents the enrichment significance and edge thickness represents the number of overlapping genes between sets. Nodes in red represent gene-sets identified from the g:Profiler enrichment analysis, and in green represent additional gene-sets identified from the VEGAS-pathway analysis. Nodes of diamond show the pathways for the implicated genes associated with both CC and spherical equivalent (Group 2 in Fig. 3). Groups of functionally related gene-sets are circled and labelled (dashed line).

To visualize functional enrichment of identified gene-sets, we mapped these sets graphically into an enrichment network^27^ in Cytoscape^28^. Similarity coefficients greater than 0.375 were used to place these sets together with the interconnectivity drawn by a line. Using comprehensive collections of gene-sets related to CC, we identified enrichments in gene-sets involved in organism development and growth, with components for eye development and connective tissue cartilage, explicitly suggesting a strong genetic link between the size of the eye and organism/body (Fig. 5). We also observed that gene-sets were involved in ECM and glycosylation protein activity, which have previously been suggested in pathways related to central corneal thickness^29^.

To assess any specific roles of subset of CC genes with pleiotropic effects on myopia, we performed gene-set clustering. Enriched pathways identified included basement membrane, endoplasmic reticulum lumen, collagen-containing extracellular matrix, ossification, and osteoblast differentiation (Fig. 5; Diamond node; Supplementary Data 5). The pathways were closely connected to ECM or developmental process, thus underlying a functional heterogeneity in genes exhibiting pleiotropic effects on both CC and refractive error.

Additional pathways identified from the whole-genome data using VEGAS, such as elastic fibre formation, camera-type eye development and O-linked Glycosylation, also displayed connectivity to the ECM, development processes and glycosylation protein activity (Fig. 5; nodes in green). A few pathways (regulation of stem cell differentiation, system development, etc.) failed to link to any existing gene-sets were identified; this is likely attributable to marginal and uncharacterized CC-genes.

### Biological function of the CC-associated loci

We examined gene expression in 20 normal human donor eyes from the ocular tissue database (OTDB; https://genome.uiowa.edu/otdb/). The majority of the genes identified at the 41 loci were expressed in human ocular tissues including cornea, sclera, ciliary body, or lens etc. (Supplementary Data 6). *THBS4* had the highest expression in the cornea and sclera, *IGFBP5* in the ciliary body and sclera, and *PDGFRA* in the lens.

We also queried expression quantitative trait loci (e-QTL) database to assess the association between the gene expression and the top CC-variants in different human tissues (see UTLs). Twenty-one index variants were eQTLs for the expression of genes, which resided in, or were adjacent to, the variant (Supplementary Data 7). Among them, the majority of variants were eQTL’s for the expression of the nearest gene. There are some exceptions. For example, SNP rs807037 is a missense variant within the *KAZALD1* gene and is also an eQTL for the nearby gene *SFXN3* in artery, brain, adipose subcutaneous and nerve tissues (*P* < 2.20 × 10^−5^). Intronic *MTOR* rs3737611 is an eQTL for *EXOSC10* in thyroid, nerve and artery tissues (*P* *<* 5.70 × 10^−5^). Intronic *RSPO1* rs4074961 is an eQTL for itself and nearby genes *GNL2, DNALI1* and *MEAF6* in various tissues.

The newly identified genes are largely involved in ocular growth/development*. LMX1B* (encoding a LIM homeodomain class transcription factor) regulates anterior segment morphogenesis and patterning^30^, and is associated with Nail-Patella syndrome^31^. Recently, *LMX1B* has been reported to be associated with primary open-angle glaucoma, accompanied by development defects of the ocular anterior segments including cornea^32–34^. *SOX2* (encoding a member of the SRY-related HMG-box family of transcription factors) links to both anophthalmia and microphthalmia^35^. Other CC genes that are also associated with eye or overall morphology include *GHSR* (associated with craniofacial development^36^), *HMGA2* (linked to body height), and *LCORL* (linked to skeletal trunk height^37^).

Five of the implicated genes (*ADAMTS3*, *ADAMTS6*, *ADAMTS7, ADAMTS19*, and *ADAMTS20*) belong to the ADAMTS protein family, which is closely involved in regulating the organization and function of ECM^38^. For instance, *ADAMTS6* has a major role in focal adhesion and tight junction formation, and can alter the deposition of fibrillin microfibrils in epithelial cells^39^. Interestingly, ADAMTS family members also associate with height variation (*ADAM28, ADAMTS19, ADAMTS2, ADAMTS3, ADAMTS6, ADAMTSL1, ADAMTSL3*), suggesting their pleiotropic roles in body growth^40^.

A cluster of novel genes are involved in both ECM and organism/eye development. For instance, *FBN1* encodes the ECM protein fibulin-1, which modulates corneal cell migration by interactions with other ECM components, such as fibronectin^41^. Weill-Marchesani syndrome (that may be associated with thicker and steeper corneas) may also result from dominant mutations in *FBN1*. Fibronectin and IGFBP5 also bind to each other. This binding regulates the ligand-dependent action of IGFBP5 on insulin-like growth factors, and this has effects on cell proliferation, differentiation, survival, and motility. *IGFBP5* shows expression in the human cornea and was down regulated in eyes with keratoconus^42,43^. *BMP7* encodes a member of the transforming growth factor -β superfamily that is involved in numerous cellular functions including development, morphogenesis, cell proliferation, apoptosis, and ECM synthesis^44^. *BMP7* is key in eye development during embryogenesis, and *BMP7*-knockout mice have been shown to develop anophthalmia^45,46^. *OFCC1* (encoding a reticular cytoplasmic protein expressed during embryonic development) in the Medaka fish is associated with the *ojoplano* (‘flat eye’) phenotype due to defective eye cup morphogenesis^47^. *COL5A1* and *COL6A1* encode components of type V and VI fibrillar collagens that are present in the human cornea^48^. *COL6A1*, together with *ADAMTS20*, have been reported to be associated with intraocular pressure^49^. *COL5A1* mutations are found in classical Ehlers-Danlos Syndrome^50^, which is associated with thinner and steeper corneas^51^. *COL5A1* is also a susceptibility locus for central corneal thickness^52,53^. *THBS4* encodes an extracellular calcium binding protein that is involved in cell proliferation, adhesion, and migration^54^. *FGF9* is involved in the neural patterning of the optic neuroepithelium^55^.

Genes identified as being involved in the Wnt signaling pathway were also implicated in our analysis (*SOX2, FRAT1, FRAT2, RSPO1, FGF9*; Supplementary Data 5 “canonical Wnt signaling pathway”). Two additional Wnt signalling related genes (*ZNRF3*^16^ and *WNT7B*^24^), though not the top genes in this study, were also identified as being associated with CC or axial length. The Wnt signalling pathway has prominent effects on multiple developmental events during embryogenesis^56^, including that of differentiation of the anterior segment of the eye^57,58^, and retinal development^59,60^.

## Discussion

In the largest CREAM trans-ethnic GWAS meta-analysis of CC to date (44,042 individuals with replication in 88,218 participants from UK Biobank), we identified novel loci through single-variant analysis, and gene-based tests. SNP-heritability was estimated at 0.267 and 0.196 in Europeans and Asians, respectively. We discovered population-specific loci that existed in both European and Asian ethnic groups, as well as the presence of a high concordance of inter-population genetic effects overall. Variants were involved in coordinating eye size (by affecting CC and AL concurrently) whilst maintaining emmetropia (Group A). Meanwhile, other genetic variants were associated with refractive error (Group B); the genetic effect for AL could not compensate the effect for CC, as showing that the pleiotropic effect ratio $\frac{{{\upbeta}_{\mathrm{AL}}}}{{{\upbeta}_{\mathrm{CC}}}}$ was significant smaller than that of “eye size” variants. A third group of variants was also observed that appeared independent in terms of pleiotropic effects. Besides pathways related to the ECM, the implicated genes were significantly enriched in pathways involved in organism development and growth, eye development, connective tissue cartilage and glycosylation protein activity. Implicated genes with pleiotropic effects on refractive error were involved in diverse pathways related to ECM and organism development and growth.

Our data provide insights into novel genes that regulate CC across European and Asian populations. We found trans-ethnic replication of significant loci, and a high concordance of genetic effects in variants with little discrepancy in allele frequency between the two ancestry groups. Our results are robust as 90.2% of CC-associated loci were replicated at a genome-wide significance throughout the UK Biobank. We also confirm the association of the *MTOR* loci^17^ with CC in Europeans, in contrast to the lack of replication in previous Europeans studies with much smaller sample size^11,19^. Although the underlying genetic effects were largely shared between the two ancestry groups, at the same time, population-specific loci were also observed: *HDAC11/FBLN2, ADAMTS19/CHSY3*, and *FGF9* in Europeans, and *RBP3* and *CMPK1/STIL* previously reported in Asians^18^. In these cases, the lead variants were monomorphic in the other non-significant Asian or European populations, respectively, and any signals barely seen from the flanking variants at these loci. Our data show that the trans-ethnic meta-analysis approach yields shared and unique variants for CC in Europeans and Asians.

We used bioinformatics tools to demonstrate the functional connectivity between the associated genes. The newly identified loci such as *LMX1B*, *SOX2*, *NHSL1*, *GHSR, HMGA2, IGFBP5, FRAT1, FRAT2, STIL, USP1, HUS1, STON2,* and *IGF2* are mainly involved in organism growth and eye development. Additional notable CC candidate genes belong to the ADAMTS family, including *ADAMTS7, ADAMTS19*, and *ADAMTS20* involved in organization and function of ECM. Novel genes, such as *FBN1, BMP7, COL6A1, THBS4, FBLN2, and KAZALD1*, are involved in both ECM formation and organism development. In addition, CC-genes associated with refractive error were involved in basement membrane, endoplasmic reticulum lumen, collagen-containing extracellular matrix, ossification, and osteoblast differentiation, underlying a functional heterogeneity in genes exhibiting pleiotropic effects on both CC and refractive error.

Our study is the most comprehensive study on pleiotropic effects of CC-associated genes on eye size and refractive error in humans. Several small-size studies also have reported the effects of ‘eye-size’ genes, such as *PDGFRA*^11,17,19^ and *RBP3*^18^. Our study confirmed previous findings. Studies in mice and chicken also support the existence of distinctive genetic effects to determine eye sizes, for instance, (1) effects that purely govern eye size, (2) effects restricted to specific ocular dimension (i.e. CC or AL separately), or (3) effects that scale the size of the eye and body simultaneously^61,62^. Clearly, CC-genes are involved in heterogeneous genetic function.

In human emmetropic eyes, CC is highly correlated with AL, and the two are carefully scaled relative to each other^63^. Thus, genetic pathways may exist to simultaneously influence AL and CC while maintaining the emmetropic status^12,61,64^. In our analyses, we have therefore compared our set of CC loci with their respective associations with AL and refractive error. We identified ‘eye-size’ genes (*HMGA2*, *RSPO1, HDAC11/FBLN2, RBP3, PDGFRA, NHSL1* and *ADAMTS19/CHSY3*, and *INTS6*) that were associated with eye size (e.g. a larger eye with both a flatter CC and longer AL, or vice versa), but not refractive error. The compensatory pleiotropic effect for AL could offset CC’s effect toward myopia or hyperopia; namely, a genetic determined 1 mm increase (or decrease) for CC accompanying a 2.92 mm increase (or decrease) on average for AL might cancel out their opposite effects on refractive error. This may represent a carefully coordinated scaling of optical components to maintain the eye in an emmetropic state as it grows. These genetic variants may therefore control variation in eye size independent of refractive error. Among these ‘eye-size’ genes, *HMGA2* and *HDAC11/FBLN2* are likely to have pleiotropic effects on both the coordinated scaling of the eye, as well as height^65,66^. Similarly, the *ADAMTS19* gene encodes metalloproteinases that belong to the *ADAMTS* family with the members as human growth genes^40^. This is consistent with the findings that height (body size) and eye size are genetically coordinated^12,62^. Among the other ‘eye-size’ genes identified, some had roles in Wnt signalling (*RSPO1* and *HDAC11*), platelet-derived growth factor signalling (*PDGFRA*), and extracellular ligands and calcium binding (*FBLN2*).

In contrast to the group of ‘eye-size’ genes that do not affect spherical equivalent, there is another group of CC-implicated variants associated with refractive error, with little or no pleiotropic effect on AL (Group B). The pleiotropic ratio $\frac{{{\upbeta}_{\mathrm{AL}}}}{{{\upbeta}_{\mathrm{CC}}}}$ was significantly smaller than that in ‘eye-size’ variants, therefore, without adequate compensatory effects on AL, these variants may influence the refractive error status of the eye primarily through CC. There is one exception at loci *RP11-91P17.1*. Variant rs7000412 T allele was associated with steeper CC (that tends to make the eye more myopic) and shorter AL (less myopic) with overall effects towards to a hyperopic refractive error; thus this variant influenced refractive error likely through AL. Five top loci replicated in UK Biobank for refractive error (*P* < 1 × 10^−7^), including *CHRND/RPSS56*, *FBN1*, *CASC15*, *RNLS*, and *KAZALD1*, have also been reported in previous CREAM GWAS^13^. Ten loci showed significance in replication after accounting for multiple testing (FDR < 0.01). These genes are actively involved in pathways of basement membrane, endoplasmic reticulum lumen and collagen-containing extracellular matrix, linking to ECM and organism development, growth. Plotnikov et al. recently also proposed a genetic link between CC and refractive error in Europeans^67^. Using CC-associated SNPs in emmetropes as instrument variables, they estimated the causal effect of CC on refractive error to be +1.41 D (95% CI, 0.65–2.16) less myopic refractive error per mm flatter cornea. A significant group of CC-genes identified in our study showing association with spherical equivalent corroborates the finding of an association between CC and refractive error.

The remaining CC loci (Group C) were not significantly associated with refractive error or AL. However, in a majority of these variants, the associations with spherical equivalent, although not statistically significant, were in the expected direction—for instance, a flatter cornea and a more hyperopic spherical equivalent, or vice versa. It is unknown whether these loci may also have modulatory effects on other refractive components of the eye (e.g. lens thickness or anterior chamber depth) that may have attenuated its effect on refractive error. In addition, some of these genes in Group 3 (*FNDC3B, COL5A1, COL6A1*), together with genes in Group 2 (*IGFBP5, FGF9*, and *CWC27/ADAMT6*) was linked to connective tissue disorder (Supplementary Fig. 6) and has been associated with keratoconus^29,68–71^, a disorder of corneal thinning and steepening, implying a possible effect of CC genes regulating refractive error and keratoconus.

In summary, we have identified 47 genome-wide significant loci for CC (of which 26 are new), through a large-scale tans-ethnic GWAS meta-analysis. The importance of undertaking this study in individuals of different ethnicities cannot be understated as we identified both population-specific loci in Europeans as well as Asians as well as loci that were common between both ethnicities. These findings provide insights into the underlying genetic aetiology of eye growth and may provide pointers for us to explore why myopia is more prevalent in Asians than Europeans. These CC loci can coordinate AL and eye-size to keep human eyes emmetropic, and some play a role in the development of refractive errors primarily through variations in CC. Implicated genes were significantly enriched in a network linking extracellular matrix organization, developmental process for body and eye and glycosylation protein activities. Elucidating and characterising the heterogeneity of such genes that regulate the optical component dimensions of the eye may enable a better understanding of the biology of both emmetropia and ametropia in humans.

## Methods

### Study populations

The discovery cohorts included 29,580 individuals with European ancestry from 18 studies, and 14,464 with Asian ancestry from 10 studies. General methods, demographics, and phenotyping of the study cohorts have previously been extensively described, and are provided in brief in Supplementary Table 1 and Supplementary Notes. In the replication phase, 88,218 participants of European ancestry from the UK Biobank who had measurements for CC were included in the replication stage, as well as 95,505 participants of European ancestry (from the UK Biobank) with phenotype information for refractive error^3^. Written informed consent was obtained from all participants in accordance with the Declaration of Helsinki. All studies were performed with the approval of their local Human Research and Ethics Committee.

### Phenotype measurements

All participating CREAM cohorts used similar protocols for the collection of keratometry and other ocular biometric measurements. The protocols have been described in detail elsewhere^14,16,59^. In brief, CC radii in the horizontal and vertical meridians were measured using an autokeratometer. The means (in millimetre) of CC from the individuals’ two eyes were used for analysis, while the means of the readings from one eye were used when the readings from the other eye were unavailable. Participants were excluded if they had corneal scars, keratoconus, prior refractive or cataract surgery, or other intraocular procedures that could alter CC. For the UK Biobank, participants were excluded from the analyses if they had an eye disorder that may have altered their refractive error or CC (see Supplementary Notes).

### Genotyping and imputation

The CREAM study samples were genotyped on either Illumina or Affymetrix platforms. Genotypes were imputed using the 1000 G Genomes Project reference panel (Phase I version 3, March 2012 release). SNPs with low imputation quality were filtered using metrics specific to the imputation method and thresholds used in previous GWAS analyses. The Markov Chain Haplotyping software, IMPUTE^72,73^, or MACH^74^ were adopted for imputation. A detailed description regarding genotyping platforms and imputation procedures have been outlined (Supplementary Table 2). Stringent quality control of genotype data was applied in each cohort from CREAM. Samples with low call rates (<95%) or with gender discrepancies were excluded. Cryptically related samples and outliers in population structure from principal component analyses were also excluded. SNPs flagged with missingness >5%, gross departure from Hardy-Weinberg equilibrium (*P* < 10^−6^), and minor allele frequency (MAF) < 1% were removed from further analyses. Poorly imputed markers (IMPUTE info < 0.5 or minimac Rsq < 0.5) were excluded. UK Biobank genotyping arrays were imputed to the HRC reference panel and a combined 1000 Genomes Project –UK10K reference panel using IMPUTE4^75^. Data quality control (QC) was described in the Supplementary Notes.

### Statistical analyses and meta-analyses

We assumed an additive genetic model where the dosage of each SNP was a continuous variable ranging from 0 to 2 for the effect allele. For each study, an additive allele-dosage regression model, adjusted for both age and sex at each genotyped or imputed SNP, was conducted to determine its association with CC represented as a quantitative trait. An additional adjustment for up to the first five principal components was carried out according to the population substructure in each individual study. For studies that included children and adolescent participants, GWAS analyses were conducted separately by age groups (age ≥ 25 vs. age < 25), as for previous GWAS analyses for corneal astigmatism^76^. Sample outliers with CC values exceeding six standard deviations from the mean were excluded at the study level. The per-SNP meta-analyses were performed in METAL software (https://genome.sph.umich.edu/wiki/METAL) with a weighted inverse-variance approach^77^. A Cochran’s Q test was used to assess heterogeneity across studies^78^.

### Locus identification and genetic variants annotation

The independent signal from the meta-analysis was determined using LD-clumping procedure in PLINK (https://www.cog-genomics.org/plink2). The index variant was identified (*P* < 5 × 10^−8^) in each clump, which was formed for variants with *P* < 1 × 10^−5^ that were in LD (r^2^ > 0.1) and within 500 kb of the index variant. The same variant were assigned to no more than one clump. The LD structure was estimated from the European panel in the 1000 Genome Project as the reference population, or Asian panel for meta-analysis summary statistics in Asians. A locus was identified by an index variant with the regions flanking 250 kb on both sides. For those with multiple signals in one locus (500 kb region) or an overlapping of multiple loci identified from the PLINK clumping procedure, conditional analysis was further performed to confirm the independent signals using GCTA-COJO^79^. The LD structure was estimated in the same manner as for LD-clumping procedure in PLINK. The regional plot was drawn for each identified locus from the combined meta-analysis (Supplementary Fig. 4) using LocusZoom (http://locuszoom.org/).

The coordinates and variant identifiers are reported on the NCBI B37 (hg19) genome build, and annotated using UCSC Genome Browser^80^. We identified variants within each of the LD blocks (*r*^2^ ≥ 0.6) in European and Asian populations of the 1000 Genomes Project (100 Kb flanking the top SNP at each locus) to apply functional annotations of transcription regulation using HaploReg^81^ (https://pubs.broadinstitute.org/mammals/haploreg/haploreg_v3.php) and Encyclopedia of DNA Elements (ENCODE)^82^ data.

### Replication in UK Biobank participants

The UK Biobank reported the maximum and minimum corneal power in each eye. After taking the mean of replicate readings, the corneal power in each eye was calculated as the mean of the maximum and minimum values. Corneal power was converted to corneal radius of curvature using the equation CC = (337.5/corneal power). For the genetic analysis of CC in all available participants, we took the average CC of the two eyes as the phenotype. A total of 88,218 individuals were included in the analysis for CC in all available participants. Analyses were performed using BOLT v2.3^22^. Variant genotype, age, sex, genotyping array (coded as 0 or 1 for the UK BiLEVE or UK Biobank Axiom, respectively) and the first 10 PCs were included as covariates. BOLT uses a mixed model to account for relatedness (kinship) between individuals.

### Gene-based tests and pathway analyses

Gene-based testing was conducted using the VEGAS software^25,26^ (https://vegas2.qimrberghofer.edu.au/) on the results of separate meta-analyses of GWAS in European and Asian ancestries. Gene-based *p*-values from different populations were combined by Fisher’s method. For samples of European descent, we used the European panel in the 1000 Genome Project as the reference population to estimate patterns of LD. For the Asian ancestry groups, we used the combined 1000 Genome Project Asian samples as the reference population to approximate LD patterns. To include gene regulatory regions, SNPs were included if they fell within 50 kb of the transcription start site of genes.

VEGAS-Pathway analysis^25,26,83^ was carried out with pre-specified pathways from Gene Ontology^84^, MSigDB^85^ (containing canonical pathways and gene-sets from BIOCARTA, REACTOME, KEGG databases), PANTHER^86^, and pathway commons databases^87^. We filtered these gene-sets to include only pathways with 10–1000 genes, yielding 9734 pathways. Empirical VEGAS-Pathway *p* values for each pathway were computed by comparing the summed *χ*^2^ test statistics from real data with those generated in 500,000 simulations where the relevant number (according to the size of the pathway) of randomly drawn *χ*^2^ test statistics was summed. To ensure that clusters of genes did not adversely affect results within each pathway, gene-sets were pruned such that each gene was >500 kb away from all other genes in the same pathway. We performed meta-analysis on the two sets of pathway *p*-values from Asian and European samples by Fisher’s method.

We investigated functional annotation of the top identified genes using g:Profiler (https://biit.cs.ut.ee/gprofiler/gost). For g:Profiler, we used “g:GOST” function to perform pathway analysis on identified CC-associated genes. Pre-specified pathways include Gene Ontology, pathways from KEGG, Reactome, WikiPathways and protein complexes from CORUM^88^. The significant pathway was claimed at the adjusted *p*-value < 0.05 after correction for multiple testing.

To investigate the connection between the enriched gene-sets, we mapped these gene-sets into network functional enrichment map analysis^27^. We visualize the network enrichment in Cytoscape software v3.7.1 (https://cytoscape.org/)^28^. Highly similar gene-sets were placed close together with the interconnectivity among gene-sets drawn by line (edge; similarity coefficient > 0.375).

### Association of corneal curvature loci with axial length and spherical equivalent

For all identified CC-associated variants, we assessed their association with refractive error in European participants of UK Biobank (*N* = 95,505). We further assessed the association of CC-associated variants with AL using a subset of CREAM cohorts (*N* = 10,851; Supplementary Table 5). False discovery rates (FDR) from the Benjamini-Hochberg procedure were set at 1% as a threshold of statistical significance^23^. We categorized CC variants into three groups: (A) variants were associated with AL, but not spherical equivalent; (B) variants were associated with spherical equivalent; and (C) variants were not associated with spherical equivalent or AL.

Pleiotropic effect ratio $\frac{{{\upbeta}_{\mathrm{AL}}}}{{{\upbeta}_{\mathrm{CC}}}}$ at each variant was calculated to quantify relevant genetic effects on AL versus effects on CC and the variance was calculated using Delta method. To estimate the pleiotropic effect ratio for variants in each group, we performed meta-analyses in METAL software (https://genome.sph.umich.edu/wiki/METAL) with a weighted inverse-variance approach^77^. A Cochran’s Q test was used to assess heterogeneity across variants^78^. Z-statistics were used to test the significant difference of the pleiotropic ratio $\frac{{{\upbeta}_{\mathrm{AL}}}}{{{\upbeta}_{\mathrm{CC}}}}$ .

### SNP-heritability estimation

We applied the LD score method^22^ (https://github.com/bulik/ldsc) using GWAS summary statistics to estimate SNP-*h*^2^. After merging SNPs with the HapMap3 Asian samples, we had a total of 1,174,487 and 1,085,659 SNPs for the LD score regression analyses for the European and Asian populations, respectively. The LD score matrix was estimated from the 1000 Genomes Project Asian reference panel, or European reference panel separately, with a 1 cM sliding window. We calculated the heritability using the software Idsc v1.0.0. The resulting regression slope was multiplied by the number of effective SNPs in the reference panel from the 1000 Genomes Project data^22^.

### Gene expression in human ocular tissues

To assess gene expression in human tissues, we examined the Ocular Tissue Database (OTDB) (https://genome.uiowa.edu/otdb/) and the EyeSAGE database^89,90^ (http://people.duke.edu/~bowes007/EyeSAGE.htm). The estimated gene and exome level abundances are available online. Normalization of gene expression used the PLIER method with GC-background correction^89^. Relationships between genotype and *cis* regulation of gene expression levels were assessed using expression quantitative trait locus (eQTL) associations obtained from GTEx Portal database^91^ (https://gtexportal.org/home/).

### Reporting summary

Further information on research design is available in the Nature Research Reporting Summary linked to this article.

## Supplementary information

Supplementary Information

Description of Additional Supplementary Files

Supplementary Data 1

Supplementary Data 2

Supplementary Data 3

Supplementary Data 4

Supplementary Data 5

Supplementary Data 6

Supplementary Data 7

Supplementary Data 8

Reporting Summary

## Data Availability

The summary statistics of the meta-analysis combining studies in CREAM are included in Supplementary Data 8. To protect the privacy of the participants in our cohorts, the datasets generated during and/or analysed during the current study are available from the corresponding authors on reasonable request.
