# Supplementary material for: Genome-wide association meta-analysis of corneal curvature identifies novel loci and shared genetic influences across axial length and refractive error
Source: Commun Biol. 2020 Mar 19;3:133. doi: 10.1038/s42003-020-0802-y (PMC7081241; doi:10.1038/s42003-020-0802-y)
Supplement: Supplementary file 1 — Supplementary Information [file 42003_2020_802_MOESM1_ESM.pdf]

SUPPLEMENTARY MATERIALS

Q. Fan et al. Genome-wide association meta-analysis of corneal curvature identifies novel loci and shared genetic influences across axial length and refractive error

CONTENTS

Supplementary Figures

Supplementary Figure 1. Quantile-Quantile plot of the meta-analysis of GWAS results in all cohorts

Supplementary Figure 2. Manhattan plot and QQ plot of the meta-analysis of GWAS results in Europeans

Supplementary Figure 3. Manhattan plot and QQ plot of the meta-analysis of GWAS results in Asians

Supplementary Figure 4. Regional association plots for the 42 identified loci

Supplementary Figure 5. Overlap of genes associated with corneal curvature, axial length and spherical equivalent

Supplementary Figure 6. The network shows functional annotation of genes at top loci

Supplementary Tables

Supplementary Table 1. Study cohorts and summary of corneal curvature measures (total n =44,042)

Supplementary Table 2. Genotyping and imputation information for the included cohorts

Supplementary Table 3. Association for corneal curvature at proxy SNPs for those monomorphic SNPs in Table1

Supplementary Table 4. Additional genome-wide significant hits when Europeans and Asians analysed separately

Supplementary Table 5. CREAM cohorts to test association between identified variants and axial length

Supplementary Table 6. Pleiotropic effect ratio estimation for CC-variants

Supplementary Table 7. Significant Loci for corneal curvature identified through the gene-based tests

Supplementary Notes:

Study Description & Acknowledgements

Supplementary Figure 1. Quantile-Quantile plot of the meta-analysis of GWAS results in all cohorts

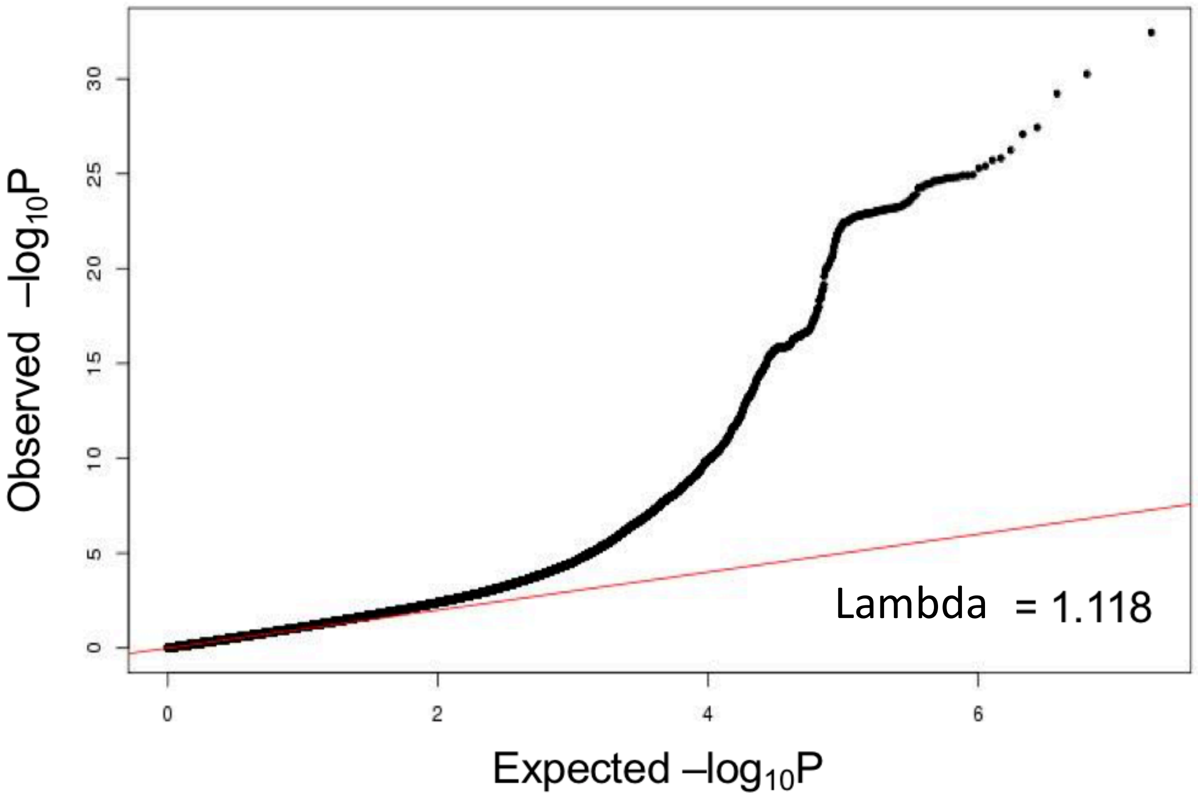

Supplementary Figure 2. Manhattan plot and QQ plot of the meta-analysis of GWAS results in Europeans

A. Manhattan plot

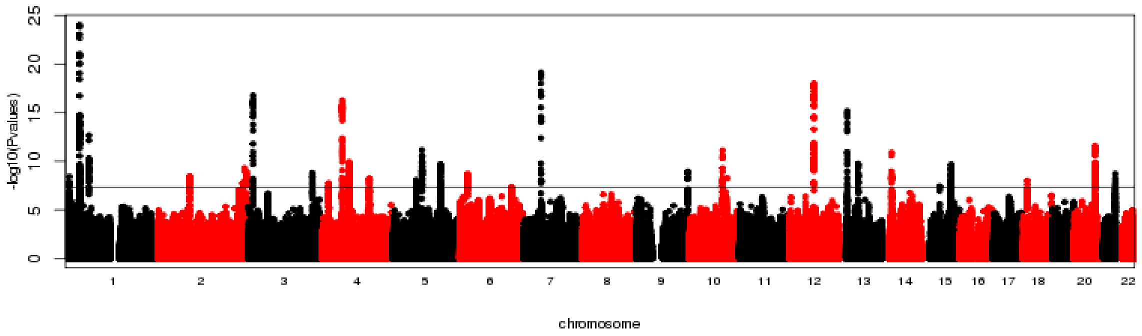

B. QQ plot

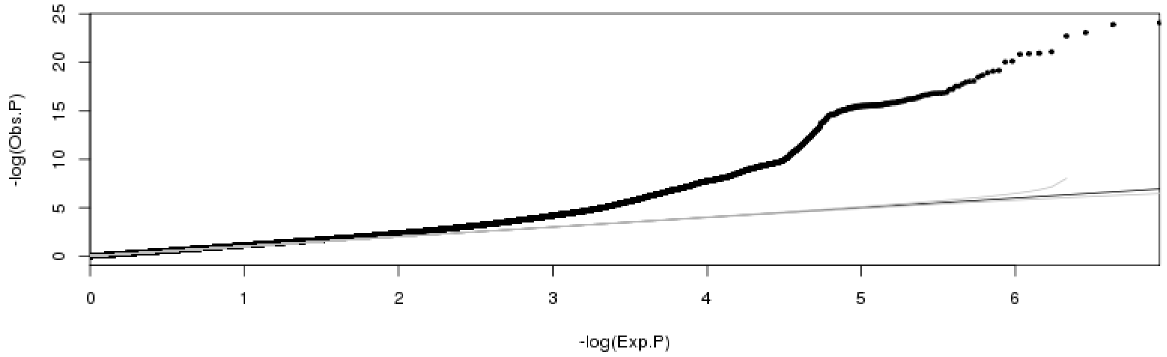

Supplementary Figure 3. Manhattan plot and QQ plot of the meta-analysis of GWAS results in Asians

A. Manhattan plot

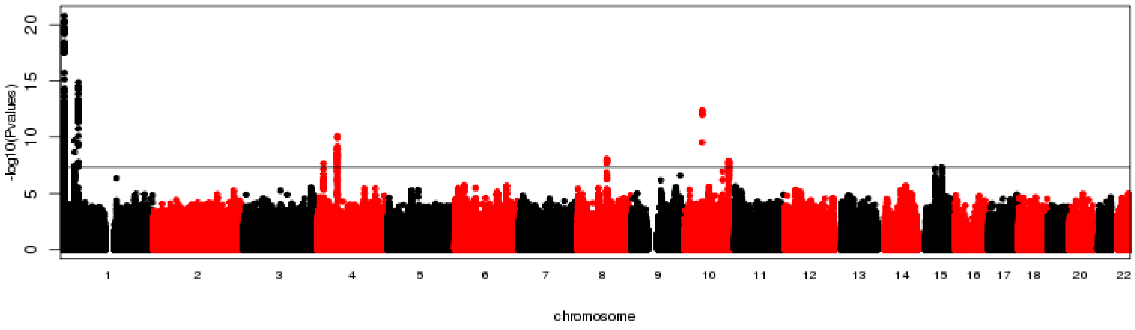

B. QQ plot

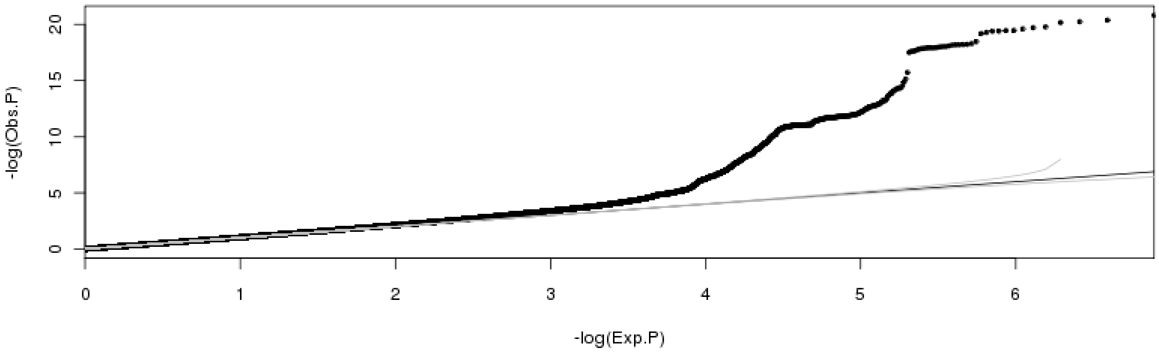

Supplementary Figure 4. Regional association plots for the 41 identified loci LD (r^2) shown in the plots based on European populations

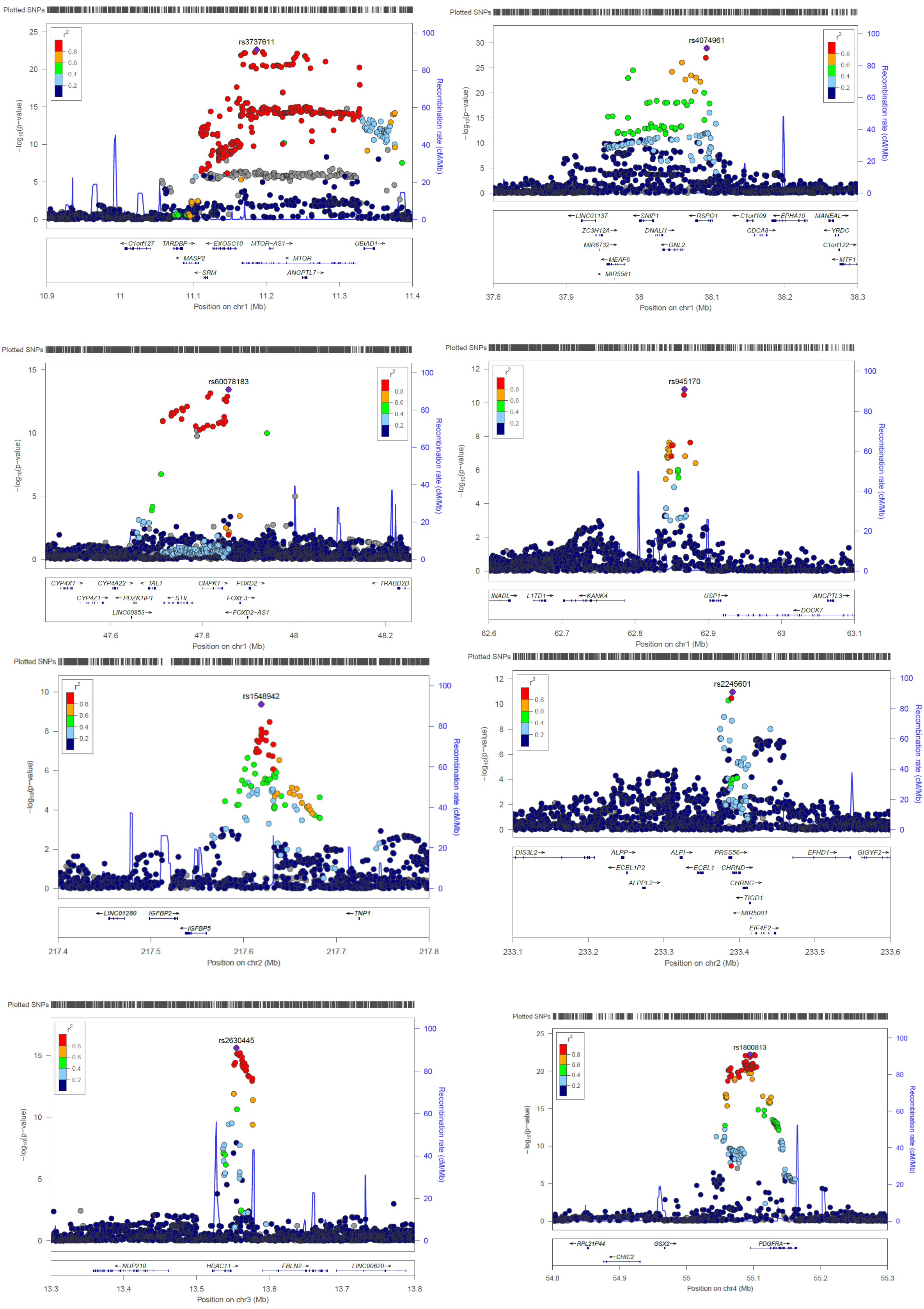

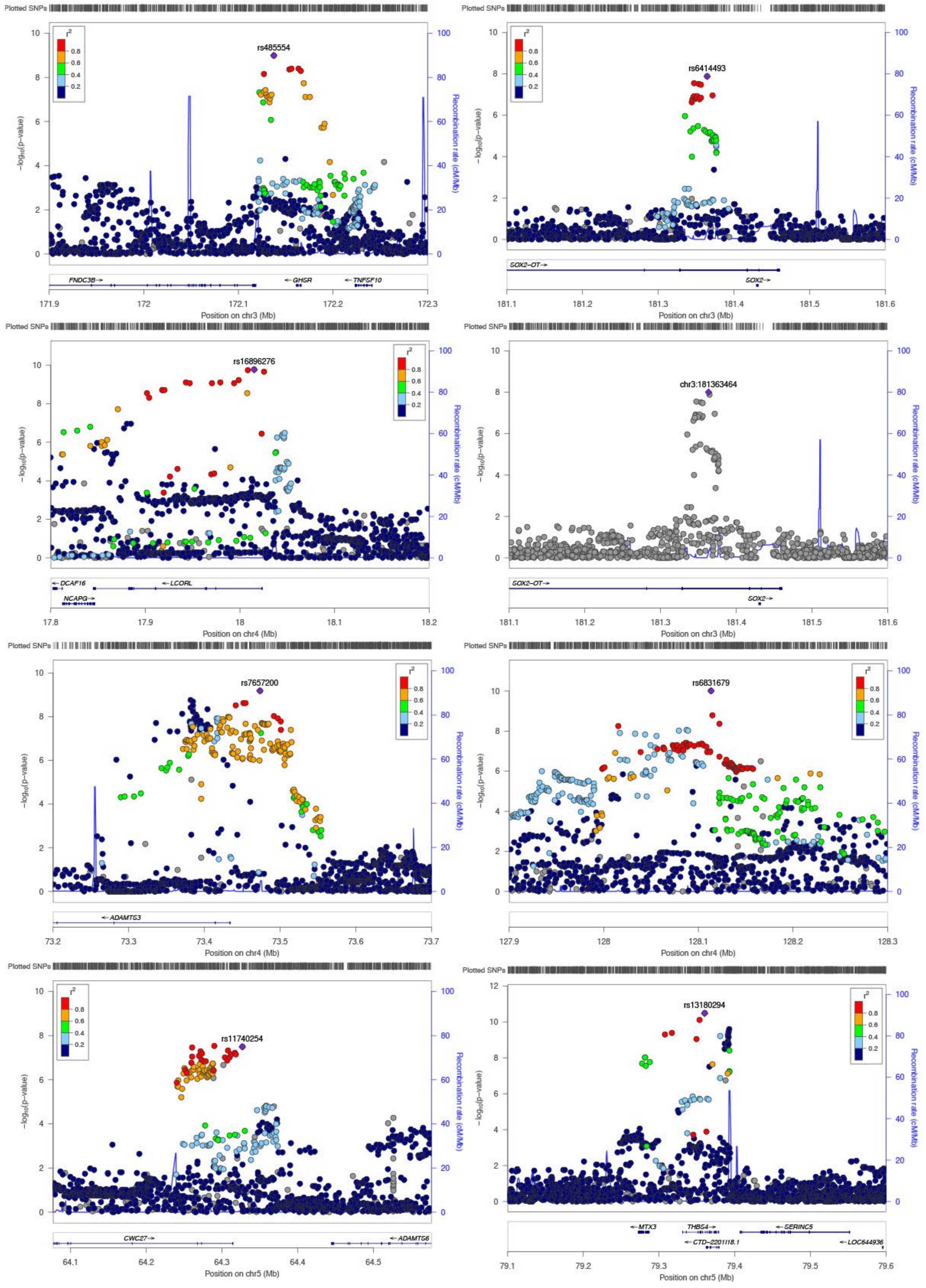

Plotted SNPs
rs485554
FNDC3B→
←GHSR
←TNFSF10
Position on chr3 (Mb)
rs6414493
SOX2−OT→
SOX2→
Position on chr3 (Mb)
rs16896276
←DCAF16
NCAPG→
←LCORL
Position on chr4 (Mb)
chr3:181363464
SOX2−OT→
SOX2→
Position on chr3 (Mb)
rs7657200
←ADAMTS3
Position on chr4 (Mb)
rs6831679
Position on chr4 (Mb)
rs11740254
CWC27→
←ADAMTS6
Position on chr5 (Mb)
rs13180294
←MTX3
THBS4→
←SERINC5
←CTD−2201I18.1
←LOC644936
Position on chr5 (Mb)
−log10(p−value)
Recombination rate (cM/Mb)
r²
0.8
0.6
0.4
0.2

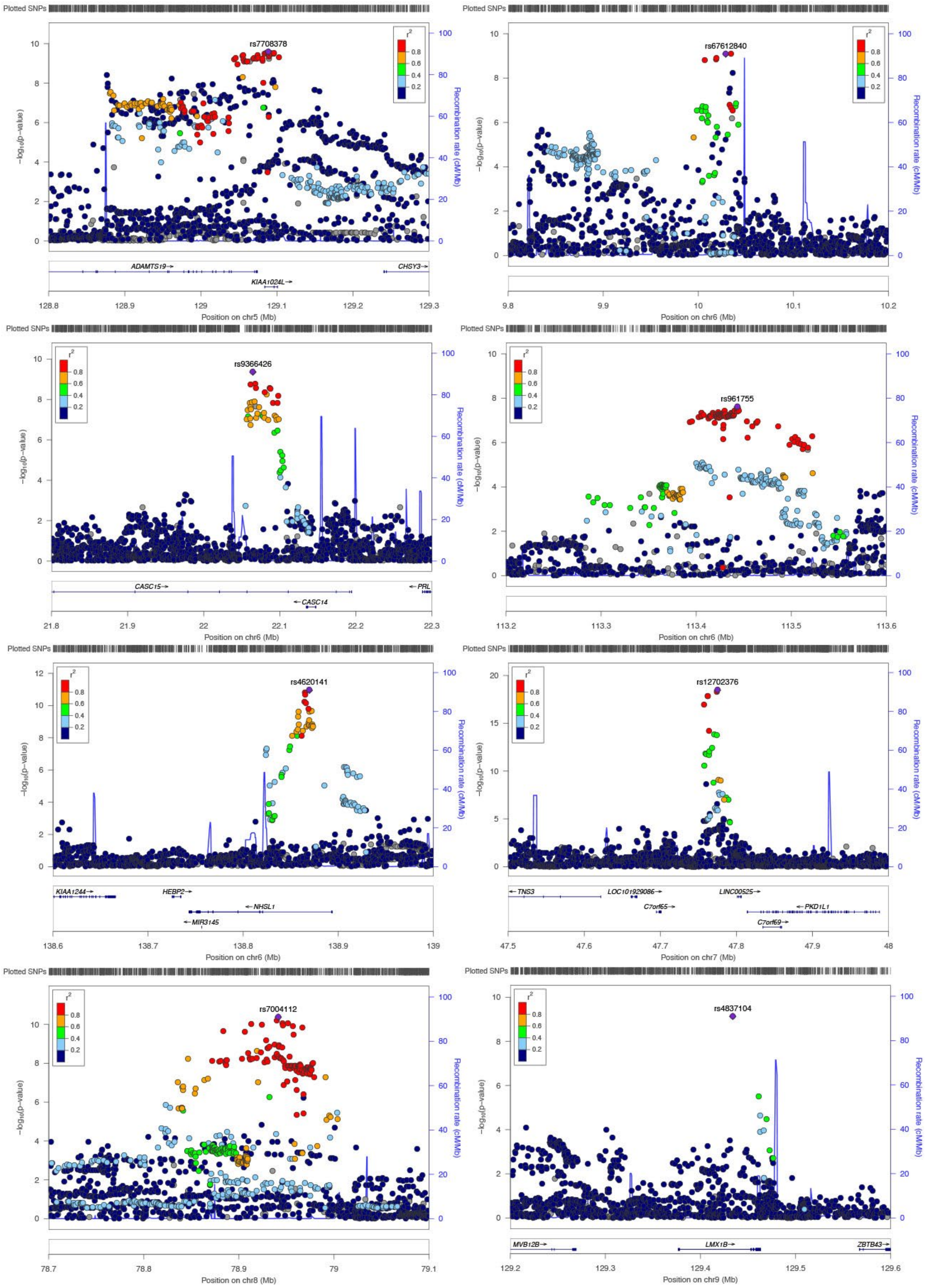

Plotted SNPs
rs7708378
ADAMTS19
KIAA1024L
CHSY3
Position on chr5 (Mb)
rs67612840
Position on chr6 (Mb)
rs9366426
CASC15
CASC14
PRL
Position on chr6 (Mb)
rs961755
Position on chr6 (Mb)
rs4620141
KIAA1244
HEBP2
NHSL1
MIR3145
Position on chr6 (Mb)
rs12702376
TNS3
LOC101929086
C7orf65
LINC00525
PKD1L1
C7orf69
Position on chr7 (Mb)
rs7004112
Position on chr8 (Mb)
rs4837104
MVB12B
LMX1B
ZBTB43
Position on chr9 (Mb)
$-\log_{10}(p\text{-value})$
Recombination rate (cM/Mb)
r^2

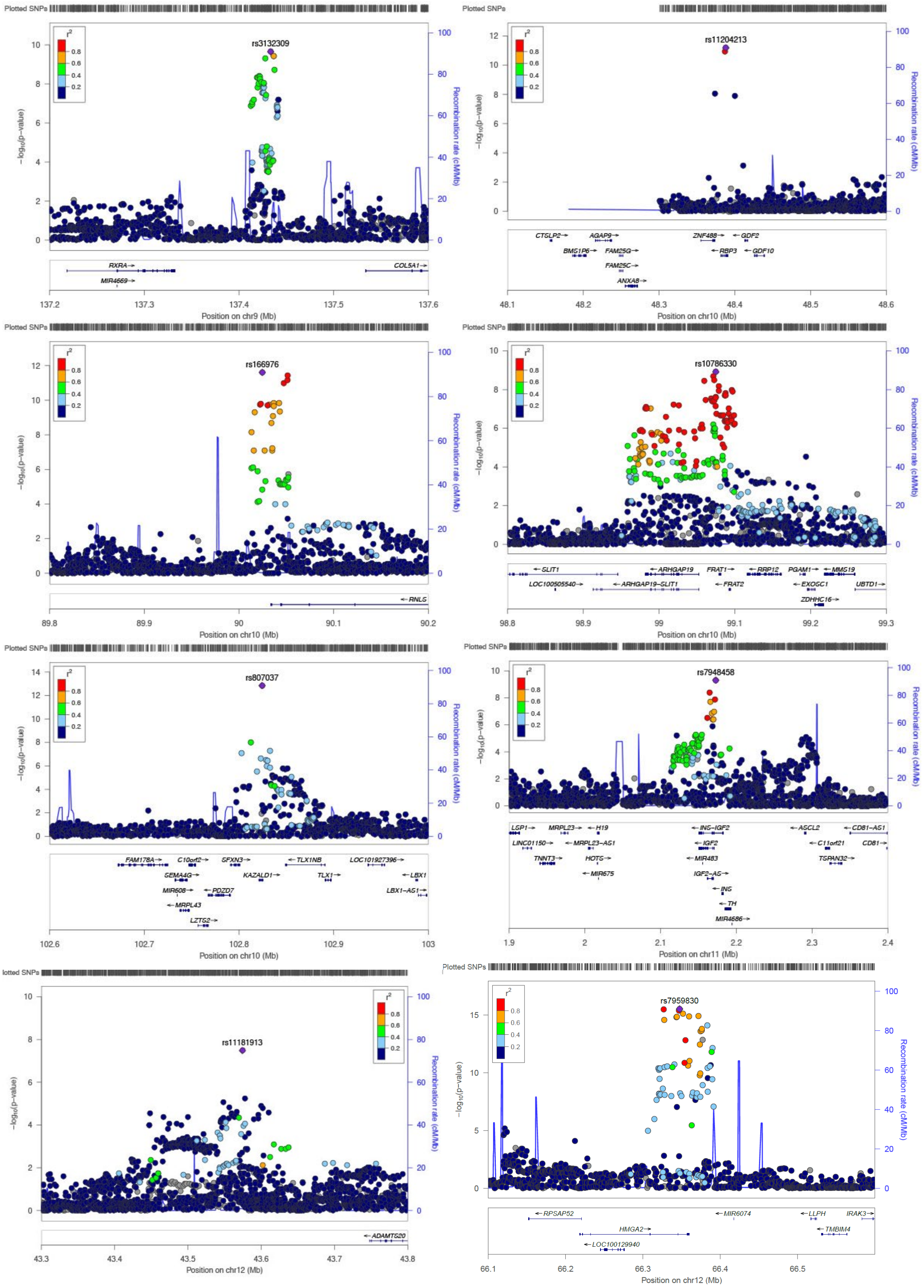

Plotted SNPs
rs3132309
RXRA
MIR4669
COL5A1
Position on chr9 (Mb)
rs11204213
CTGLP2
AGAP9
BMS1P6
FAM25G
FAM25C
ANXA8
ZNF488
RBP3
GDF2
GDF10
Position on chr10 (Mb)
rs166976
RNLS
Position on chr10 (Mb)
rs10786330
SLIT1
LOC100505540
ARHGAP19
ARHGAP19-SLIT1
FRAT1
FRAT2
RRP12
PGAM1
EXOSC1
MMS19
UBTD1
ZDHHC16
Position on chr10 (Mb)
rs807037
FAM178A
C10orf2
SFXN3
TLX1NB
LOC101927396
SEMA4G
KAZALD1
TLX1
LBX1
MIR608
PDZD7
LBX1-AS1
MRPL43
LZTS2
Position on chr10 (Mb)
rs7948458
LSP1
MRPL23
H19
INS-IGF2
ASCL2
CD81-AS1
LINC01150
MRPL23-AS1
IGF2
C11orf21
CD81
TNNT3
HOTS
MIR483
TSPAN32
MIR675
IGF2-AS
INS
TH
MIR4686
Position on chr11 (Mb)
rs11181913
ADAMTS20
Position on chr12 (Mb)
rs7959830
RPSAP52
MIR6074
LLPH
IRAK3
HMGA2
TMBIM4
LOC100129940
Position on chr12 (Mb)
−log10(p-value)
Recombination rate (cM/Mb)
r2

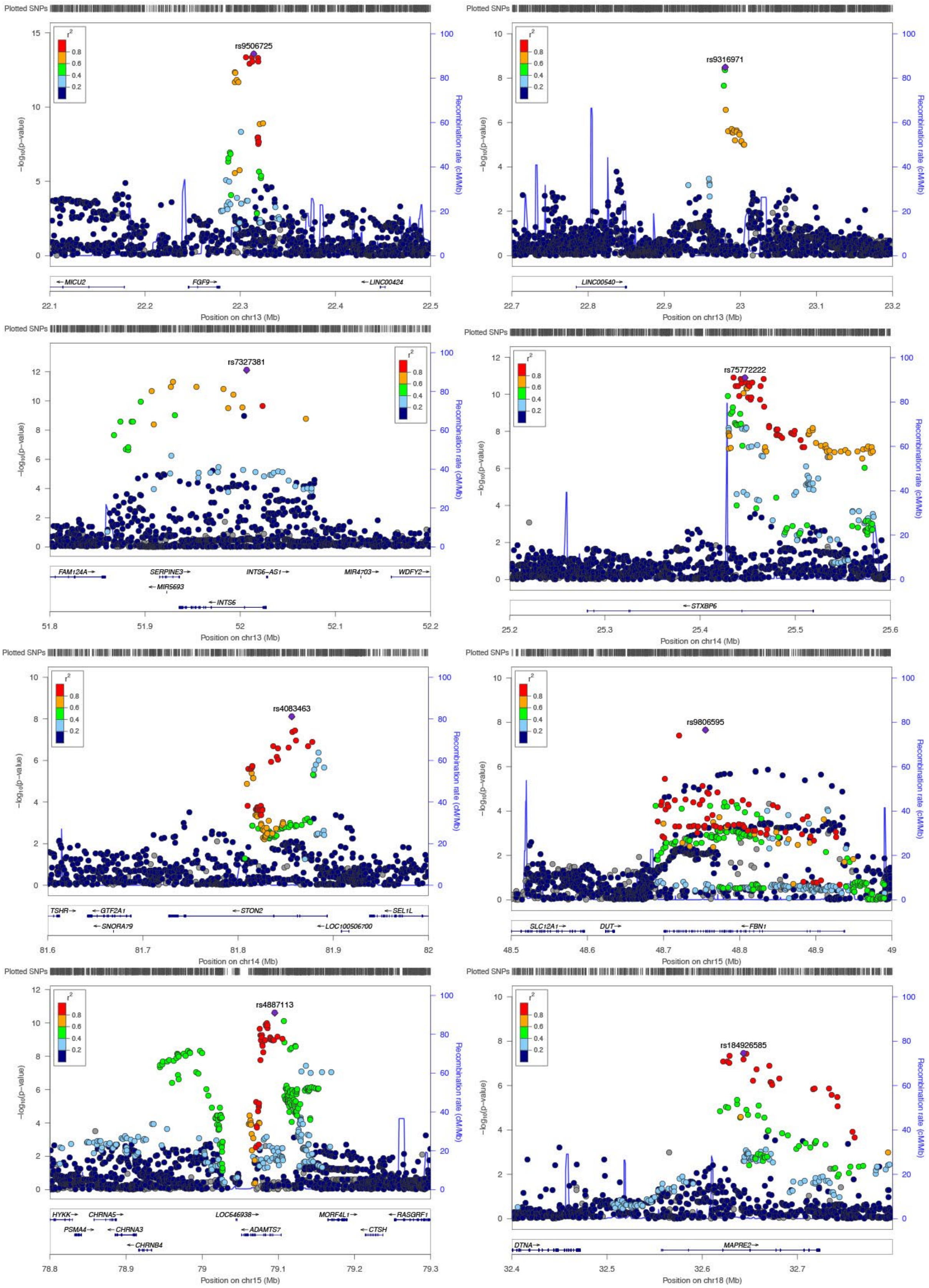
Plotted SNPs
rs9506725
r^2
0.8
0.6
0.4
0.2
$-\log_{10}$(p-value)
Recombination rate (cM/Mb)
MICU2
FGF9
LINC00424
Position on chr13 (Mb)
rs9316971
LINC00540
Position on chr13 (Mb)
rs7327381
FAM124A
SERPINE3
INTS6-AS1
MIR4703
WDFY2
MIR5693
INTS6
Position on chr13 (Mb)
rs75772222
STXBP6
Position on chr14 (Mb)
rs4083463
TSHR
GTF2A1
STON2
SEL1L
SNORA79
LOC100506700
Position on chr14 (Mb)
rs9806595
SLC12A1
DUT
FBN1
Position on chr15 (Mb)
rs4887113
HYKK
CHRNA5
LOC646938
MORF4L1
RASGRF1
PSMA4
CHRNA3
ADAMTS7
CTSH
CHRNB4
Position on chr15 (Mb)
rs184926585
DTNA
MAPRE2
Position on chr18 (Mb)

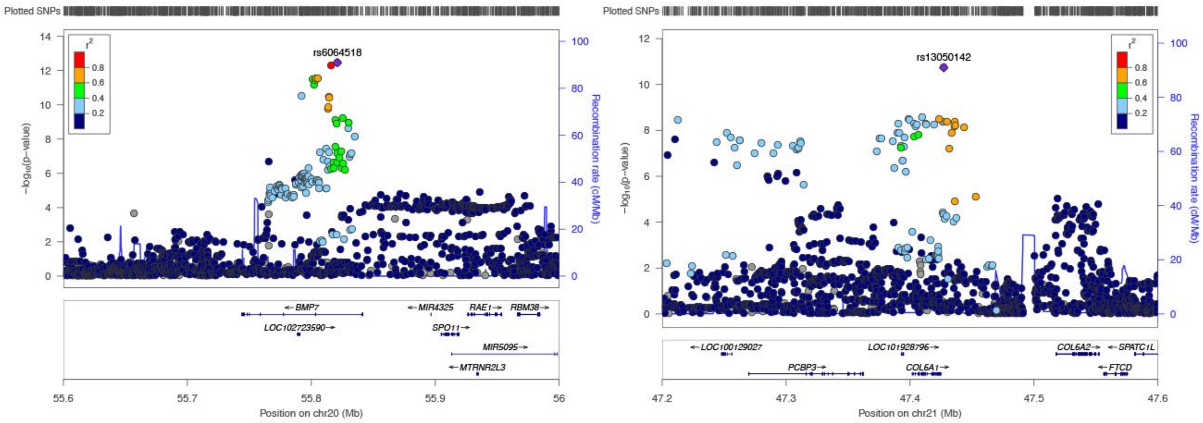

Plotted SNPs
rs6064518
r^2
0.8
0.6
0.4
0.2
$-\log_{10}$(p-value)
Recombination rate (cM/Mb)
BMP7
LOC102723590
MIR4325
RAE1
RBM38
SPO11
MIR5095
MTRNR2L3
Position on chr20 (Mb)
55.6
55.7
55.8
55.9
56
rs13050142
LOC100129027
LOC101928796
COL6A2
SPATC1L
PCBP3
COL6A1
FTCD
Position on chr21 (Mb)
47.2
47.3
47.4
47.5
47.6

Supplementary Figure 5. Overlap of genes associated with corneal curvature, axial length and spherical equivalent. Genes in black are CC-associated genes identified in the current study; Genes in blue are genes for axial length identified from previous papers [23,27].

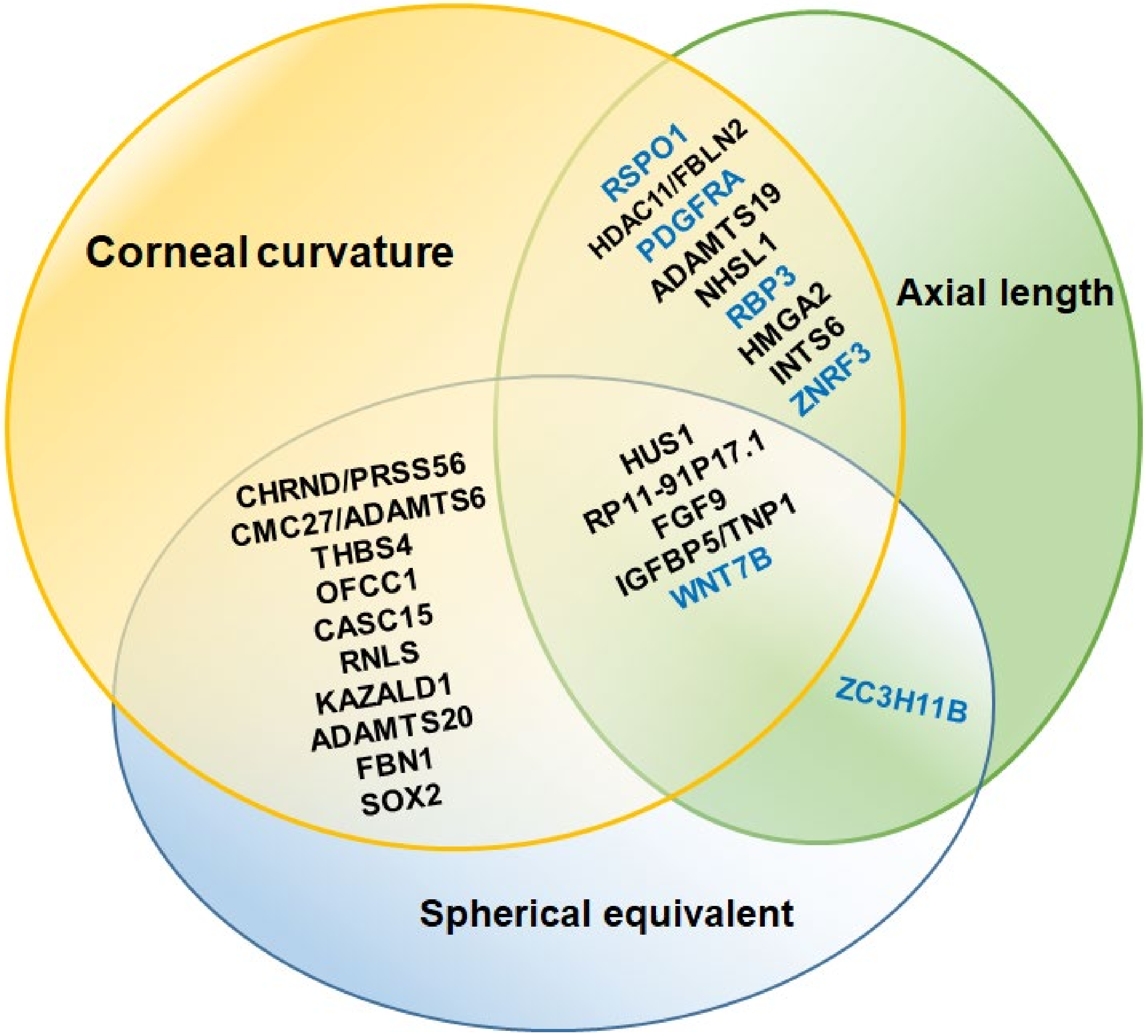

Supplementary Figure 6 Network shows functional annotation of genes at top loci

This figure shows the top network (score=38, IPA computes a score for each network according to the fit of that network to the user-defined set of Focus Genes) generated by IPA network analysis. 'IPA overlay function' was applied to show the significant pathways (after FDR correction). All symbols were correlated. Figure shows the correlation between connective tissue disorder with symbols involved in the network. IPA: https://www.qiagenbioinformatics.com/products/ingenuity-pathway-analysis/.

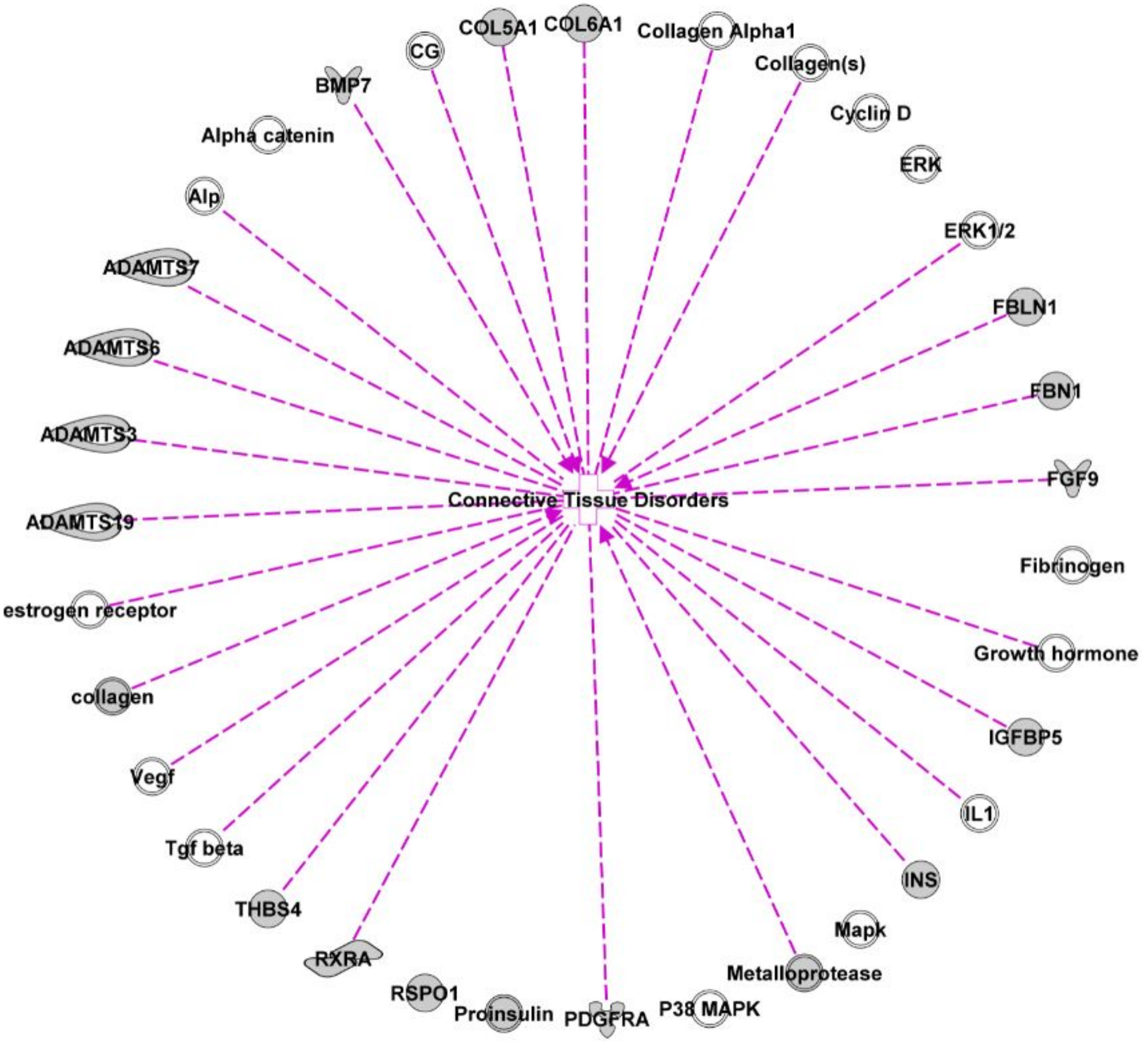

Supplementary Table 1. Study cohorts and summary of corneal curvature measures (total N=44,042)

Study		N	Male, %	Age (SD), years	Mean (SD), mm	Method of measurement
**European ancestry**		**29580**				
ALSPAC		2113	46.7	15.5 (0.3)	7.82 (0.26)	IOLmaster
BATS						
	10 ≤ age < 25	992	46.3	19.1 (3.2)	7.67 (0.25)	Humphrey-598 Automatic Refractor/Keratometer
	age ≥ 25	157	43.3	26.5 (2.4)	7.64 (0.27)	Humphrey-598 Automatic Refractor/Keratometer
BMES		1269	42.7	67.1 (9.2)	7.77 (0.61)	IOLmaster
Croatia-Korcula		849	35.8	56.7 (13.4)	7.78 (0.31)	Eschosan US-1800
Croatia-Split		762	39.4	52.1(13.0)	7.76 (0.27)	Eschosan US-1800
Croatia-Vis		568	40	56.4 (13.4)	7.73 (0.26)	Eschosan US-1800
ERF		2530	45	50.2 (12.8)	7.72 (0.27)	Topcon RM-A2000 autorefractor
EPIC-Norfolk		927	42.5	68.9 (7.5)	7.74 (0.25)	IOLmaster v4
FITSA		116	0	67.9 (3.1)	7.55 (0.22)	IOLmaster
GHS1		2867	51.1	55.80 (10.90)	7.76 (0.26)	Lenstar LS 900
GHS2		1013	49.4	55.10 (10.90)	7.76 (0.25)	Lenstar LS 900
Generation R		2073	50	6.09 (0.40)	7.82 (0.26)	IOLmaster
OGP-Talana						
	10 ≤ age < 25	83	43.4	16.6 (3.9)	7.75 (0.26)	IOLmaster
	age ≥ 25	428	36.4	51.6 (15.3)	7.64 (0.26)	IOLmaster
RAINE		935	49.9	20.0 (0.4)	7.73 (0.25)	IOLmaster
RS1		5744	41	68.8 (8.8)	7.67 (0.25)	Topcon RM-A2000 autorefractor
RS2		2547	46	64.2 (7.5)	7.70 (0.25)	Topcon RM-A2000 autorefractor
RS3		2960	44	56.9 (6.5)	7.75 (0.26)	Topcon RM-A2000 autorefractor
TEST						
	age < 10	176	47.7	7.49 (1.22)	7.77 (0.27)	IOLmaster

10 ≤ age < 25	208	36.1	15.6 (4.2)	7.71 (0.27)	IOLmaster
age ≥ 25	263	34.2	46.7 (13.2)	7.66 (0.25)	IOLmaster
**Asian ancestry**	**14462**				
BES	576	34.1	62.2 (8.6)	7.59 (0.26)	Lenstar LS 900
GTES	1052	48.5	15.5(2.8)	7.75(0.26)	Topcon KR8800 autorefraction
Hong Kong Study					
10 ≤ age < 25	76	40.8	21.8(2.1)	7.81 (0.22)	Canon RK-5 Auto Ref-keratometer
age ≥ 25	110	37.3	34.7 (6.5)	7.74 (0.22)	Canon RK-5 Auto Ref-keratometer
Nagahama Study	2747	34.1	52.1 (13.8)	7.68 (0.26)	ARK-530A (Nidek)
SCES	1731	51.6	57.6 (9.0)	7.66 (0.25)	IOLmaster
SCORM	912	51.9	10.8 (0.8)	7.76 (0.24)	Auto Ref-Keratometer
SiMES	2138	49.3	57.6 (10.7)	7.66 (0.25)	IOLMaster
SINDI	2124	51.4	55.9 (8.8)	7.62 (0.27)	IOLMaster
SP2-550	324	24.1	49.7 (12.7)	7.79 (0.25)	Auto Ref-Keratometer
SP2-1M	846	63.8	46.8 (10.1)	7.75 (0.28)	Auto Ref-Keratometer
SP2-610	1086	22.9	48.6 (11.3)	7.70 (0.28)	Auto Ref-Keratometer
STARS	740	51.1	38.9 (5.2)	7.68 (0.28)	IOLMaster

ALPACS, Avon Longitudinal Study of Parents and Children; BATS, Brisbane Adolescent Twins Study; BMES, Blue Mountains Eye Study; ERF4, Erasmus Rucphen Family Study 4; EPIC, European Prospective Investigation of Cancer; FITSA, Finnish Twin Study on Ageing; GHS, Gutenberg Health Study; OGP, Ogliastra Genetic Park; RAINE, RAINE eye health study; RS, Rotterdam Study; TEST, Twins Eye Study in Tasmania; BES, Beijing Eye Study; GTES, Guangzhou Twins Eye Study; SCES, Singapore Chinese Eye Study; SCORM, Singapore Cohort Study of the Risk Factors for Myopia; SiMES, Singapore Malay Eye Study; SINDI, Singapore Indian Eye Study; SP2, Singapore Prospective Study; STARS, Strabismus, Amblyopia, & Refractive Error Study of Preschool Children.

Supplementary Table 2. Genotyping and imputation information for the included cohorts

Study	Genotyping method/platform	Imputation software	Reference population	No of SNPs	$Lambda_{gc}$
**EUROPEANS**					
ALSPAC Children	Illumina Infinium II HumanHap550	MACH v.1.0.16	1000 Genome Reference panels	8219160	0.977
BATS (10 < age ≤ 25)	Illumina 660 Quad	MACH v2	1000 Genome Reference panels	7708087	0.994
BATS (25 < age)	Illumina 660 Quad	MACH v2	1000 Genome Reference panels	7708087	0.988
BMES	Illumina Human 670-Quad v1	MACH v1.1.16	1000 Genome Reference panels	4008218	0.872
Croatia-Korcula	Illumina 370CNV-Quad v1	MACH v1.0.15	1000 Genome Reference panels	8027707	0.992
Croatia-Split	Illumina 370CNV-Quad v3	MACH v1.0.15	1000 Genome Reference panels	4503699	1.02
Croatia-Vis	HumanHap 300v1	MACH v1.0.15	1000 Genome Reference panels	7849213	1.006
ERF4	Illumina 6k, Illumina 318K, Illumina 370K, and Affymetrix 250K	MACH v1.0.15	1000 Genome Reference panels	6318051	1.085
EPIC-Norfolk	Affymetrix GeneChip Human Mapping 500K	IMPUTE2	1000 Genome Reference panels	8697280	1.001
FITSA	Illumina HumanCoreExome	IMPUTE2	1000 Genome Reference panels	8936601	1.013
GHS1	Affymetrix Human SNP 6.0	Impute (v2.1.0)	1000 Genome Reference panels	7344430	1.024
GHS2	Affymetrix Human SNP 6.0	Impute (v2.1.0)	1000 Genome Reference panels	7350794	1.027
Generation R	Illumina 610/660 Quad	MACH v2	1000 Genome Reference panels	8179580	1.039
OGP-Talana (10 < age ≤ 25)	500K Affymetrix GeneChip	MACH v1.0.16	1000 Genome Reference panels	7191761	1.031
OGP-Talana (25 < age)	500K Affymetrix GeneChip	MACH v1.0.16	1000 Genome Reference panels	7859715	1.002
RAINE	Illumina 610/660 Quad	MACH v2	1000 Genome Reference panels	6250269	1.005
RS1	Illumina HumanHap550	MACH v1.0.15	1000 Genome Reference panels	8304111	0.977
RS2	Illumina HumanHap550 Duo, Human610-Quad	MACH v1.0.16	1000 Genome Reference panels	8278354	1.019
RS3	Human 610 Quad Arrays Illumina	MACH v1.0.17	1000 Genome Reference panels	8339676	1.054
TEST (age ≤ 10)	Illumina 660 Quad	MACH v2	1000 Genome Reference panels	7708087	1.004
TEST(10 < age ≤ 25)	Illumina 660 Quad	MACH v2	1000 Genome Reference panels	7708087	1.026

TEST (25 < age)	Illumina 660 Quad	MACH v2	1000 Genome Reference panels	7708087	1.073
**ASIANS**			1000 Genome Reference panels		
BES	Illumin 610	IMPUTE v0.5.0	1000 Genome Reference panels	7084108	1.021
GTES	Affymetrix Titan platform with Asian Axiom chip	Impute2	1000 Genome Reference panels	6500104	1.012
Hong Kong Study (10 < age ≤ 25)	Illumina HumanCNV370-Quadv3	IMPUTE v2.3.0	1000 Genome Reference panels	7419383	0.996
Hong Kong Study (25 < age)	Illumina HumanCNV370-Quadv3	IMPUTE v2.3.0	1000 Genome Reference panels	7462396	1.011
Nagahama Study	HumanHap610KQuad, HumanOmni2.5M, HumanExome	Minimac	1000 Genome Reference panels	5911014	1.016
SCES	Illumin610 Quad	Minimac	1000 Genome Reference panels	7030375	1.013
SCORM	HumanHap 550, 550Duo	Minimac	1000 Genome Reference panels	6983145	1.009
SiMES	Illumin610 Quad	Minimac	1000 Genome Reference panels	7246124	1.012
SINDI	Illumin610 Quad	Minimac	1000 Genome Reference panels	7861976	0.988
SP2-550	Illumin550	Minimac	1000 Genome Reference panels	6960896	0.977
SP2-1M	Illumin 1 million	Minimac	1000 Genome Reference panels	7313116	1.002
SP2-610	Illumin610 Quad	Minimac	1000 Genome Reference panels	7067357	1.013
STARS	Illumin610 Quad	Minimac	1000 Genome Reference panels	7044441	1.000

ALPACS, Avon Longitudinal Study of Parents and Children; BATS, Brisbane Adolescent Twins Study; BMES, Blue Mountains Eye Study; ERF4, Erasmus Rucphen Family Study 4; EPIC, European Prospective Investigation of Cancer; FITSA, Finnish Twin Study on Ageing; GHS, Gutenberg Health Study; OGP, Ogliastra Genetic Park; RAINE, RAINE eye health study; RS, Rotterdam Study; TEST, Twins Eye Study in Tasmania; BES, Beijing Eye Study; GTES, Guangzhou Twins Eye Study; SCES, Singapore Chinese Eye Study; SCORM, Singapore Cohort Study of the Risk Factors for Myopia; SiMES, Singapore Malay Eye Study; SINDI, Singapore Indian Eye Study; SP2, Singapore Prospective Study; STARS, Strabismus, Amblyopia, & Refractive Error Study of Preschool Children.

Supplementary Table 3. Association for corneal curvature at proxy SNPs for those monomorphic SNPs in Table1

					European				Asian				All			
A) Lead SNPs are monomorphic in Asians and corresponding proxy SNPs nearby																
**Lead SNP**	CHR	POS	**GENE**	A1/A2	EAF	β	s.e	*P*	EAF	β	s.e	*P*	β	s.e	*P*	Het_P
rs2630445	3	13554886	HDAC11/FBLN2	T/G	0.10	0.030	0.004	1.17E-15	0.00				0.030	0.004	1.17E-15	0.58
rs7708378	5	129088784	ADAMTS19/CHSY3	T/G	0.07	0.028	0.004	2.49E-09	0.00				0.028	0.004	2.49E-09	0.41
rs9506725	13	22314146	FGF9	T/C	0.64	0.019	0.002	3.07E-14	1.00				0.019	0.002	3.07E-14	0.37
**Proxy SNP**	CHR	POS	**GENE**	A1/A2	EAF	β	s.e	P	EAF	B	s.e	P	B	s.e	P	Het_P
rs2655225	3	13551420	HDAC11/FBLN2	A/ G	0.86	-0.026	0.003	5.69E-13	0.48	-0.010	0.003	3.34E-03	-0.018	0.002	1.29E-12	0.021
rs11746536	5	129095006	*ADAMTS19*	C/ G	0.64	-0.012	0.002	4.07E-06	0.53	-0.011	0.003	2.67E-04	-0.012	0.002	6.90E-09	0.316
rs507446	13	22318497	*FGF9*	T/ C	0.62	0.016	0.002	1.99E-10	0.80	0.002	0.004	7.22E-01	0.012	0.002	1.11E-08	0.148
rs2655225, rs2630445		r2=0.6207														
rs7708378, rs11746536		r2=0.1079														
B) Lead SNPs are monomorphic in Europeans and corresponding proxy SNPs nearby																
**Lead SNP**	CHR	POS	**GENE**	A1/A2	EAF	β	s.e	P	EAF	B	s.e	P	B	s.e	P	Het_P
rs60078183	1	47857307	CMPK1	A/G	0.00				0.21	-0.037	0.005	1.37E-15	-0.037	0.005	9.74E-16	0.69
	10	48388228	RBP3	T/C	0.00				0.04	0.071	0.010	9.83E-13	0.071	0.010	9.83E-13	0.66
**Proxy SNP**																
rs11211514	1	47787954	CMPK1	T/G	0.02	0.010	0.011	0.4109	0.16	-0.030	0.004	3.83E-14	-0.026	0.004	5.91E-11	0.34
rs34654656	10	48400296	*RBP3*	T/ C	0.98	0.010	0.017	5.80E-01	0.95	-0.062	0.009	2.56E-12	-0.047	0.008	1.29E-08	0.053

Supplementary Table 4. Additional significant hits when Europeans and Asians analysed separately

A. Additional genome-wide significant hits ($P < 5 \times 10^{-8}$) when Europeans and Asians analysed separately

						CREAM-Europeans					CREAM-Asians					CREAM-ALL			
SNP	chr	pos	Gene	A1	A2	Freq	β	s.e	P	Het-P	Freq	β	s.e	P	Het-P	β	s.e	P	Het-P
Asains																			
rs2240776	10	119307524	EMX2/EMX2OS	a	t	0.627	0.004	0.002	0.120	0.690	0.533	0.017	0.003	2.45E-08	0.524	0.009	0.002	6.09E-06	0.207
rs7672919	4	17797455	NCAPG	t	g	0.139	-0.006	0.003	0.072	0.241	0.328	-0.018	0.003	3.90E-08	0.863	-0.012	0.002	6.23E-07	0.257
Europeans																			
None																			

B. Additional top hits when Europeans and Asians analysed separately ($P < 1 \times 10^{-7}$)

								CREAM-Europeans					CREAM-Asians				CREAM-ALL		
SNP	chr	pos	Gene	A1	A2	Freq	beta	se	P	Het-P	Freq	beta	se	P	Het-P	beta	se	P	Het-P
Asians																			
rs12593707	15	67088350	SMAD6	t	c	0.015	0.003	0.013	0.816	0.627	0.209	0.023	0.004	8.46E-08	0.473	0.021	0.004	6.67E-07	0.554
Europeans																			
rs2101976	18	11256751	PIEZO2	t	c	0.757	0.015	0.0026	7.32E-08	0.4	0.956	0.0013	0.012	9.12E-01	0.350	0.014	0.003	1.18E-07	0.36

Supplementary Table 5. CREAM cohorts to test association between identified variants and axial length

	Study	N
Asian Cohorts	SCES	1697
	SIMES	2139
	SINDI	2080
	GTES	1055
	Total	6971
European Cohorts	ALSPAC	2254
	Raine	1026
	OGP	86
	RS1	1170
	RS2	1299
	RS3	2612
	ERF	2404
	Total	10851

Supplementary Table 6. Pleiotropic effect ratio estimation for CC-variants

	β_{AL} to β_{CC}	I^2 (%)	P
Group A	2.92 (2.37, 3,48)	0	<0.0001
Group B	1.66 (1.16, 2.16)	40.7	<0.0001
Group C	1.19 (0.78, 1.62)	0	<0.0001
Group A vs Group B			7.56 x 10-4
Group A vs Group C			1.68 X 10-6
Group B vs Group C			0.407

Supplementary Table 7. Significant Loci for corneal curvature identified through the gene-based tests

				Europeans			Asians			All
Chr	Gene	Start position	Stop position	Best SNP	Best SNP P	Gene-base P	Best SNP	Best SNP P	Gene-base P	Gene-based P
1	*ANKRD65*	1303799	1406824	rs2649590	1.15E-06	3.00E-06	rs9661288	1.26E-03	1.69E-02	9.00E-07
1	*PEAR1*	156813522	156936226	rs703156	1.60E-05	1.00E-05	rs41273221	1.17E-03	9.13E-03	1.57E-06
2	*ASB1*	239285625	239410891	rs61332075	1.17E-07	1.00E-06	rs111233411	1.22E-03	1.33E-02	2.54E-07
6	*GMDS*	1574034	2295868	rs6912735	5.50E-06	1.00E-06	rs9503059	3.33E-05	1.15E-03	2.48E-08
10	*EMX2/EMX2OS**	119193803	119354579	rs1981661	1.13E-03	2.32E-03	rs2240776	1.45E-08	1.00E-06	4.84E-08
20	*HM13-AS1*	30105509	30211066	rs6088749	8.82E-03	1.13E-02	rs6059952	4.50E-02	1.00E-06	2.18E-07

* EMX2/EMX2OS was identified as Asian-speciifc locus from SNP-based analyses; see Supplementary Table 6.
Gene with smallest gene-based p-value ta each locus is listed in the table
Gene-based analysis was performed for each gene +/-50kb flanking, using the VEGAS, with p-value at 2.09E-06 for gene-set significance.

Study Description

EUROPEAN ETHNICITY STUDIES

Avon Longitudinal Study of Parents and Children (ALSPAC)

Details of ALSPAC cohorts have been published previously[1; 2]. The research adhered to the tenets of the Declaration of Helsinki. Ethical approval for the study was obtained from the ALSPAC Law and Ethics committee and the Local Research Ethics Committees. Pregnant women resident in Avon, UK with expected dates of delivery 1st April 1991 to 31st December 1992 were invited to take part in the study. The initial number of pregnancies enrolled was 14,541 (for these at least one questionnaire has been returned or a "Children in Focus" clinic had been attended by 19/07/99). Of these initial pregnancies, there was a total of 14,676 foetuses, resulting in 14,062 live births and 13,988 children who were alive at 1 year of age. Data collection has been via various methods including self-completion questionnaires sent to the mother, to her partner and after age 5 to the child; direct assessments and interviews in a research clinic. Please note that the study website contains details of all the data that is available through a fully searchable data dictionary and variable search tool" and reference the following webpage: http://www.bristol.ac.uk/alspac/researchers/our-data/. As well as investigating the health and well-being of the children in the birth cohort, the health of the mothers is also an important area of investigation. For mothers, DNA was extracted from blood samples collected as part of routine antenatal care, during attendance at ALSPAC research clinics, or from immortalized lymphoblastoid cell lines, for a total of 10,321 of the mothers. Non-cycloplegic autorefraction (Canon R50 instrument) was performed opportunistically when mothers accompanied their child to a research clinic visit, and/or by a researcher visiting their optician to obtain their spectacle prescription. Non-cycloplegic autorefraction data was used in preference to subjective refraction data when available. DNA samples were available for 11,343 children, prepared from either blood samples or lymphoblastoid-transformed cell lines. Non-cycloplegic autorefraction (Canon R50 instrument) was performed during attendance at an ALSPAC research clinic visit when the children were approximately 15 years old. Genotyping was performed using Illumina 660 W-quad (mothers) or Illumina HumanHap 550 (children) bead arrays. Samples that did not cluster with HapMap CEU individuals on IBS plots, with excessive missingness (>5%), minimal or excessive autosomal heterozygosity, cryptic relatedness (>10% IBD) or with a sex-mismatch were excluded. SNPs with call rate <95%, minor allele frequency <1%, or Hardy-Weinberg P value < 10^{-7} were excluded. Genotypes were available for 8340 mothers and 8365 children. Imputation was carried out separately for Mothers and Children. For mothers, individual chromosomes were pre-phased with ShapeIt v2 using the b37 genetic map, and imputation was performed with minimac-omp using the GIANT phase1 release v3 (2010-11-23) 1000 Genomes reference panel. For children, phasing was carried out using MACH and imputation with minimac, against the same reference panel.

BATS

The Brisbane Adolescent Twins Study (BATS) is a part of the Australian Twin Eye Study, which was described elsewhere [3]. Ethical approval was obtained from the Royal Victorian Eye and Ear Hospital, the University of Tasmania, the Australian Twin Registry and the Queensland Institute of Medical Research. In all subjects post-cycloplegic (following instillation of tropicamide 1%) refraction for both eyes was measured using a Humphrey-598 automatic refractor (Carl Zeiss Meditec, Inc., Miami, Florida, USA). These measurements were used to determine the spherical equivalence trait analysed here. Education data in BATS were collected as part of the 19UP study, through either telephone interviews or online questionnaires.

DNA was extracted from blood leucocytes according to standard procedures. The Australian cohorts were genotyped on the Illumina Human Hap610 Quad array. SNPs with a genotype success rate of 0.95 or above was required for inclusion of the SNP into further steps of the analysis. Only SNPs in Hardy-Weinberg equilibrium were processed: the HWE inclusion threshold was P>10x10-6. The minimum minor allele frequency required for inclusion of individual SNPs was 0.01. Ancestral outliers were defined as having the first two principal components more than six standard deviations from the mean values of HapMap European samples, and therefore were subsequently excluded from the analyses. Imputation was performed against version 3 of the November 23, 2010 version of the publicly released 1000 Genomes Project genotyping, using MACH

(http://www.sph.umich.edu/csg/abecasis/MACH/) for phasing and minimac for imputation (http://genome.sph.umich.edu/wiki/Minimac).

Blue Mountains Eye Study (BMES)
The Blue Mountains Eye Study (BMES) is a population-based cohort of a predominantly white population in west of Sydney, Australia. At baseline (1992-94), 3,654 permanent residents aged 49 years or older participated (participation rate of 82.4%[9]. During 1997-99 (BMES II A), 2,335 participants (75.1% of survivors) returned for examinations after 5 years. During 1999-2000, 1,174 (85.2%) new participants took part in an Extension Study of the BMES (BMES IIB). BMES cross-section II thus includes BMES IIA (66.5%) and BMES IIB (33.5%) participants (n=3,509)[4]. From the BMES cross section II who had blood samples collected, DNA was extracted for 3,189 (90.1 %) participants. Over 98% of BMES participants were European ancestry. All BMES examinations were approved by the Human Ethics Committees of the Western Sydney Area Health Service and University of Sydney. Signed informed consent was obtained from participants at each examination. Participants of the BMES cross section II who had DNA available in early 2009 (n=2983) were genotyped using the Illumina Human 670-Quadv1 custom genotyping array at the Wellcome Trust Sanger Institute, Cambridge as part of WTCCC2, and 2,761 had genotyping data available. Following exclusion through GWAS and DNA quality control and phenotype exclusion criteria resulted in genotyping data being available for 1,896 individuals. Imputation was performed to HapMap (NCBI Build 36.1) using MACH (V 1.0.16; autosomes only). Imputed SNPs were excluded from the analysis when failing one or more of the following QC filters: 1) prop info ≥ 0.5 (a software-specific statistic from IMPUTE); 2) Hardy-Weinberg P-value < 1×10^{-6}.

CROATIA-Korčula Study
The CROATIA-Korčula study, Croatia, is a population-based, cross-sectional study that includes a total of 969 adult examinees, aged 18-98 (mean=56.3), from the Dalmatian island of *Korčula* and most (N=930) underwent a complete eye examination [5]. The study received approval from relevant ethics committees in Scotland and Croatia and followed the tenets of the Declaration of Helsinki. Non-cycloplegic autorefraction was measured on each eye using a NIDEK Ark30 hand-held autorefractometer. Measures on eyes with a history of trauma, intra-ocular surgery, LASIK operations or keratoconus were removed. Analysis was performed as per analysis plan, excluding individuals with a cylinder power >= 5D in either eye and individuals with difference in cylinder power between right and left eyes beyond 4 standard deviations from the mean, and for over 25 year-old only as there were too few individuals in this study who were under 25 years of age. Genotypes were generated using a dense Illumina SNP arrays, Illumina CNV370v1 and CNV370-Quadv3, following the manufacturer's standard recommendations. Genotypes were determined using the Illumina BeadStudio software. Samples with a call rate below 97 % , potentially mixed samples with excess autosomal heterozygosity or gender discrepancy (based on the sex chromosomes genotypes), and ethnic outliers (based on principal components analysis of genotypic data), were excluded from the analysis using the quality control algorithm implemented in the R package GenABEL. After exclusion of SNP with MAF < 0.01, call rate < 98% and HWE deviation $p < 10^{-6}$, samples were pre-phased using shapeit v2[6]. Imputation was carried out using impute v2[7] and the 1,000 genomes All ancestries phase1 integrated v3 reference panel. The impute2mach GENABEL function was used to convert the impute2 outputs to the MACH format that is used in the ABEL suite (http://www.genabel.org/packages) and the regression analyses adjusted for age and sex on SNP allele dose using the MixABEL package. The variance covariance matrix used in MixABEL to account for relatedness between individuals was generated using the polygenic functions of the GenABEL package.

CROATIA-Split Study
The CROATIA-Split study, Croatia, is a population-based, cross-sectional study in the Dalmatian City of Split that includes 1000 examinees aged 18-95. The study received approval from relevant ethics committees in Scotland and Croatia and followed the tenets of the Declaration of Helsinki.
Individuals were genotyped with either the 370CNV-Quadv3 (n=500) or the Illumina OmniExpress Exome-8v1_A beadchips (n=500). Alleles were called in BeadStudio/GenomeStudio using Illumina cluster files. Subjects were excluded if they fulfilled any of the following criteria: genotypic call rate <97%, mismatch between reported

and genotypic sex, unexpectedly low genomic sharing with first degree relatives, excess autosomal heterozygosity, or outliers identified by IBS clustering analysis. We excluded SNPs on the basis of minor allele frequency (<0.01/monomorphism), HWE (P<10^-6), call rate (<97%). The samples genotyped with the denser array (Illumina OmniExpress Exome) were first prephased and imputed as described for the CROATIA-Korcula study and the output of this imputation used as a secondary panel to complement the 1,000 genomes All ancestries phase1 integrated v3 reference panel for the imputation of the samples genotyped on the less dense array. Imputations for the two halves of the study were then combined to form a combined panel of ~37.5m SNPs. Genome-wide scan for association was performed as described in the CROATIA-Korcula Study.

CROATIA-Vis Study

The CROATIA-Vis study, Croatia, is a population-based, cross-sectional study including adult participants, aged 18–93 years (mean = 56), from the Dalmatian island of Vis, a subset of which (N=640) underwent a complete eye examination in summer 2007 and provided their ophthalmologic history [5]. The study received approval from relevant ethics committees in Scotland and Croatia and followed the tenets of the Declaration of Helsinki. Genotypes were generated using a dense Illumina SNP array, HumanHap 300v1, following the manufacturer's standard recommendations. Genotypes were determined using the Illumina BeadStudio software. Samples with a call rate below 97 % , potentially mixed samples with excess autosomal heterozygosity or gender discrepancy (based on the sex chromosomes genotypes), and ethnic outliers (based on principal components analysis of genotypic data), were excluded from the analysis using the quality control algorithm implemented in the R package GenABEL. Imputation of allele dosage to the 1,000 genomes all ancestries phase1 integrated v3 reference panel and genome-wide scan for association were performed as described in the CROATIA-Korcula Study.

EPIC-Norfolk Eye Study (EPIC)

The European Prospective Investigation into Cancer (EPIC) study is a pan-European prospective cohort study designed to investigate the aetiology of major chronic diseases[8]. EPIC-Norfolk , one of the UK arms of EPIC, recruited and examined 25,639 participants aged 40-79 years between 1993 and 1997 for the baseline examination[9]. Recruitment was via general practices in the city of Norwich and the surrounding small towns and rural areas, and methods have been described in detail previously [10]. Since virtually all residents in the UK are registered with a general practitioner through the National Health Service, general practice lists serve as population registers. Ophthalmic assessment formed part of the third health examination and this has been termed the EPIC-Norfolk Eye Study[11]. In total, 8,623 participants were seen for the ophthalmic examination, between 2004 and 2011. Refractive error was measured using a Humphrey Auto-Refractor 500 (Humphrey Instruments, San Leandro, California, USA). Educational level was recorded and classified into four groups according to the highest qualification achieved (Less than O level / O Level / A level / Degree). For the purposes of the current study, educational attainment was dichotomised into lower (Less than O level / O Level) or higher (A level / Degree). Genotyping was undertaken using the Affymetrix GeneChip Human Mapping 500K Array Set. Data were pre-phased with SHAPEIT version 2 and imputed to the March 2012 build of the 1000 Genomes project using IMPUTE version 2.2.2. The EPIC-Norfolk Eye Study was carried out following the principles of the Declaration of Helsinki and the Research Governance Framework for Health and Social Care. The study was approved by the Norfolk Local Research Ethics Committee (05/Q0101/191) and East Norfolk & Waveney NHS Research Governance Committee (2005EC07L). All participants gave written, informed consent.

Erasmus Rucphen Family Study (ERF)

The Erasmus Rucphen Family (ERF) Study is a family-based cohort in a genetically isolated population in the southwest of the Netherlands with over 3,000 participants aged between 18 and 86 years. Cross-sectional examination took place between 2002 and 2005. The rationale and study design of this study have been described elsewhere [12; 13]. Cross-sectional examination took place between 2002 and 2005, including a non-dilated automated measurement of refractive error using a Topcon RM-A2000 autorefractor. All measurements in these studies were conducted after the Medical Ethics Committee of the Erasmus University had approved the study

protocols and all participants had given a written informed consent in accordance with the Declaration of Helsinki.

DNA was genotyped on one of four different platforms (Illumina 6k, Illumina 318K, Illumina 370K and Affymetrix 250K). Samples with low call rate (<97.5%), with excess autosomal heterozygosity (>0.336), or with sex-mismatch were excluded, as were outliers identified by the identity-by-state clustering analysis (outliers were defined as being >3 s.d. from population mean or having identity-by-state probabilities >97%). A set of genotyped input SNPs with call rate >98%, with minor allele frequency >0.01, and with Hardy-Weinberg P value $>10^{-6}$ was used for imputation. We used Minimac to impute to 1000G (phase 1, March 2012). For each imputed SNP, a reliability of imputation was estimated as the ratio of the empirically observed dosage variance to the expected binomial dosage variance (O/E ratio). GWAS analyses were performed using the MixABEL package and adjusted for family structure in the first step of two-staged modelling.

Finnish Twin Study on Aging (FITSA)

Finnish Twin Study on Aging (FITSA) [14] is a study of genetic and environmental effects on the disablement process in older female twins. The FITSA participants were 103 MZ and 114 DZ Finnish twin pairs (424 individuals, all Caucasian women) aged 63-76 years who took part in multiple laboratory examination in 2000, 2003 and responded in questionnaires in 2011. Before the examinations, the subjects provided a written informed consent according to the Declaration of Helsinki. The study protocol was approved by the ethics committee of the Central Hospital District of Central Finland.

DNA was extracted from EDTA-anticoagulated whole blood according to standard procedures. The genotyping was carried out with Illumina HumanCoreExome chip. The genotyping quality control thresholds included minor allele frequency >0.01, success rate by marker >0.95, success rate by individual >0.95, and HWE P>0.000001. The imputation was performed with SHAPEIT2 and IMPUTE2 with 1000 Genomes haplotypes reference panel (Phase I integrated variant set release in NCBI build 37 (hg19) coordinates).

Gutenberg Health Study (GHS1, GHS2)

The Gutenberg Health Study (GHS) is a population-based, prospective, observational cohort study in the Rhine-Main Region in midwestern Germany with a total of 15,010 participants and follow-up after five years. The study sample is recruited from subjects aged between 35 and 74 years at the time of the exam. The sample was drawn randomly from local governmental registry offices and stratified by gender, residence (urban and rural) and decade of age. Exclusion criteria were insufficient knowledge of the German language to understand explanations and instructions, and physical or psychic inability to participate in the examinations in the study center. Individuals were invited for a 5-hour baseline-examination to the study center where clinical examinations and collection of blood samples were performed. The interdisciplinary study design comprises an ophthalmological examination, general and especially cardiovascular examinations, psychosomatic evaluation, laboratory tests, and biobanking for proteomic and genetic analyses. All participants underwent an ophthalmological investigation of 25 minutes' duration taking place between 11:00 a.m. and 8:00 p.m. This examination was based on standard operating procedures and included a medical history of eye diseases, autorefraction and visual acuity testing (Humphrey® Automated Refractor/Keratometer (HARK) 599™, Carl Zeiss Meditec AG, Jena, Germany), visual field screening using frequency doubling technology (Humphrey® Matrix Perimeter, Carl Zeiss Meditec AG, Jena, Germany), central corneal thickness and keratometry measurement (Scheimpflug imaging with the Pachycam™, Oculus, Wetzlar, Germany), IOP measurement with a non-contact tonometer (Nidek NT-2000™, Nidek Co., Japan), slitlamp biomicroscopy with undilated pupils (Haag-Streit BM 900®, Bern, Switzerland) and non-mydriatic fundus photography (Visucam PRO NM,™, Carl Zeiss Meditec AG, Jena, Germany), all administered by an ophthalmologist. The study was approved by the Medical Ethics Committee of the University Medical Center Mainz and by the local and federal data safety commissioners. According to the tenets of the Declaration of Helsinki, written informed consent was obtained from all participants prior to entering the study.

Within GHS, DNA was extracted from buffy-coats from EDTA blood samples as described in Zeller *et al.*[15]. Genetic analysis was conducted in the first 5,000 study participants. For these, 3,463 individuals were genotyped in 2008 (GHS1) and further 1,439 individuals in 2009 (GHS2). Genotyping was performed for GHS1 and GHS2 using the Affymetrix Genome-Wide Human SNP Array 6.0 (http://www.affymetrix.com), as described by the Affymetrix user manual. Genotypes were called using the Affymetrix Birdseed-V2 calling algorithm. Individuals with low genotyping call rate, a too high level of heterozygosity (hetFDR>0.01)), with sex-mismatches, and with Non-European ancestry were excluded. After applying standard quality criteria (minor allele frequency >1%, genotype call rate >98% and P-value of deviation from Hardy-Weinberg equilibrium of >0.0001), 689,634 SNPs in 2996 individuals from GHS1 and 701,418 SNPs in 1,179 individuals from GHS2 remained for analysis (total 4175). Imputation of missing genotypes was performed using the software MACH (v1.0.18.c) and minimac (release 2012-03-14) with the reference panel 1000G Phase I Integrated Release Version 2 Haplotypes (2010-11 data freeze, 2012-02-14 haplotypes) for each cohort separately.

Generation R

Generation R Study, a population-based prospective cohort study of pregnant women and their children in Rotterdam, The Netherlands. A total of 9,778 pregnant women were included in the study. All children were born between April 2002 and January 2006 [16; 17]. The children were invited at age 5 years with their mothers for examination on the research center by trained nurses. Of the 9,778 included pregnant woman 6,690 participated with their children for physical examination in the research centre at 5 years of age. The study protocol was approved by the Medical Ethical Committee of the Erasmus Medical Centre, Rotterdam (MEC 217.595/2002/20). Written informed consent was obtained from all participants. Corneal curvature was obtained with a Zeiss IOL-master 500. Data were collected from right and left eyes. Three measurement of K1 and K2 were taken of OD and OS, and were averaged.

DNA from children (cord blood or during physical examination at 6 years of age) has been extracted, normalized and plated. Samples were genotyped using Illumina Infinium II HumanHap610 Quad Arrays following standard manufacturer's protocols. Intensity files were analyzed using the Beadstudio Genotyping Module software v.3.2.32, and genotype calling based on default cluster files. Any sample displaying call rates below 97.5%, excess of autosomal heterozygosity (F<mean-4SD) and mismatch between called and phenotypic gender (0.2%) were excluded. Genotypes were imputed for all polymorphic SNPs (single nucleotide polymorphisms) from phased haplotypes in autosomal chromosomes using the 1000 Genomes GIANTv3 panel.

Ogliastra Genetic Park, Talana study (OGP Talana)

A cross-sectional ophthalmic study was performed in Talana, Perdasdefogu and Urzulei within the Ogliastra Project, a large epidemiological survey conducted in a geographically, culturally and genetically isolated population living in an eastern-central region of Sardinia [26]. In Talana the study was carried out between October 2001 and October 2002 and adhered to the tenets of the declaration of Helsinki. Talana is an Ogliastran village situated at an altitude of 700 m above sea level in one of the most secluded areas of Sardinia; it has about 1200 inhabitants and, importantly, archival records are available from 1589 and genealogical trees have been reconstructed from 1640. 789 volunteers gave their written informed consent and were invited to the local medical centre, which was equipped with a complete set of ophthalmic instruments for this survey. All participants underwent a complete eye examination conducted according to a standardized protocol that included visual acuity measurement with Snellen charts at a distance of 5 m, autorefraction (RK-8100 Topcon, Tokyo, Japan) assessing sphere, cylinder and axis, slit lamp biomicroscopy (Model BQ900, Haag-Streit, Bern, Switzerland), contact tonometry and colour fundus photography (TRC-50IA,Topcon) and non-contact optical biometry (IOLMaster,Carl Zeiss, Italy) and Optical coherence tomography (OCT). Whole blood was obtained from all consenting family members of Talana village for DNA extraction. Genotyping was carried out using the Affymetrix 500k chips using standard protocols. SNPs quality control was performed using the GenABEL software package in R. Samples with overall SNP call rate < 95%, showing excess of heterozygosity, or being classified as outliers by allelic identity-by-state (IBS) clustering analysis, were excluded. After exclusion of SNPs

with minor allele frequency < 0.05, Hardy-Weinberg P value >10^{-4} and call rate < 95%, data were pre-phased with Shapeit and imputed with Impute2 Using the GIANT phase 1 release v3 1000 Genome reference panel.

RAINE Eye Health Study (RAINE)
The Raine Eye Health Study (REHS) was conceived to determine the prevalence of and risk factors for eye disease in young adults, and to characterize ocular biometric parameters in a young adult cohort [18]. The Western Australian Pregnancy Cohort (Raine) Study originated as a randomized-controlled trial of 2900 women recruited from the state's largest maternity hospital. Their offspring (N=2868) have been followed at birth, ages 1, 2, 3, 5, 8, 10, 14, 17 and 20 years of age in a prospective cohort study. DNA was collected from participants for genome-wide association studies and genotyping was performed using Illumina 660 Quad Array. Any pair of individuals who were related with a $\pi > 0.1875$ (in between second and third degree relatives – e.g. between half-sibs and cousins) was investigated, and the individual with the higher proportion of missing data was excluded from the 'clean' dataset (68 individuals excluded). Individuals who had low genotyping success (i.e. missing data) were excluded from the 'clean' dataset – a threshold of absent data > 3% was used for exclusion (16 individuals excluded). Additionally, if they had high levels of heterozygosity then they were also excluded (heterozygosity < 0.30 excluded 3 individuals). SNPs which did not satisfy a Hardy-Weinburg equilibrium p-value > 5.7x10-7 (919 markers), a call rate >95% (97,718 markers), and a minor allele frequency >0.01 (1%) (119,246 markers – includes CNV's) were excluded. To account for population stratification, the first five principal components were calculated using a subset of 42,888 SNPS that were not in LD with each other. Principal component analysis was conducted using the EIGENSTRAT program. Raine Study was imputed against the 1000 Genomes Phase 1 Europeans (November 23, 2010 release) using MACH v 2.3.0 software. A minimum passing threshold of 0.3 on the Rsq metric and a MAF>0.01 were applied to ~30 million imputed SNP. At the 20-year follow-up participants completed a comprehensive eye assessment that included visual acuity, orthoptic assessment and cycloplegic autorefraction, as well as several ocular biometric variables and multiple ophthalmic photographs of the anterior and posterior segments. Using the 20 year follow-up examination refractive error phenotypes, 348 Caucasian participants aged 20 years or older with high quality genotypes and known spherical equivalent refraction and educational level were included in the current analysis.

Rotterdam Study (RS1, RS2, RS3)
The Rotterdam Study is a prospective population-based cohort study in the elderly living in Ommoord, a suburb of Rotterdam, the Netherlands. Details of the study are described elsewhere [19]. In brief, the Rotterdam Study consists of 3 independent cohorts: RS1, RS2, and RS3. For the current analysis, 5,422 residents aged 55 years and older were included from RS1, 1,973 participants aged 55 and older from RS2, and 1,971 aged 45 and older from RS 3. 99% of subjects were of Caucasian ancestry. Participants underwent multiple physical examinations with regular intervals from 1991 to present, including a non-dilated automated measurement of refractive error using a Topcon RM-A2000 autorefractor. All measurements in RS-1–3 were conducted after the Medical Ethics Committee of the Erasmus University had approved the study protocols and all participants had given a written informed consent in accordance with the Declaration of Helsinki.

DNA was extracted from blood leucocytes according to standard procedures. Genotyping of SNPs was performed using the Illumina Infinium II HumanHap550 chip v3.0 array (RS-I); the HumanHap550 Duo Arrays and the Illumina Human610-Quad Arrays (RS-II), and the Human 610 Quad Arrays Illumina (RS-III). Samples with low call rate (<97.5%), with excess autosomal heterozygosity (>0.336), or with sex-mismatch were excluded, as were outliers identified by the identity-by-state clustering analysis (outliers were defined as being >3 s.d. from population mean or having identity-by-state probabilities >97%). We used genomic control to obtain optimal and unbiased results and applied the inverse variance method of each effect size estimated for both autosomal SNPs that were genotyped and imputed in both cohorts. A set of genotyped input SNPs with call rate >98%, with minor allele frequency >0.01, and with Hardy-Weinberg P value >10^{-6} was used for imputation. We used Minimac to impute to 1000G (phase 1, March 2012). For each imputed SNP, a reliability of imputation was estimated as the

ratio of the empirically observed dosage variance to the expected binomial dosage variance (O/E ratio).

UK biobank

Ethical approval for the UK Biobank study was obtained from the National Health Service (NHS) National Research Ethics committee (Ref. 11/NW/0382) and all participants provided informed consent. During 2006-2010 approximately 500,000 participants attended 1 of 22 assessment centres across the UK, where they completed interviews with a trained nurse and underwent a range of physical assessments. Approximately 25% of participants underwent an ophthalmic assessment, which included autorefraction and autokeratometry (Tomey RC5000; Tomey GmbH Europe, Erlangen-Tennenlohe, Germany). A logMAR visual acuity (VA) test was carried out at a test distance of 4 metres, with habitual spectacles if worn. A cohort profile describing the details of the UK Biobank eye measurements is available[20]

Participants who had undergone cataract surgery, retinal detachment surgery, or corneal surgery, or who self-reported a history of laser refractive surgery, cataract surgery, corneal graft surgery, any other eye surgery in the last 4 weeks, any eye trauma resulting in sight loss, serious eye problems, or self-report of having cataracts or retinal detachment were excluded. Exclusions were also made for: withdrawal of consent; genotype data not being released by UK Biobank [21]; non-European genetic ancestry; a mismatch between self-reported and genetically-inferred sex; genetic heterozygosity beyond 4 standard deviations of the mean level for Europeans in UK Biobank; no information for the trait or traits of interest (CC, refractive error, and VA); a missing genotype rate of 0.025 or more.

ASIAN ETHNICITY STUDIES

Beijing Eye Study (BES)

The BES is a population-based cohort of Han Chinese in the rural region and in the urban region of Beijing in North China. The Medical Ethics Committee of the Beijing Tongren Hospital approved the study protocol and all participants gave informed consent, according to the Declaration of Helsinki. At baseline (2001), 4439 individuals out of 5324 eligible individuals aged 40 years or older participated (response rate: 83.4%). In the years 2006 and 2011, the study was repeated by re-inviting all participants from the survey from 2001 to be re-examined. Out of the 4439 subjects examined in 2001, 3251 (73.2%) subjects returned for the follow-up examination in 2006, and 2695 (60.7%) subjects returned for the follow-up examination in 2011. For all subjects, visual acuity was measured. Automatic refractometry (Auto Refractometer AR-610, Nidek Co., Ltd, Tokyo, Japan) was performed if uncorrected visual acuity was lower than 1.0. The values obtained by automatic refractometry were verified and refined by subjective refractometry. Refraction data collected in 2011 was used in the analysis. In the survey of 2006, blood samples were taken from 2,929 (90.1%), and DNA was extracted from blood leucocytes according to standard procedures. We performed genotyping using Illumina Human610-Quad BeadChip in 988 subjects [22]. Of them, we excluded 151 with cryptic relatedness during sample QC procedure. Additional 259 Individuals with cataract surgery or missing refraction data were also excluded. This left a total of 585 individuals for analysis. Linear regression analyses for SE were performed at each SNP using 585 individuals with age, sex, and the first two principal components (to adjust for population stratification) included in the model.

Guangzhou Twin Eye Study (GTES)

The Guangzhou Twin Eye Study was launched in 2006, twins aged 7to 15 years at baseline were recruited and examined annually and it has completed 12 consecutive annual follow-up examinations, with more than 1300 twin pairs participating. Only the first-born twins were included in the current study. Those with manifest strabismus, amblyopia, nystagmus, post-refractive surgery, or any ocular disease causing best-corrected visual acuity less than 20/25 were excluded. The study was conducted in accordance with the tenets of the World Medical Association's Declaration of Helsinki and was approved by the Ethics Review Board of the Zhongshan Ophthalmic Center of Sun Yat-Sen University. Written informed consent was obtained from the parents or legal

guardians of the participants. Cycloplegia was induced with 2 drops of 1% cyclopentolate, administered 5 minutes apart, with a third drop administered after 20 minutes. Cycloplegia and pupil dilation were evaluated after an additional 15 minutes. Cycloplegia was considered complete if the pupil dilated to 6 mm or greater and a light reflex was absent. If not, another 20 minutes observation was taken, and refractive measurement was taken regardless of the presence or absence of light reflex. Corneal curvature was determined by an auto-refractor (Topcon KR-8800, Tokyo, Japan) after cycloplegia. The corneal curvature was recorded in diopter.

DNA was genotyped on the Affymetrix Genome-Wide ASI Axiom 1.0. Sample with high missing rate (>5%) were excluded. SNPs with calling rate > 95%, with minor allele frequency > 0.01 and with Hardy-Weinberg P-value > 0.0001 was used for imputation. We used Impute2 to impute to 1000G (phase 1, March 2012). The same QC methods used for the genotyped SNPs were applied to the GTES imputed SNPs. In addition, imputed SNPs with low imputation quality (**Score<0.5**) were excluded. Linear regression analyses of CC were performed using 1055 individuals with age, sex and the first two principal components (to adjust for population stratification) included in the model as covariates.

Hong Kong Study (Myopia Genomics Study of Hong Kong)
For the Myopia Genomics Study of Hong Kong, unrelated individuals of Chinese descent aged between 18 and 45 years were recruited via the Optometry Clinic at the Hong Kong Polytechnic University with details as described previously[23-25]. Ophthalmic examination was performed with refraction measured by cycloplegic autorefraction together with the measurement of other ocular components including corneal curvature, lens thickness and axial length. In particular, central corneal curvature was measured using autokeratometry (Canon RK-5 Auto Ref-keratometer; Canon, Inc, Tokyo). Subjects with signs of ocular disorders or other inherited disorders associated with myopia were excluded from the study. Whole-genome genotyping was performed using Illumina Human610 Quad BeadChips by deCODE Genetics (Iceland). The Human610 BeadChip assays ~621,000 SNPs and markers per sample.

Nagahama Prospective Genome Cohort for the Comprehensive Human Bioscience (Nagahama)
The Nagahama Prospective Genome Cohort for the Comprehensive Human Bioscience (the Nagahama Study) is a community-based prospective cohort study that aims to determine the prevalence and risk factors of various diseases in a community. The details of study design and methodology have been described elsewhere[26]. In brief, residents of Nagahama City who satisfied the following criteria were recruited as participants and were examined between November 2008 and November 2010: 1) age 30 and 74 years; 2) ability to participate on one's own; 3) no significant problems communicating in Japanese; 4) no current serious diseases/symptoms or health issues; and 5) voluntarily decided to participate in this study. A total of 9,804 Japanese individuals participated in the Nagahama Study. All the participants in the Nagahama Study had their axial length (millimeter [mm]; IOL Master, Carl Zeiss Meditec, Dublin, CA, USA), spherical equivalent (diopter [D]; ARK-530A, Nidek, Aichi, Japan), and corneal curvature (mm; ARK-530A, Nidek) measured for both eyes. Color fundus photographs were also obtained from all participants (CR-DG10, Canon, Tokyo, Japan). Of the participants, 3,712 individuals were genome-scanned using HumanHap610K Quad Arrays, HumanOmni2.5M Arrays, and/or HumanExome Arrays (Illumina Inc., San Diego, California, USA). After our standard quality control, genomic imputation was performed on 192 participants' data that had been genotyped by every platform. Finally, the data that consists of 1,756,611 SNPs of 3,248 individuals were fixed. All study procedures were approved by the ethics committee of Kyoto University Graduate School of Medicine.

Singapore Chinese Eye Study (SCES) I/II
Similar to SINDI, the Singapore Chinese Eye Study (SCES) is a population-based cross-sectional study of eye diseases in Chinese adults 40 years of age or older residing in the southwestern part of Singapore. The methodology of the SCES study has been described in detail previously. Between 2009 and 2011, 3,353 (72.8%) of 4,605 eligible individuals underwent a comprehensive ophthalmologic examination, using the same protocol as SINDI [27]. Genome-wide genotyping using was done in a subset of SCES participants using Illumina Human610-Quad BeadChip [22] (SCES I, n=1,952) and Illumina OmniExpress (SCES II, n = 615). Samples were excluded if

they showed evidence of admixture, cryptic relatedness, high heterogeneity and gender discrepancies. From a starting number of 1,952 individuals, three samples had per-sample call rate of <95% and were removed from analysis. A total of 21 individuals showed evidence of admixture and were consequently excluded. Biological relationship verification revealed a total of 29 sample pairs with cryptic relatedness. For these, the sample with the lower call rate was removed. In addition, further 14 samples with impossible biological sharing or heterogeneity, probably because of contamination, were removed, as well as two individuals who were removed due to gender discrepancies. PC analysis of the remaining individuals for SCES against the 1000 genomes phase 1 cosmopolitan panel haplotypes (March 2012 release) did not show the cohort to be dissimilar in ancestry, and therefore no PCs were used to correct for any underlying population substructure in the analysis performed. Individuals were excluded from the study if they had cataract surgery and missing refraction data. After phenotype and genotype QC, 1,662 individuals were left for the analysis. [22; 28].[29]

Singapore Malay Eye Study (SIMES)
SiMES is a population-based prevalence survey of Malay adults aged 40 to 79 years living in Singapore that was conducted between August of 2004 and June of 2006[27]. From a Ministry of Home Affairs random sample of 16,069 Malay adults in the Southwestern area, an age-stratified random sampling strategy was used in selecting 1400 from each decade from age 40 years onward (40–49, 50–59, 60–69, and 70–79 years).The 4,168 eligible participants from the sampling frame, while 3280 (78.7%) participated. Genome-wide genotyping was performed in 3,072 individuals [22; 28].

Total of 3,072 DNA samples were genotyped using the Illumina Human 610 Quad Beadchips [28; 30]. Using the same quality control criteria, we omitted a total of 530 individuals including those of subpopulation structure (n=170), cryptic relatedness (n=279), excessive heterozygosity or high missingness rate > 5% (n=37), and gender discrepancy (n=44). A total of 2165 individuals were over age 25 and had high quality genotypes and phenotypes for astigmatism. After the removal of the samples, SNP QC was then applied on a total of 579,999 autosomal SNPs for the 2,542 post-QC samples. The same QC methods used for SCES were applied to the SiMES genotyping samples. Linear regression analyses of CC were performed using 2,256 individuals with age, sex and the first two principal components (to adjust for population stratification) included in the model as covariates.

Singapore Indian Eye Study (SINDI)
SINDI is a population-based survey of major eye diseases [31] in ethnic Indians aged 40 to 80 years living in the South-Western part of Singapore and was conducted from August 2007 to December 2009. In brief, 4,497 Indian adults were eligible and 3,400 participated. Genome-wide genotyping was performed in 2,953 individuals [30]. Participants were excluded from the study if they had cataract surgery and missing refraction data.
The Illumina Human610 Quad Beadchips was used for genotyping all DNA samples from SINDI (n=2,593). We excluded 415 subjects from the total of 2,953 genotyped samples based on: excessive heterozygosity or high missingness rate > 5% (n=34) , cryptic relatedness (n=326), issues with population structure ascertainment (n=39) and gender discrepancies (n=16). This left a total of 2,538 individuals with 579,999 autosomal SNPs and 2,088 of these individuals were also over age 20 and had phenotype data. During SNP QC procedure. SNPs were excluded based on (i) high rates of missingness (> 5%) ; (ii) monomorphism or MAF < 1% ; or (iii) genotype frequencies deviated from HWE ($p < 1 \times 10^{-6}$). Linear regression analyses of CC were performed using 2,088 individuals with age, sex and the first two principal components (to adjust for population stratification) included in the model as covariates.

Singapore Prospective Study Program (SP2-1M; SP2-610)
Samples of SP2 were from a revisit of two previously conducted population-based surveys carried out in Singapore between 1992 and 1998, including the National Health Survey 1992 and the National Health Survey 1998[32]. These studies comprise random samplings of individuals stratified by ethnicity from the entire Singapore population. A total of 8266 subjects were invited in this follow-up survey and 6301 (76.1% response rate) subjects completed the questionnaire, of which 4056 (64.4% of those who completed the questionnaire) also attended the

health examination and donated blood specimens. The present GWA genotyping for SP2 involved individuals of Chinese descent only (n=2,867)[33].

Of the 2,867 blood-derived DNA samples, 1,459 samples were genotyped on the 610-Quad (SP2-610) and 1,016 samples on the 1M-Duov3 (SP2-1M). We excluded 443 individuals on the following conditions, sample call rates of less than 95%, excessive heterozygosity, cryptic relatedness by IBS, population structure ascertainment, and gender discrepancies as listed in the main text. During the SNPs QC procedure, we excluded SNPs with low genotyping call rates (> 5% missingness) or monomorphic, with MAF < 1%, or with significant deviation from HWE ($P < 10^{-6}$). This yielded a post-QC set of 462,580 SNPs. We additionally assessed the SNPs that are present on different platforms for extreme variations in allele frequencies with a 2-degree of freedom chi-square test of proportions, removing 62 SNPs with *P*-values < 0.0001. A total of 811 individuals in SP2-1M and 854 individuals in SP2-610 had both high quality genotype data and CC data and were used in the linear regression analyses adjusting for age and sex.

Strabismus, Amblyopia and Refractive Error Study (STARS)
The Strabismus, Amblyopia and Refractive Error Study in Singaporean Chinese Preschoolers (STARS) Family study is a family-based study nested in a prevalence survey of Singaporean preschool children (n=3,009) conducted from March 2008 to March 2010[34]. The biological parents of STARS probands were invited to enroll in the STARS Family study. A total of 1,451 samples from 440 nuclear fmailies were genotyped using Illumina Human610 Quad Beadchips. The 741 parents who had phenotype data and who also had available, high quality GWAS genotypes were used in the current study.

All Singapore studies adhere to the Declaration of Helsinki. Ethics approvals have been obtained from the Institutional Review Boards of the Singapore Eye Research Institute, Singapore General hospital, National University of Singapore and National Healthcare Group, Singapore. In all cohorts, participants provided written, informed consent at the recruitment into the studies.

Acknowledgements
We would like to acknowledge the following agencies and persons:

Core support for **ALSPAC** was provided by the UK Medical Research Council and Wellcome (Grant ref: 102215/2/13/2) and the University of Bristol. GWAS data was generated by Sample Logistics and Genotyping Facilities at Wellcome Sanger Institute and LabCorp (Laboratory Corporation of America) using support from 23andMe. This publication is the work of the authors and J.A. Guggenheim and C. Williams will serve as guarantors for the contents of this paper. This research was funded by the National Eye Research Centre, Bristol (SAC015); C.W. is supported by an NIHR Fellowship. ALSPAC thanks all the families who took part in this study, the midwives for their help in recruiting them, and the whole ALSPAC team, which includes interviewers, computer and laboratory technicians, clerical workers, research scientists, volunteers, managers, receptionists and nurses.

BATS/TEST (Australian Twins) were supported by an Australian National Health and Medical Research Council (NHMRC) Enabling Grant (2004-2009, 350415, 2005-2007); Clifford Craig Medical Research Trust; Ophthalmic Research Institute of Australia; American Health Assistance Foundation; Peggy and Leslie Cranbourne Foundation; Foundation for Children; Jack Brockhoff Foundation; National Institutes of Health/National Eye Institute (RO1EY01824601 (2007-2010)); Pfizer Australia Senior Research Fellowship (to D.A.M.); and Australian NHMRC Career Development Award (to S.M.). Genotyping was funded by an NHMRC Medical Genomics Grant; US National Institutes of Health/National Eye Institute (1RO1EY018246), Australian sample

imputation analyses were carried out on the Genetic Cluster Computer which is financially supported by the Netherlands Scientific Organization (NWO48005003). Australian Twins thank Nicholas Martin, Scott Gordon, Dale Nyholt, Sarah Medland, Brian McEvoy, Margaret Wright, Anjali Henders, Megan Campbell for ascertaining and processing genotyping data, and research nurses and managers at QIMR Berghofer for data collections on education level in the 19UP study; Jane MacKinnon, Shayne Brown, Lisa Kearns, Jonathan Ruddle, Paul Sanfilippo, Sandra Staffieri, Olivia Bigault, Colleen Wilkinson, Jamie Craig, Yaling Ma, Julie Barbour for assisting with clinical examinations; and Dr Camilla Day and staff at the Center for Inherited Disease Research.

BMES was supported by the Australian National Health & Medical Research Council (NH&MRC), Canberra Australia (974159, 211069, 457349, 512423, 475604, 529912); the Centre for Clinical Research Excellence in Translational Clinical Research in Eye Diseases; NH&MRC research fellowships (358702, 632909 to J.J.W, 1028444 and 1138585 to P.N.B.); and the Wellcome Trust, UK as part of Wellcome Trust Case Control Consortium 2 (A. Viswanathan, P. McGuffin, P. Mitchell, F. Topouzis, P. Foster) for genotyping costs of the entire BMES population (085475B08Z, 08547508Z, 076113). The Centre for Eye Research Australia receives Operational Infrastructure Support from the Victorian government. BMES acknowledges Elena Rochtchina from the Centre for Vision Research, Department of Ophthalmology and Westmead Millennium Institute University of Sydney (NSW Australia); John Attia, Rodney Scott, Elizabeth G. Holliday from the University of Newcastle (Newcastle, NSW Australia); Srujana Sahebjada and Andrea J. Richardson from the Centre for Eye Research Australia, University of Melbourne; Michael T. Inouye, Medical Systems Biology, Department of Pathology & Department of Microbiology & Immunology, University of Melbourne (Victoria, Australia); Ananth Viswanathan, Moorfields Eye Hospital (London, UK); Paul J. Foster, NIHR Biomedical Research Centre for Ophthalmology, UCL Institute of Ophthalmology & Moorfields Eye Hospital (London); Peter McGuffin, MRC Social Genetic and Developmental Psychiatry Research Centre, Institute of Psychiatry, King's College (London, United Kingdom); Fotis Topouzis, Department of Ophthalmology, School of Medicine, Aristotle University of Thessaloniki, AHEPA Hospital (Thessaloniki, Greece); Xueling Sim, National University of Singapore; members of the Wellcome Trust Case Control Consortium 2.

The **CROATIA** studies were funded by grants from the Medical Research Council (UK) and from the Republic of Croatia Ministry of Science, Education and Sports (10810803150302); and the CROATIA-Korcula genotyping was funded by the European Union framework program 6 project EUROSPAN (LSHGCT2006018947). The CROATIA studies acknowledges Dr. Goran Bencic, Biljana Andrijević Derk, Valentina Lacmanović Lončar, Krešimir Mandić, Antonija Mandić, Ivan Škegro, Jasna Pavičić Astaloš, Ivana Merc, Miljenka Martinović, Petra Kralj, Tamara Knežević and Katja Barać-Juretić as well as the recruitment team from the Croatian Centre for Global Health, University of Split and the Institute of Anthropological Research in Zagreb for the ophthalmological data collection; Peter Lichner and the Helmholtz Zentrum Munchen (Munich, Germany), AROS Applied Biotechnology, Aarhus, Denmark and the Wellcome Trust Clinical facility (Edinburgh, United Kingdom) for the SNP genotyping all studies; Jennifer Huffman, Susan Campbell and Pau Navarro for genetic data preparation. Research was supported by the MRC Human Genetics Unit core programme grant "QTL in Health and Disease", currently MC_UU_0007/10.

The **DCCT/**EDIC Research Group is sponsored through research contracts from the National Institute of Diabetes, Endocrinology and Metabolic Diseases of the National Institute of Diabetes and Digestive and Kidney Diseases (NIDDK) and the National Institutes of Health. Clinical data and DNA from the DCCT study is available through the National Institute of Diabetes and Digestive and Kidney Diseases repository at https://www.niddkrepository.org/niddk/home.do. The authors are grateful to the patients and researchers of DCCT/EDIC. ADP is the guarantor for DCCT.
EGCUT studies were financed by University of Tartu (grant "Center of Translational Genomics") by Estonian Government (grant # TP1GV140601, grant #ETF9353) and by European Commission through the European Regional Development Fund in the frame of grant "center of Excellence in genomic" and by an Estonian Reseach Infrastructure's Roadmap and through FP7 grant #313010.

EPIC-Norfolk infrastructure and core functions are supported by grants from the Medical Research Council (G1000143) and Cancer Research UK (C864/A14136). The clinic for the third health examination was funded by Research into Ageing (262). Mr Khawaja is funded by a Moorfields Eye Charity Career Development Fellowship. Professor Foster has received additional support from the Richard Desmond Charitable Trust (via Fight for Sight) and the Department for Health through the award made by the National Institute for Health Research to Moorfields Eye Hospital and the UCL Institute of Ophthalmology for a specialist Biomedical Research Centre for Ophthalmology.

FITSA was supported by ENGAGE (FP7-HEALTH-F4-2007, 201413); European Union through the GENOMEUTWIN project (QLG2-CT-2002-01254); the Academy of Finland Center of Excellence in Complex Disease Genetics (213506, 129680); the Academy of Finland Ageing Programme; and the Finnish Ministry of Culture and Education and University of Jyväskylä. For FITSA the contributions of Antti-Pekka Sarin, Emmi Tikkanen, Samuli Ripatti, Markku Kauppinen, Taina Rantanen and Jaakko Kaprio are acknowledged.

The **Gutenberg Health Study** is funded through the government of Rheinland-Pfalz ("Stiftung Rheinland Pfalz für Innovation" (contract AZ 961-386261/733); the research programs "Wissen schafft Zukunft" and “Center for Translational Vascular Biology (CTVB)” of the Johannes Gutenberg-University of Mainz and its contract with Boehringer Ingelheim and PHILIPS Medical Systems, including unrestricted grants for the Gutenberg Health Study. GHS acknowledges Dagmar Laubert-Reh for data analysis, and thanks all study participants for their willingness to provide data for this research project and we are indebted to all coworkers for their enthusiastic commitment.

OGP Talana was supported by grants from the Italian Ministry of Education, University and Research (5571DSPAR2002, 718Ric2005). OGP Talana thanks the Ogliastra population and the municipal administrators for their collaboration to the project and for economic and logistic support.

ORCADES was supported by the Chief Scientist Office of the Scottish Government, the Royal Society, the Medical Research Council Human Genetics Unit and the European Union framework program 6 EUROSPAN project (LSHGCT2006018947). ORCADES acknowledges the invaluable contributions of Lorraine Anderson and the research nurses in Orkney, in particular Margaret Pratt who performed the eye measurements,as well as the administrative team in Edinburgh University; and the Wellcome Trust Clinical facility (Edinburgh, United Kingdom) for DNA extraction; and Peter Lichner and the Helmholtz Zentrum Munchen (Munich, Germany) for genotyping; and Mirna Kirin for the genetic data imputation.

The core management of the **RAINE** Study is funded by The University of Western Australia (UWA), The Telethon Institute for Child Health Research, Raine Medical Research Foundation, UWA Faculty of Medicine, Dentistry and Health Sciences, Women’s and Infant’s Research Foundation and Curtin University. Genotyping was funded by NHMRC project grant 572613. Support for the Raine Eye Health Study was provided by NHMRC Grant 1021105, Lions Eye Institute, the Australian Foundation for the Prevention of Blindness and Alcon Research Institute. The Raine Eye Health Study authors thank the Raine eye health study participants and their families. They also thank the Raine Study management, Craig Pennell, Jenny Mountain and the team at TICHR and LEI for cohort co-ordination and data collection, particularly: Seyhan Yazar, Hannah Forward,Charlotte McKnight, Alex Tan, Alla Soloshenko, Sandra Oates, and Diane Wood.

The **Generation R** Study is conducted by the Erasmus Medical Centre in close collaboration with the School of Law and Faculty of Social Sciences of the Erasmus University Rotterdam, the Municipal Health Service Rotterdam area, Rotterdam, the Rotterdam Homecare Foundation, Rotterdam and the Stichting Trombosedienst & Artsenlaboratorium Rijnmond (STAR-MDC), Rotterdam. We gratefully acknowledge the contribution of children and parents, general practitioners, hospitals, midwives and pharmacies in Rotterdam. The Generation R study is made possible by financial support from the Erasmus Medical Centre, Rotterdam; the Netherlands Organisation

for Scientific Research (NWO); the Netherlands Organisation for Health Research and Development (ZonMw); the Dutch Ministry of Education, Culture and Science; the Dutch Ministry of Health, Welfare, and Sports; the European Commission (DG XII). The research was funded by the European Research Council (ERC) under the European Union's Horizon 2020 research and innovation programme (grant 648268) and the Netherlands Organisation for Scientific Research (NWO, grant 91815655) The author was supported by the following foundations: Algemene Nederlandse Vereniging ter Voorkoming van Blindheid, De Landelijke Stichting voor Blinden en Slechtzienden, MaculaFonds, Novartis Researchfunds, ODAS Stichting en het Oogfonds that contributed through UitZicht (Grant 2013-24) and the Henkes Stichting. The funding organizations had no role in the design or conduct of this research. They provided unrestricted grants

The **Rotterdam Study** and **ERF** are supported by the Netherlands Organisation of Scientific Research NWO Investments (nr. 175.010.2005.011, 911-03-012, Vidi nr. 91796357 to C.C.W.K.); the Research Institute for Diseases in the Elderly (014-93-015; RIDE2), the Netherlands Genomics Initiative (NGI)/Netherlands Organisation for Scientific Research (NWO) project nr. 050-060-810; Erasmus Medical Center and Erasmus University, Rotterdam, The Netherlands; Netherlands Organization for Health Research and Development (ZonMw); UitZicht; the Ministry of Education, Culture and Science; the Ministry for Health, Welfare and Sports; the European Commission (DG XII); the Municipality of Rotterdam; Center for Medical Systems Biology of NGI; Lijf en Leven; M.D. Fonds; Henkes Stichting; Stichting Nederlands Oogheelkundig Onderzoek; Swart van Essen; Bevordering van Volkskracht; Blindenhulp; Landelijke Stichting voor Blinden en Slechtzienden; Rotterdamse Vereniging voor Blindenbelangen; OOG; Algemene Nederlandse Vereniging ter Voorkoming van Blindheid; the Rotterdam Eye Hospital Research Foundation; ErasmusMC Trustfonds; Stichting Glaucoomfonds; Stichting Nederlands Oogheelkundig Onderzoek (SNOO); Ter Meulen Fonds; Stichting tot Verbetering Lot van de Blinden; Stichting Dondersfonds; MolMed; LSBS; Stichting Nelly Reef Fund; Stichting Simonsfonds; Novartis; Alcon; Topcon Europe. We thank Pascal Arp, Mila Jhamai, Marijn Verkerk, Lizbeth Herrera and Marjolein Peters, Patricia van Hilten, Jeanette Vergeer, Sander Bervoets for their help in creating the GWAS database, and Karol Estrada and Maksim V. Struchalin for their support in creation and analysis of imputed data. We thank Ada Hooghart, Corina Brussee, Riet Bernaerts-Biskop for the ophthalmological examinations. The authors are grateful to the study participants, the staff from the Rotterdam Study and the participating general practitioners and pharmacists.

UK Biobank was established by the Wellcome Trust; the UK Medical Research Council; the Department for Health (London, UK); Scottish Government (Edinburgh, UK); and the Northwest Regional Development Agency (Warrington, UK). It also received funding from the Welsh Assembly Government (Cardiff, UK); the British Heart Foundation; and Diabetes UK. Collection of eye and vision data was supported by The Department for Health through an award made by the NIHR to the Biomedical Research Centre at Moorfields Eye Hospital NHS Foundation Trust, and UCL Institute of Ophthalmology, London, United Kingdom (grant no. BRC2_009). Additional support was provided by The Special Trustees of Moorfields Eye Hospital, London, United Kingdom (grant no. ST 12 09). Data analysis was carried out using the RAVEN computing cluster, maintained by the ARCCA group at Cardiff University ARCCA and the BLUE CRYSTAL3 computing cluster maintained by the HPC group at the University of Bristol. This research has been conducted using the UK Biobank Resource (applications #17351).

Nagahama Study was supported by Grants-in-aid for scientific research (No. 2429082, No24592624) from the Japan Society for the Promotion of Science, Tokyo, Japan, Health Labour Sciences Research Grant H23-003, and Supporting Program for Interaction-based Initiative Team Studies.

Beijing Eye Study was supported by National Natural Science Foundation of China (grant 81170890).

The **GTES** was supported by National Natural Science Foundation of China (grant 11771463) and Peal River S&T Nova Program (Grant 201806010142)

Hong Kong Study (Myopia Genomics Study of Hong Kong) was supported by grants from the Hong Kong Polytechnic University (J-BB7P, G-YBK2, G-YBPJ and 99QP) and the Research Grant Council of Hong Kong (B-Q33T and B-Q45J). Prof. Maurice Yap was supported by the Endowed Professorship Scheme (KB Woo Family Endowed Professorship in Optometry) of the Hong Kong Polytechnic University.

The Singapore studies (**SCES-I/II, SIMES, SINDI, SP2-1M/610** were supported by the National Medical Research Council, Singapore (NMRC 0796/2003, NMRC 1176/2008, STaR/0003/2008; CG/SERI/2010), Biomedical Research Council, Singapore (06/1/21/19/466, 09/1/35/19/616 and 08/1/35/19/550). The Singapore Tissue Network and the Genome Institute of Singapore, Agency for Science, Technology and Research, Singapore provided services.
The Strabismus, Amblyopia, and Refractive Error Study of Preschool Children (**STARS**) was supported by a NMRC grant (1176/2008).The animal work was supported by the grant from the National Medical Research Council, Singapore (NMRC/IRG/1117/2008; NMRC/CG/SERI/T1/2010; NMRC/CG/015/2013) to V.A.B. The authors appreciate the support of Duke-NUS/ SingHealth Academic Medicine Research Institute and the medical editing assistance of Taara Madhavan (Associate, Clinical Sciences, Duke-NUS Graduate Medical School).

JEBW, DDL, QL, CDM and AMM were supported by the Intramural Research Program of the National Human Genome Research Institute, National Institutes of Health, USA.

Founding Resources for TLY include University of Wisconsin Centennial Scholars Program and National Institutes of Health, National Eye Institute 2R01EY014685-11A1.

References

1. Boyd, A., Golding, J., Macleod, J., Lawlor, D.A., Fraser, A., Henderson, J., Molloy, L., Ness, A., Ring, S., and Davey Smith, G. (2013). Cohort Profile: The 'Children of the 90s'--the index offspring of the Avon Longitudinal Study of Parents and Children. Int J Epidemiol 42, 111-127.
2. Fraser, A., Macdonald-Wallis, C., Tilling, K., Boyd, A., Golding, J., Davey Smith, G., Henderson, J., Macleod, J., Molloy, L., Ness, A., et al. (2013). Cohort Profile: the Avon Longitudinal Study of Parents and Children: ALSPAC mothers cohort. Int J Epidemiol 42, 97-110.
3. Mackey, D.A., Mackinnon, J.R., Brown, S.A., Kearns, L.S., Ruddle, J.B., Sanfilippo, P.G., Sun, C., Hammond, C.J., Young, T.L., Martin, N.G., et al. (2009). Twins eye study in Tasmania (TEST): rationale and methodology to recruit and examine twins. Twin Res Hum Genet 12, 441-454.
4. Foran, S., Wang, J.J., and Mitchell, P. (2003). Causes of visual impairment in two older population cross-sections: the Blue Mountains Eye Study. Ophthalmic Epidemiol 10, 215-225.
5. Vitart, V., Bencic, G., Hayward, C., Herman, J.S., Huffman, J., Campbell, S., Bucan, K., Zgaga, L., Kolcic, I., Polasek, O., et al. (2010). Heritabilities of ocular biometrical traits in two croatian isolates with extended pedigrees. Invest Ophthalmol Vis Sci 51, 737-743.
6. Delaneau, O., Zagury, J.F., and Marchini, J. (2013). Improved whole-chromosome phasing for disease and population genetic studies. Nat Methods 10, 5-6.
7. Howie, B.N., Donnelly, P., and Marchini, J. (2009). A flexible and accurate genotype imputation method for the next generation of genome-wide association studies. PLoS Genet 5, e1000529.
8. Riboli, E., and Kaaks, R. (1997). The EPIC Project: rationale and study design. European Prospective Investigation into Cancer and Nutrition. Int J Epidemiol 26 Suppl 1, S6-14.
9. Day, N., Oakes, S., Luben, R., Khaw, K.T., Bingham, S., Welch, A., and Wareham, N. (1999). EPIC-Norfolk: study design and characteristics of the cohort. European Prospective Investigation of Cancer. Br J Cancer 80 Suppl 1, 95-103.
10. Hayat, S.A., Luben, R., Keevil, V.L., Moore, S., Dalzell, N., Bhaniani, A., Khawaja, A.P., Foster, P., Brayne, C., Wareham, N.J., et al. (2014). Cohort profile: A prospective cohort study of objective physical and cognitive capability and visual health in an ageing population of men and women in Norfolk (EPIC-Norfolk 3). Int J Epidemiol 43, 1063-1072.
11. Khawaja, A.P., Chan, M.P., Hayat, S., Broadway, D.C., Luben, R., Garway-Heath, D.F., Sherwin, J.C., Yip, J.L., Dalzell, N., Wareham, N.J., et al. (2013). The EPIC-Norfolk Eye Study: rationale, methods and a cross-sectional analysis of visual impairment in a population-based cohort. BMJ Open 3.
12. Aulchenko, Y.S., Heutink, P., Mackay, I., Bertoli-Avella, A.M., Pullen, J., Vaessen, N., Rademaker, T.A., Sandkuijl, L.A., Cardon, L., Oostra, B., et al. (2004). Linkage disequilibrium in young genetically isolated Dutch population. Eur J Hum Genet 12, 527-534.
13. Pardo, L.M., MacKay, I., Oostra, B., van Duijn, C.M., and Aulchenko, Y.S. (2005). The effect of genetic drift in a young genetically isolated population. Ann Hum Genet 69, 288-295.
14. Parssinen, O., Jauhonen, H.M., Kauppinen, M., Kaprio, J., Koskenvuo, M., and Rantanen, T. (2010). Heritability of spherical equivalent: a population-based twin study among 63- to 76-year-old female twins. Ophthalmology 117, 1908-1911.
15. Zeller, T., Wild, P., Szymczak, S., Rotival, M., Schillert, A., Castagne, R., Maouche, S., Germain, M., Lackner, K., Rossmann, H., et al. (2010). Genetics and beyond--the transcriptome of human monocytes and disease susceptibility. PLoS One 5, e10693.
16. Jaddoe, V.W., van Duijn, C.M., Franco, O.H., van der Heijden, A.J., van Iizendoorn, M.H., de Jongste, J.C., van der Lugt, A., Mackenbach, J.P., Moll, H.A., Raat, H., et al. (2012). The Generation R Study: design and cohort update 2012. Eur J Epidemiol 27, 739-756.
17. Jaddoe, V.W., Bakker, R., van Duijn, C.M., van der Heijden, A.J., Lindemans, J., Mackenbach, J.P., Moll, H.A., Steegers, E.A., Tiemeier, H., Uitterlinden, A.G., et al. (2007). The Generation R Study Biobank: a resource for epidemiological studies in children and their parents. Eur J Epidemiol 22, 917-923.
18. Yazar, S., Forward, H., McKnight, C.M., Tan, A., Soloshenko, A., Oates, S.K., Ang, W., Sherwin, J.C., Wood, D., Mountain, J.A., et al. (2013). Raine eye health study: design, methodology and baseline

prevalence of ophthalmic disease in a birth-cohort study of young adults. Ophthalmic genetics 34, 199-208.
19. Hofman, A., van Duijn, C.M., Franco, O.H., Ikram, M.A., Janssen, H.L., Klaver, C.C., Kuipers, E.J., Nijsten, T.E., Stricker, B.H., Tiemeier, H., et al. (2011). The Rotterdam Study: 2012 objectives and design update. Eur J Epidemiol 26, 657-686.
20. Chua, S.Y.L., Thomas, D., Allen, N., Lotery, A., Desai, P., Patel, P., Muthy, Z., Sudlow, C., Peto, T., Khaw, P.T., et al. (2019). Cohort profile: design and methods in the eye and vision consortium of UK Biobank. BMJ Open 9, e025077.
21. Bycroft, C., Freeman, C., Petkova, D., Band, G., Elliott, L.T., Sharp, K., Motyer, A., Vukcevic, D., Delaneau, O., O'Connell, J., et al. (2018). The UK Biobank resource with deep phenotyping and genomic data. Nature 562, 203-209.
22. Cornes, B.K., Khor, C.C., Nongpiur, M.E., Xu, L., Tay, W.T., Zheng, Y., Lavanya, R., Li, Y., Wu, R., Sim, X., et al. (2012). Identification of four novel variants that influence central corneal thickness in multi-ethnic Asian populations. Hum Mol Genet 21, 437-445.
23. Jiang, B., Yap, M.K., Leung, K.H., Ng, P.W., Fung, W.Y., Lam, W.W., Gu, Y.S., and Yip, S.P. (2011). PAX6 haplotypes are associated with high myopia in Han chinese. PLoS One 6, e19587.
24. Zha, Y., Leung, K.H., Lo, K.K., Fung, W.Y., Ng, P.W., Shi, M.G., Yap, M.K., and Yip, S.P. (2009). TGFB1 as a susceptibility gene for high myopia: a replication study with new findings. Arch Ophthalmol 127, 541-548.
25. Mak, J.Y., Yap, M.K., Fung, W.Y., Ng, P.W., and Yip, S.P. (2012). Association of IGF1 gene haplotypes with high myopia in Chinese adults. Arch Ophthalmol 130, 209-216.
26. Nakata, I., Yamashiro, K., Nakanishi, H., Akagi-Kurashige, Y., Miyake, M., Tsujikawa, A., Matsuda, F., Yoshimura, N., and Nagahama Cohort Research, G. (2013). Prevalence and characteristics of age-related macular degeneration in the Japanese population: the Nagahama study. Am J Ophthalmol 156, 1002-1009 e1002.
27. Foong, A.W., Saw, S.M., Loo, J.L., Shen, S., Loon, S.C., Rosman, M., Aung, T., Tan, D.T., Tai, E.S., and Wong, T.Y. (2007). Rationale and methodology for a population-based study of eye diseases in Malay people: The Singapore Malay eye study (SiMES). Ophthalmic Epidemiol 14, 25-35.
28. Vithana, E.N., Aung, T., Khor, C.C., Cornes, B.K., Tay, W.T., Sim, X., Lavanya, R., Wu, R., Zheng, Y., Hibberd, M.L., et al. (2011). Collagen-related genes influence the glaucoma risk factor, central corneal thickness. Hum Mol Genet 20, 649-658.
29. Lawlor, D.A., Harbord, R.M., Sterne, J.A., Timpson, N., and Davey Smith, G. (2008). Mendelian randomization: using genes as instruments for making causal inferences in epidemiology. Stat Med 27, 1133-1163.
30. Khor, C.C., Ramdas, W.D., Vithana, E.N., Cornes, B.K., Sim, X., Tay, W.T., Saw, S.M., Zheng, Y., Lavanya, R., Wu, R., et al. (2011). Genome-wide association studies in Asians confirm the involvement of ATOH7 and TGFBR3, and further identify CARD10 as a novel locus influencing optic disc area. Hum Mol Genet 20, 1864-1872.
31. Lavanya, R., Jeganathan, V.S., Zheng, Y., Raju, P., Cheung, N., Tai, E.S., Wang, J.J., Lamoureux, E., Mitchell, P., Young, T.L., et al. (2009). Methodology of the Singapore Indian Chinese Cohort (SICC) eye study: quantifying ethnic variations in the epidemiology of eye diseases in Asians. Ophthalmic Epidemiol 16, 325-336.
32. Hughes, K., Aw, T.C., Kuperan, P., and Choo, M. (1997). Central obesity, insulin resistance, syndrome X, lipoprotein(a), and cardiovascular risk in Indians, Malays, and Chinese in Singapore. J Epidemiol Community Health 51, 394-399.
33. Sim, X., Ong, R.T., Suo, C., Tay, W.T., Liu, J., Ng, D.P., Boehnke, M., Chia, K.S., Wong, T.Y., Seielstad, M., et al. (2011). Transferability of type 2 diabetes implicated loci in multi-ethnic cohorts from Southeast Asia. PLoS Genet 7, e1001363.
34. Li, L.J., Cheung, C.Y., Gazzard, G., Chang, L., Mitchell, P., Wong, T.Y., and Saw, S.M. (2011). Relationship of ocular biometry and retinal vascular caliber in preschoolers. Investigative ophthalmology & visual science 52, 9561-9566.
