# Supplementary material for: Genome-wide association meta-analysis of corneal curvature identifies novel loci and shared genetic influences across axial length and refractive error
Source: Commun Biol. 2020 Mar 19;3:133. doi: 10.1038/s42003-020-0802-y (PMC7081241; doi:10.1038/s42003-020-0802-y)
Supplement: Supplementary file 2 — Description of Additional Supplementary Files [file 42003_2020_802_MOESM2_ESM.docx]

**Supplementary Data:**

Supplementary Data 1. GWAS hits for corneal curvature by age group

Supplementary Data 2. Annotation and function characterization of variants in LD at the 41 loci

Supplementary Data 3. Association of corneal curvature SNPs with axial length and spherical equivalent

Supplementary Data 4. Significant pathways enriched using VEGAS

Supplementary Data 5. Significant pathways enriched for implicated corneal curvature genes using g:Profile

Supplementary Data 6. Expression of genes at 41 loci in the ocular tissue database

Supplementary Data 7. eQTL queries using GTEx gene expression in various tissues

Supplementary Data 8. Summary association statistical results that support the findings of this study.
