# Supplementary material for: Genome-wide association meta-analysis of corneal curvature identifies novel loci and shared genetic influences across axial length and refractive error
Source: Commun Biol. 2020 Mar 19;3:133. doi: 10.1038/s42003-020-0802-y (PMC7081241; doi:10.1038/s42003-020-0802-y)
Supplement: Supplementary file 11 — Reporting Summary [file 42003_2020_802_MOESM11_ESM.pdf]

nature research

Corresponding author(s): Qiao Fan, Ching-Yu Cheng

Last updated by author(s): 01/8/2020

Reporting Summary

Nature Research wishes to improve the reproducibility of the work that we publish. This form provides structure for consistency and transparency in reporting. For further information on Nature Research policies, see Authors & Referees and the Editorial Policy Checklist.

Statistics

For all statistical analyses, confirm that the following items are present in the figure legend, table legend, main text, or Methods section.

n/a	Confirmed	
☐	☒	The exact sample size (*n*) for each experimental group/condition, given as a discrete number and unit of measurement
☐	☒	A statement on whether measurements were taken from distinct samples or whether the same sample was measured repeatedly
☐	☒	The statistical test(s) used AND whether they are one- or two-sided<br>*Only common tests should be described solely by name; describe more complex techniques in the Methods section.*
☐	☒	A description of all covariates tested
☐	☒	A description of any assumptions or corrections, such as tests of normality and adjustment for multiple comparisons
☐	☒	A full description of the statistical parameters including central tendency (e.g. means) or other basic estimates (e.g. regression coefficient) AND variation (e.g. standard deviation) or associated estimates of uncertainty (e.g. confidence intervals)
☐	☒	For null hypothesis testing, the test statistic (e.g. F, t, r) with confidence intervals, effect sizes, degrees of freedom and P value noted<br>*Give P values as exact values whenever suitable.*
☒	☐	For Bayesian analysis, information on the choice of priors and Markov chain Monte Carlo settings
☒	☐	For hierarchical and complex designs, identification of the appropriate level for tests and full reporting of outcomes
☐	☒	Estimates of effect sizes (e.g. Cohen's *d*, Pearson's *r*), indicating how they were calculated

Our web collection on statistics for biologists contains articles on many of the points above.

Software and code

Policy information about availability of computer code

Data collection	Each study collected data individually with the method/software described in the supplementary file.
Data analysis	Different softwares have been used for data analysis for each study. Meta-analysis and other related softwares used for centralized analysis were listed in the main paper

For manuscripts utilizing custom algorithms or software that are central to the research but not yet described in published literature, software must be made available to editors/reviewers. We strongly encourage code deposition in a community repository (e.g. GitHub). See the Nature Research guidelines for submitting code & software for further information.

Data

Policy information about availability of data

All manuscripts must include a data availability statement. This statement should provide the following information, where applicable:

- Accession codes, unique identifiers, or web links for publicly available datasets
- A list of figures that have associated raw data
- A description of any restrictions on data availability

The authors declare that data supporting the findings of this study are available within the paper and supplementary tables. The datasets generated during and/or analysed during the current study are available from the corresponding authors on reasonable request.

Field-specific reporting

Please select the one below that is the best fit for your research. If you are not sure, read the appropriate sections before making your selection.

☒ Life sciences ☐ Behavioural & social sciences ☐ Ecological, evolutionary & environmental sciences

For a reference copy of the document with all sections, see nature.com/documents/nr-reporting-summary-flat.pdf

Life sciences study design

All studies must disclose on these points even when the disclosure is negative.

Sample size	For GWAS we included all available samples in the analysis to boost power as genetic effects of variants are moderate .
Data exclusions	For each study the samples with missing phenotypes were excluded, as well as those with missing genotypes >5%. Different study conducted QC procedure respectively. We also excluded rare variants with MAF < 0.01, departure from HWE (P < 10^6) and poorly imputed markers (IMPUTE info < 0.5) at the meta-analysis stage.
Replication	We validated and replicated our findings in UK Biobank data.
Randomization	Randomization is not relevant for GWAS study. To account for potential confounding of population stratification, we adjusted top PC components in GWAS. In meta-analysis, we adjusted p-values by gnomic control factor (lambda).
Blinding	No blinding is implemented in this study as it is not a RCT. We used summary statistics for meta-analysis.

Reporting for specific materials, systems and methods

We require information from authors about some types of materials, experimental systems and methods used in many studies. Here, indicate whether each material, system or method listed is relevant to your study. If you are not sure if a list item applies to your research, read the appropriate section before selecting a response.

Materials & experimental systems

n/a	Involved in the study	
☒	☐	Antibodies
☒	☐	Eukaryotic cell lines
☒	☐	Palaeontology
☒	☐	Animals and other organisms
☐	☒	Human research participants
☒	☐	Clinical data

Methods

n/a	Involved in the study	
☒	☐	ChIP-seq
☒	☐	Flow cytometry
☒	☐	MRI-based neuroimaging

Human research participants

Policy information about studies involving human research participants

Population characteristics	Our studies included individuals with European ancestry (n= 29,580) and Asian ancestry (n= 14,462) from 28 studies. We further replicated the findings in 88,218 participants from UK Biobank. Detailed characteristics of participants of each study were described in Supplementary Notes and Supplementary Table 1.
Recruitment	All participating CREAM cohorts used similar protocols for the collection of keratometry and other ocular biometric measurements. In general, majority of studies (total of 28 studies) are population-based studies. The participants with conditions that could alter refraction were excluded.
Ethics oversight	All studies were performed with the approval of their Human Research and Ethics Committee. Written informed consent was obtained from all participants in accordance with the Declaration of Helsinki.

Note that full information on the approval of the study protocol must also be provided in the manuscript.
